# Whi5 phosphorylation embedded in the G_1_/S network dynamically controls critical cell size and cell fate

**DOI:** 10.1038/ncomms11372

**Published:** 2016-04-20

**Authors:** Pasquale Palumbo, Marco Vanoni, Valerio Cusimano, Stefano Busti, Francesca Marano, Costanzo Manes, Lilia Alberghina

**Affiliations:** 1 SYSBIO.IT Center for Systems Biology, Italy; 2 CNR-IASI, Italian National Research Council - Institute for Systems Analysis and Computer Science, Via dei Taurini 19, 00185 Rome, Italy; 3 Department of Biotechnology and Biosciences, University of Milano-Bicocca, Piazza della Scienza 2, 20126 Milan, Italy; 4 Department of Information Engineering, Computer Science and Mathematics, University of L'Aquila, Via Vetoio, 67100 Coppito (L'Aquila), Italy

## Abstract

In budding yeast, overcoming of a critical size to enter S phase and the mitosis/mating switch—two central cell fate events—take place in the G_1_ phase of the cell cycle. Here we present a mathematical model of the basic molecular mechanism controlling the G_1_/S transition, whose major regulatory feature is multisite phosphorylation of nuclear Whi5. Cln3–Cdk1, whose nuclear amount is proportional to cell size, and then Cln1,2–Cdk1, randomly phosphorylate both decoy and functional Whi5 sites. Full phosphorylation of functional sites releases Whi5 inhibitory activity, activating G_1_/S transcription. Simulation analysis shows that this mechanism ensures coherent release of Whi5 inhibitory action and accounts for many experimentally observed properties of mitotically growing or conjugating G_1_ cells. Cell cycle progression and transcriptional analyses of a Whi5 phosphomimetic mutant verify the model prediction that coherent transcription of the G_1_/S regulon and ensuing G_1_/S transition requires full phosphorylation of Whi5 functional sites.

## Abstract (web summary)

In budding yeast the G1/S transition requires the attainment of a critical cell size. Here the authors unravel its basic control mechanism by integrating simulations of a mathematical model of multisite phosphorylation of Whi5 by Cln3–Cdk1, with molecular analyses of a Whi5 phospho-mimetic mutant.

## Graphical abstract

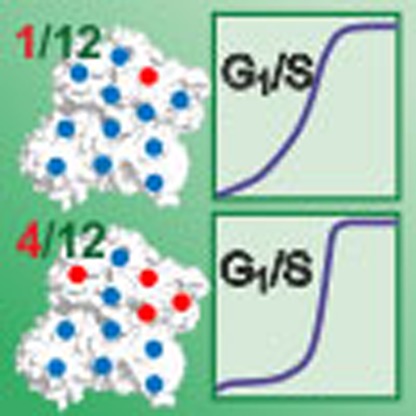

The ability of G_1_ cells to select specific cell fates in response to external and internal cues is crucial for life, in both lower and higher eukaryotes^1^. In the presence of nutrients—and in the absence of the complementary mating factors—haploid G_1_ cells of the budding yeast *Saccharomyces cerevisiae—*a widely used model for the study of the eukaryotic cell cycle—grow to the critical cell size required to enter S phase^2^,^3^ and are committed to proliferation. The complementary mating factor induces haploid G_1_ cells to undergo cell cycle arrest, differentiate and conjugate producing diploid cells^4^,^5^. Nutrient-limiting conditions lead to growth arrest and entry into stationary phase^6^,^7^.

Several features of critical cell size control have been reported. The average cell size of yeast populations increases with ploidy^3^,^8^. Smaller G_1_ cells require a longer G_1_ phase before entering S phase as compared to larger G_1_ cells^9^,^10^,^11^,^12^,^13^. The large size variability of newborn daughter cells is substantially reduced when they enter S phase^13^. Cells exponentially growing in rich media supporting faster growth rate have a larger critical cell size than that of cells growing in poor media which support slower growth^11^,^14^. During a nutritional shift up from a poor to a rich medium, changes in growth rate and cycle progression cause the average cell size of the population to increase, slowly adapting to the size characteristic of the new medium^15^. Finally, several signalling pathways modulate critical cell size^16^,^17^,^18^,^19^,^20^,^21^,^22^,^23^.

The number of gene products involved in execution and control of growth and cell cycle has been estimated at around 300 for the cell cycle and around 600 for growth^24^,^25^. Gene products reported to modulate the critical cell size include proteins involved in the G_1_/S transition network (such as Cln3, Whi3, Ydj1, Far1, Whi5, Cln1, Cln2, Sic1and so on) and in ribosome biogenesis (such as Sfp1 and Sch9)^3^,^26^.

A well-accepted critical cell size control theory assumes two distinct, but interconnected, functions: sensing of actual cell size and setting of the critical cell size according to growth conditions^2^,^26^. Following this view, two mechanisms—possibly involving a (partially) different set of molecules—would be devoted to ‘weigh' the protein content of a cell and to set the actual critical cell size for each growth condition. Cln3, the most upstream cyclin regulating the cyclin-dependent kinase Cdk1, has been proposed as the sensing component^19^,^21^,^27^,^28^. The most recent model in this class holds that a yeast cell may titrate Cln3 molecules against the number of genomic binding sites for SBF, a heterodimeric transcription factor that regulates transcription of the G_1_/S regulon, encoding genes required to execute the G_1_/S transition^28^. Genome-wide screenings for mutants with altered cell size have suggested that ribosome biogenesis may set the cell size value characteristic for each growth rate^26^.

Single-cell analysis suggested a sizer plus timer model, in which Cln3 is involved in a noisy sizer control (whose complementary molecular component is unspecified), which sets a period within G_1_ (called *T*_1_) of considerable length in small daughter cells, but much shorter in parent cells^13^. *T*_1_ terminates with the export of Whi5 from the nucleus and the consequent transcriptional activation of the G_1_/S regulon. The following *T*_2_ period, which ends the G_1_ phase, has similar length in both parent and daughter cells and terminates with bud emergence and the onset of DNA replication^13^. The sensing/setting theory and the sizer plus timer models are reciprocally compatible and considerable effort has been dedicated to identify the counterpart of Cln3 in the sensing mechanism. A recently proposed model substantially departs from this common background to hold that the critical cell size is robustly set by the rate of linear growth during G_1_ and that the chaperone Ydj1 has an important role in regulating critical cell size^29^.

In this paper we focus on the core mechanism of cell size control in steady state growth, no consideration being given yet on how changes in nutrients or growth rate affect cell size. We present a novel and conceptually simple mathematical model of the G_1_/S network, centered on the phosphorylation of Whi5, an inhibitor of G_1_/S-specific transcription. Although our model includes all known molecular players of the G_1_/S transition, only few critical parameters (that affect Whi5 phosphorylation) determine its most important functions. Model predictions have been validated by analysing cell cycle entry and transcriptional activation in mutants expressing a Whi5 protein with constitutive (pseudo)phosphorylation. The model accounts for different data sets—including pheromone effects on cell cycle-related events—that were not considered in model design and parameter optimization, thereby providing a new unifying, comprehensive, molecular mechanism for the core critical cell size control and for the mitosis/mating switch.

## Results

### Outline of the model of the G_1_/S transition

**Figure 1:**
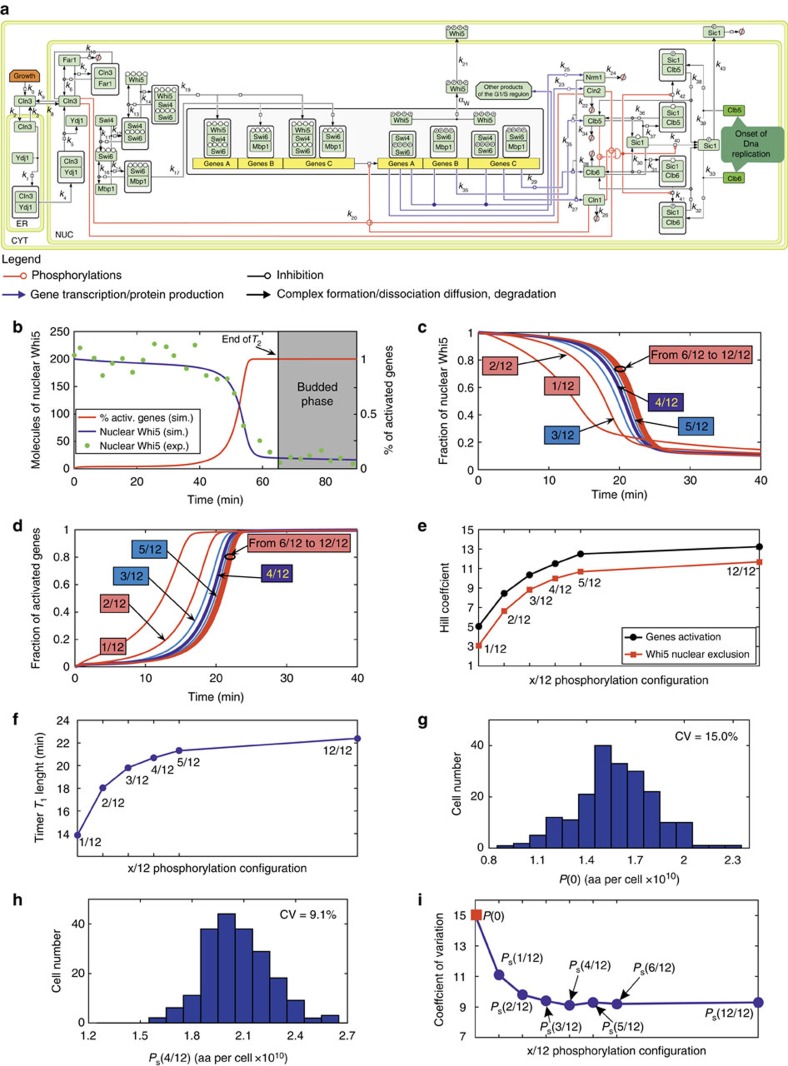
Multisite Whi5 phosphorylation controls the G_1_/S transition. (**a**) Map of the molecular processes regulating the G_1_/S transition. The box labelled ‘Growth' accounts for the growth equations (written in terms of the overall cell protein and ribosome amount) driving the molecular model. (**b**) Time course of the fraction of the activated genes of the G_1_/S regulon (red line) and the number of nuclear Whi5 molecules (blue line), obtained from the simulation of an extra-small daughter cell (*P*(0)=1.2*10^10^ aa, *R*(0)=1.6 × 10^5^ rib), fitting experimental Whi5 values from ref. 29. aa, amino acids. (**c**,**d**) Time course of the fraction of nuclear Whi5 (**c**) and of the activation of the G_1_/S regulon genes (**d**), obtained from the simulation of an average daughter cell according to different multisite phosphorylation schemes (12 total Whi5 phosphorylation sites, 1 to 12 functional sites). (**e**) Hill coefficients of the best-fitting Hill function for the curves reported in **c**,**d**. (**f**) *T*_1_ length for the curves reported in **c**,**d**. (**g**,**h**) Size (protein content) distribution of newborn cells (**g**) and of cells at the beginning of the budded phase according to the 4/12 Whi5 phosphorylation configuration. The simulations were carried out by sampling the initial *P*(0) from a log-normal distribution (average value 1.6 × 10^10^ aa, CV 15%) and fixing *R*(0)=*ρ* × *P*(0). In each simulation, all the parameters were allowed to vary (log-normal distribution) with 5% CV over the average values (see Supplementary Table 10 for details). (**i**) Diagram of the CVs for the distributions of different phosphorylation schemes, same rules to assign parameters and initial conditions as in **g**,**h** (distributions reported in Supplementary Fig. 6).

In our model (Fig. 1a; Supplementary Notes 1–10), synthesis of the upstream cyclin Cln3 links cell growth—modelled according to ref. 30—to molecular events promoting the G_1_/S transition. Cln3 synthesis is exquisitely sensitive to alterations in growth rate and ribosomal content^31^ and its average G_1_ concentration is higher in fast growing cells^19^,^21^,^31^. Conflicting evidences regarding the precise pattern of Cln3 protein accumulation during the G_1_ phase have been reported^32^,^33^,^34^,^35^. Our simulations show that the G_1_/S transition kinetics are quite insensitive to the specific pattern of Cln3 accumulation (Supplementary Figs 1–2), while they are dependent on average Cln3 level during G_1_ (*cln3*Δ and Cln3 overexpression cases). Therefore, we used the simpler hypothesis, that is, that Cln3 concentration is constant throughout the G_1_ phase. In early G_1_, the chaperone Ydj1 facilitates import of Cln3 in the nucleus where it binds the catalytic kinase subunit Cdk1 (ref. 27): the nuclear amount of Cln3–Cdk1 is therefore larger in larger cells and smaller in smaller cells. Coherent transcriptional activation of the G_1_/S regulon^36^ leads to synchronous expression of hundreds of gene products, orderly driving the G_1_/S transition that starts the pathway towards mitosis. It is driven by two heterodimeric transcription factors, SBF (composed by the co-activator Swi6 and the DNA binding protein Swi4) and MBF (composed by Swi6 and Mbp1 (ref. 37)) and inhibited by the transcriptional repressor Whi5 that regulates the G_1_/S transition in both mitotic^36^,^38^,^39^ and mating factor-treated cells^5^ through inhibition of several hundreds of SBF molecules.

Multisite phosphorylation of a regulatory protein is an effective molecular device able to produce a coherent response in the specific function controlled by the same protein, when the function involves hundreds of identical molecular players, as observed in the control of firing of DNA replication origins by phosphorylation of Sld2/Sld3 (refs 40, 41). As Cln3–Cdk1 accumulates, it phosphorylates Whi5 on 12 Cdk phosphoacceptor sites that are mostly occupied *in vivo* in asynchronously growing cells^42^. Whi5 is released from SBF when its four specific functional phosphosites are fully phosphorylated^42^,^43^. The other 8 sites—whose phosphorylation does not affect binding activity—act as decoys that compete with the functional sites for the available Cdk1 kinase activity. SBF–Whi5 dissociation may also derive from the full phosphorylation of four critical phosphosites^42^ of Swi6, a critical target of Cln3 (ref. 44). When Whi5 is released, transcription of the SBF-driven genes of the G_1_/S regulon may start^36^,^42^. Full phosphorylation of the four Swi6 phosphosites of MBF drives transcription of the MBF-regulated genes of the regulon.

The order of transcription of the genes encoding the more relevant cycle regulatory proteins—which may be fine-tuned in different experimental conditions—is: *CLN1*, *CLN2*, *CLB5*, *CLB6* and then *NRM1* (ref. 36). A positive-feedback loop that involves *CLN1* and *CLN2* transcription ultimately commits yeast cells to S phase^45^, while phosphorylated Whi5 is released from SBF and exported from the nucleus^5^,^13^,^29^,^38^. MBF is repressed by a product of the same regulon, Nrm1, which terminates the transcription of the regulon as cells progress towards S phase^46^.

The model includes cytoplasm, endoplasmic reticulum and nuclear sub-cellular compartments. Ordinary Differential Equations (ODEs) based on the mass–action law, modified when required to account for nonlinear behaviour (for example, for Cln3–Ydj1 diffusion into the nucleus, Cln3-dependent Far1 degradation, Sic1 phosphorylation and so on), describe synthesis, degradation, activity and sub-cellular localization of proteins and protein complexes. Coherent regulon activation depends on multisite phosphorylation of Whi5, SBF and MBF, catalysed by Cln–Cdk1 complexes. A discrete stochastic module calculates the probability distributions of the different phosphorylation states of the DNA-bound SBF (SBF–Whi5 complex) and MBF transcriptional activators and turns on G_1_/S transcription accordingly. The model computes percentage of the activated genes of the regulon, and dedicated ODEs compute synthesis of Cln1, Cln2, Clb5, Clb6 and Nrm1. G_1_ ends when 50% of Sic1—the inhibitor the Clb–Cdk1 complexes—has left the nucleus following phosphorylation by Cln1,2,3– and Clb5,6–Cdk1 complexes.

The mathematical model—constructed using a previously reported model^47^ as a stepping stone—is described in Supplementary Notes 1–10. Supplementary Tables 1–7 report initial conditions and input parameters. Supplementary Fig. 3 reports simulations of various molecular players in an average newborn daughter cell. In small elutriated cells, the simulated kinetics of translocation of Whi5 from the nucleus—an emergent property of the network—shows close agreement with experimental findings (Fig. 1b)^29^.

### Multisite Whi5 phosphorylation controls the G_1_/S transition

A convincing test of the usefulness of a mathematical model describing a complex biological process is given by its ability, expressed through simulation analysis, to quantitatively account for different experimental data sets and to offer new insight on how the distinctive functional features of a given biological process emerge from the interactions within its underlying molecular network.

Experimental evidence^42^,^43^ indicates that Whi5 release requires the phosphorylation by Cdk1 of a relatively small number (4) of functional phosphorylation sites within a larger pool (12) of phosphosites. We thus asked how the properties of cell cycle regulation change by altering the ratio between functional and decoy sites, fixing the total number of sites to 12. As the number of functional sites increases from 1 to 3, the coherence of Whi5 exclusion from the nucleus and of the activation of the G_1_/S regulon genes markedly increases (Fig. 1c,d), as indicated by their Hill coefficients, whose values more than double (Fig. 1e; Supplementary Fig. 4; Supplementary Movie 1). The length of the *T*_1_ period (Fig. 1f) substantially increases. The 3/12, 4/12 and 5/12 configurations had very similar parameters. A further increase in the number of functional phosphorylation sites (up to the 12/12 configuration) does not substantially modify the Hill coefficient nor further lengthens *T*_1_ (Fig. 1e,f). The fraction of activated genes for different phosphorylation configurations of Whi5 are reported in Supplementary Fig. 5, where the ratio of functional/total phosphorylation sites is kept fixed at 1/3.

These outputs are unaffected by the specific pattern of Cln3 accumulation (Supplementary Figs 1–2).

Another functional property of the G_1_/S transition, the significant reduction in cell size variability observed at the entrance into S phase^13^, appears affected by Whi5 multisite phosphorylation. Simulation of the G_1_/S transition for a cohort of newborn daughter cells with the *P*(0) size distribution reported in Fig. 1g was performed with a varying number of functional Whi5 sites to obtain the distribution of the critical cell size *P*_s_ (Fig. 1h, case 4/12). Starting with a coefficient of variation (CV) of 15% at *P*(0), *P*_s_ variability decreases by increasing the number of functional sites of Whi5 from 1 to 4 and remains constant thereafter (see Fig. 1i and Supplementary Fig. 6). These findings, together with previous genetic^42^ and structural evidences^43^, indicate that the 4/12 configuration for Whi5 phosphorylation is likely to be the wild-type one.

### Choice of cell fate determined by mating factor

**Figure 2:**
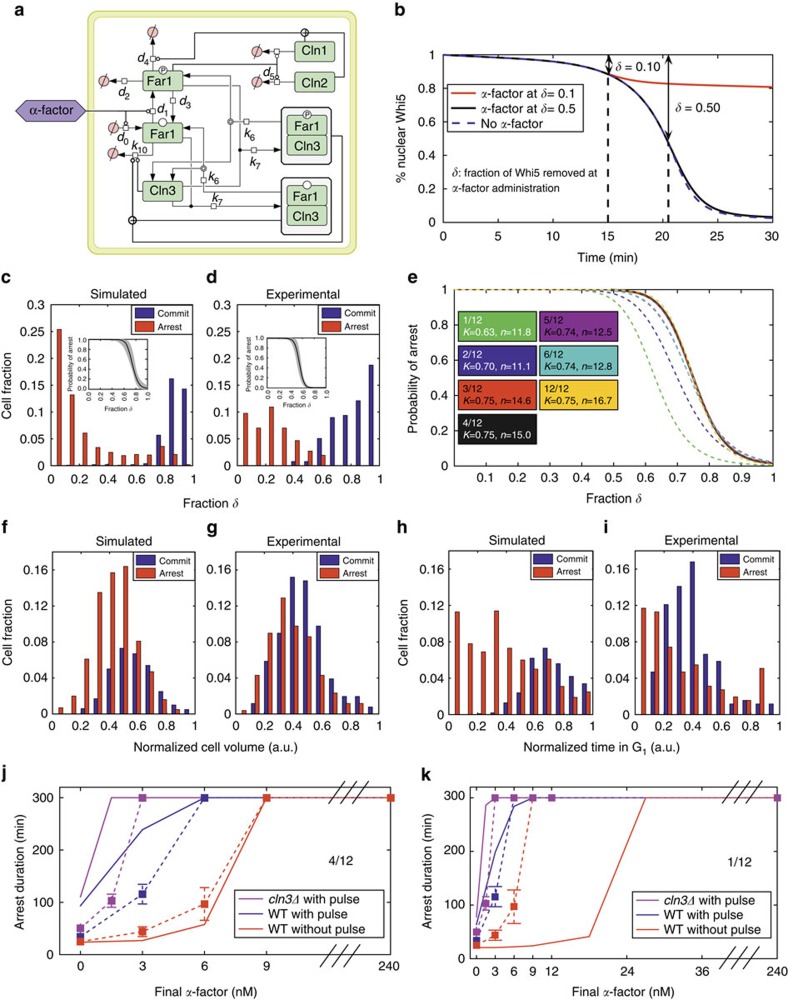
Choice of cell fate determined by mating factor. (**a**) Scheme of the molecular processes involved in α-factor-induced cell cycle arrest. (**b**) Time course of Whi5 localization after switching the parameters to the α-factor set at a different fraction of nuclear Whi5, obtained by the simulation of an average daughter cell. (**c**,**d**,**f**–**i**) Histograms for fractions of cells arrested in G_1_ phase (in red) or committed to S phase (in blue). (**c**,**f**,**h**) refer to 1,000 simulated cells (4/12 Whi5 phosphorylation configuration, see Supplementary Table 10 for the details): distributions are drawn with respect to *δ*, the fraction of Whi5 removed from the nucleus at the time of α-factor administration (**c**), with respect to the normalized cell size (**f**) and with respect to the normalized time in G_1_ (**h**). The histograms in **c**,**f**,**h** achieved in simulations are compared with the histograms achieved from experimental data (**d**,**g**,**i**, respectively, redrawn from ref. 5). The insets in **c**,**d** show the probability of arrest in G_1_ phase versus fraction *δ*, with the grey region indicating the 95% confidence intervals based on 10,000 bootstrapping iterations. (**e**) Reports the probability of arrest curve for different Whi5 phosphorylation schemes: the legends report the median point (*K*) and the Hill coefficient (*n*) of the Hill functions best-fitting the fractions of arrested cells as coming from the histograms. (**j**,**k**) Duration of arrest in G_1_ phase versus the continuous baseline administration of α-factor according to the 4/12 (**j**) and 1/12 (**k**) phosphorylation configurations. Simulated data (continuous line) are compared with experimental data (markers, reference to ref. 48). A high concentration, 30 min long α-factor pulse was given when a very low fraction of Whi5 was removed from the nucleus (<2%). The maximum waiting time was set to 300 min according to ref. 48. See Supplementary Table 9 for the implementation details.

G_1_ arrest is a major pre-requisite for conjugation between haploid yeast cells of opposite mating types. Far1, the Cln–Cdk1 inhibitor presented in the model in Fig. 1a, plays a key role in this arrest. To describe cell mating, we added a module (Fig. 2a) that recapitulates the α-factor-dependent cell cycle-related events by abruptly changing the relevant parameters when the mating factor is added, following a recently described mathematical formalism^5^,^48^ (see Supplementary Note 11 and Supplementary Table 8 for parameter definitions).

The mating/mitosis switch is very sensitive to the level of nuclear Whi5 at the time of α-factor addition: when the fraction of removed Whi5 (*δ*) is low (around 10–30%), there is a very high probability of cell cycle arrest, while when it is high (over 50%), the probability of cell cycle arrest drops close to zero^5^. Simulation with the standard (4/12) configuration for Whi5 phosphorylation and standard values for parameters neatly predicts the experimental behaviour (Fig. 2b), since the addition of α-factor when *δ*=0.1 yielded cell cycle arrest, whereas pheromone addition when *δ*=0.5 allows the cell cycle to proceed unperturbed towards mitosis.

Populations of daughter cells were exposed to virtual α-factor addition at different times after birth, their G_1_/S transition was simulated with the extended model (4/12 Whi5 configuration) and their cell fate was recorded as cell cycle arrest or entrance into S phase. The pattern of arrested cells as a function of parameter *δ* is a good predictor of α-factor-induced arrest, with little overlap between the two subpopulations of arrested and committed cells as shown for experimental data (Fig. 2c,d). The insets report the probability of arrest, computed from the distribution of arrested cells, the shaded region indicating the 95% confidence intervals based on 10,000 bootstrapping iterations^5^. Our simulations refer to daughter cells only: almost 40% of them result committed, in good agreement with the 40% experimental value^5^, but lower than the value observed in the experimental mixed (daughter/parent) population (Fig. 2d right, see also Fig. 2f in ref. 5). The pattern of arrest for different Whi5 phosphorylation configurations was very similar, with a mild increasing trend for the Hill coefficient and the median point when the number of functional sites rises from 1/12 to 4/12. (Fig. 2e; Supplementary Fig. 7). Simulation results further indicate that the parameter *δ* is a better predictor of α-factor-induced arrest than the normalized volume or the time spent in G_1_, in agreement with experimental findings^5^ (Fig. 2c,d,f-i).

The mating response presents hysteresis: cells pre-treated with a brief pulse of saturating α-factor arrest cell cycle when treated with a suboptimal α-factor concentration, which alone is normally ineffective^48^. Hysteresis was reproduced by our model (Fig. 2j, 4/12 configuration). Consistently with experimental results, *CLN3* deletion reinforced hysteresis, in keeping with the notion that Cln3–Cdk1 is the major kinase involved in the recovery from α-factor treatment and thus a privileged target of Far1 inhibitory action. Hysteresis was also detected in simulations performed with the 1/12 configuration for Whi5 phosphorylation, but full arrest required a higher concentration of virtual α-factor (note different abscissa scale in Fig, 2j,k. See also Supplementary Fig. 8). In summary, the extended model captures the major distinctive features of α-factor-induced cell cycle arrest.

### G_1_ duration in daughters born with different size

Newborn daughter cells show large size variability. Smaller cells delay entry into S phase, suggesting a deterministic requirement for a critical cell size in the execution of the G_1_/S transition^13^. Statistical analysis suggested that an efficient sizer operates in smaller daughter cells, whereas in larger ones a timer appears to be active, size-independent molecular noise providing the largest quantitative contribution to G_1_ variability^13^.

**Figure 3:**
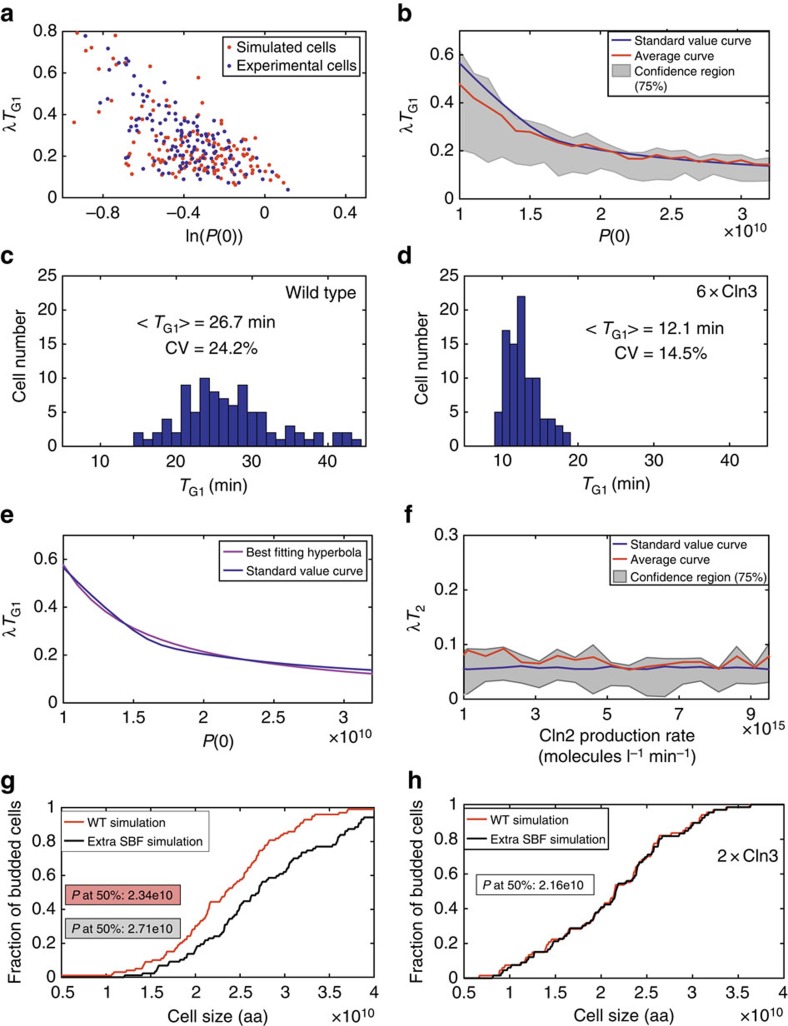
G_1_ duration in daughters born with different size. (**a**) Correlation between *λT*_G1_ and ln(*P*(0)), with *P*(0) normalized to the average size at budding of the cell population. Experimental values (blue) from ref. 13, simulated data (red) refer to average and extra-small populations (see Supplementary Table 10 for the implementation details). A CV value of 20% was chosen for the initial protein distribution, given that the experimental sample to be compared with simulation results includes unusually small cells^13^. (**b**) Correlation between *λT*_G1_ and *P*(0). Twenty three cells differing only in their initial protein content were simulated (blue line). The red line refers to the average results of 50 separate cells in each initial condition, parameters allowed to vary as described in Supplementary Table 10. The 75% quantile region is in grey. (**c**,**d**) G_1_ phase length heterogeneity for wild-type cells (**c**) and cells overexpressing Cln3 (6 × over wild-type, **d**). (**e**) Best-fitting hyperbola for the standard value curve of *λT*_G1_ (blue line, **b**) versus the initial protein content *P*(0). (**f**) Correlation between *λT*_2_ and the Cln2 production rate. Twenty three cells differing only in their Cln2 production rate were simulated (blue line). The red line refers to the average results of 50 separate cells in each initial condition, with parameters that were allowed to vary as described in Supplementary Table 10. The 75% quantile region is in grey. (**g**,**h**) Fraction of budded cells *versus* cell size. (**g**) compares budding kinetics for wild-type cells and cells including extra SBF-binding sites; (**h**) compares the same two curves for cells overexpressing Cln3. See Supplementary Table 10 for implementation details.

To account for these data, we simulated a population of daughter cells (Fig. 3a, red dots) with different birth size *P*(0) and found that it superimposes quite well with experimental findings (blue dots) in a plot of *λT*_G1_ (where *λ* is the rate of exponential growth) versus ln[*P*(0)]^13^. The blue line in Fig. 3b (standard value curve) plots the *λT*_G1_ value obtained from simulations of about 20 single cells sharing the same model parameters, and having a different *P*(0), selected at even intervals in the same range covered by the population of newborn cells shown in Fig. 3a. The red line corresponds to the average value of 50 simulations for each *P*(0) value, with parameters allowed to vary according to a log-normal distribution, with a 12% coefficient of variation. The 75% quantile region (grey area), indicates that variability is larger for smaller cells and that larger cells converge to a constant *λT*_G1_ value. The large variability in *λT*_G1_ of smaller cells is not directly due to cell size, but is strongly related to the low amount of Cln3 present. In fact a cell population with the same initial cell size distribution as wild-type cells and a 6-fold higher Cln3 level - that allows more efficient phosphorylation of Whi5—shows a decrease in G_1_ length and reduced variability (Fig. 3c,d).

The standard value curve was very well fitted by a hyperbolic curve (see Supplementary Notes 14–15; red line in Fig. 3e) rather than by the previously proposed broken-line plot^13^,^29^. The equation of the best-fitting hyperbola (Supplementary Note 15, eq.(C.5.1.1)) allows to estimate *λT*_G1_ from any *P*(0) value, providing an input–output relationship (resembling numerical results achievable by simulations from the model in Fig. 1a), which cannot be derived through an analytical approach.

In our model, the *T*_2_ period, which begins when 50% of Whi5 has been exported from the nucleus, terminates when 50% of nuclear Sic1 is exported from the nucleus. We analysed the impact of *CLN2* dosage—simulated by the appropriate alteration of SBF/MBF dependent synthesis—on *T*_2_ length. Using the same approach utilized in Fig. 3b, we show that *T*_2_ length and its variability are insensitive to *CLN2* dosage (Fig. 3f). Di Talia *et al*.^13^ experimentally decomposed the overall noise in G_1_ length into a size-dependent and a size-independent part. Table 1 compares the simulated noise (see Supplementary Note 16) with the experimental one. The overall and decomposed values for G_1_ noise compared quite well for wild-type cells and Cln3-overexpressing cells, with the general tendency of the simulated cells to be less noisy than their real counterparts. Our model rationalizes the observed size variability and newly links it quantitatively to the variability of the G_1_ phase duration.

The observation that increasing the number of SBF-binding sites in a cell delays budding and increases the critical cell size, as if a genomic binding site participates to the sensing mechanism, was considered connected to the relation between critical cell size and ploidy^28^. Simulation of our model in the presence of an increased number of SBF-binding sites (and hence of bound Whi5) reproduces the experimentally observed increase in cell size (red and black lines, Fig. 3g). Consistently with the notion that Cln3–Cdk1 activity is the major limiting factor in the G_1_/S transition, an increase in Cln3 quenched the effects of the increased availability of SBF-binding sites (compare the red and black lines in Fig. 3h), as experimentally reported^28^. Thus, multisite phosphorylation of SBF-bound Whi5 fully explains the findings that were interpreted to indicate titration of SBF-binding sites by Cln3 (ref. 28).

### Linear growth rate and critical cell size

Ferrezuelo *et al*.^29^ recently proposed a theory of critical cell size control that drastically departs from the sensing and setting theory^2^,^26^. They noticed that experimentally determined values of *V*_s_ (the cell volume at the end of *T*_1_, that is, when 50% of Whi5 has been translocated out of the nucleus) are proportional to the linear growth rate in *T*_1_, called *α*. On these basis they proposed that ‘the critical cell size is set at a single-cell level by linear growth rate'^29^. (see also Supplementary Fig. 9). In the following, we test whether the experimental findings reported in ref. 29 could find an alternative interpretation in our model.

**Figure 4:**
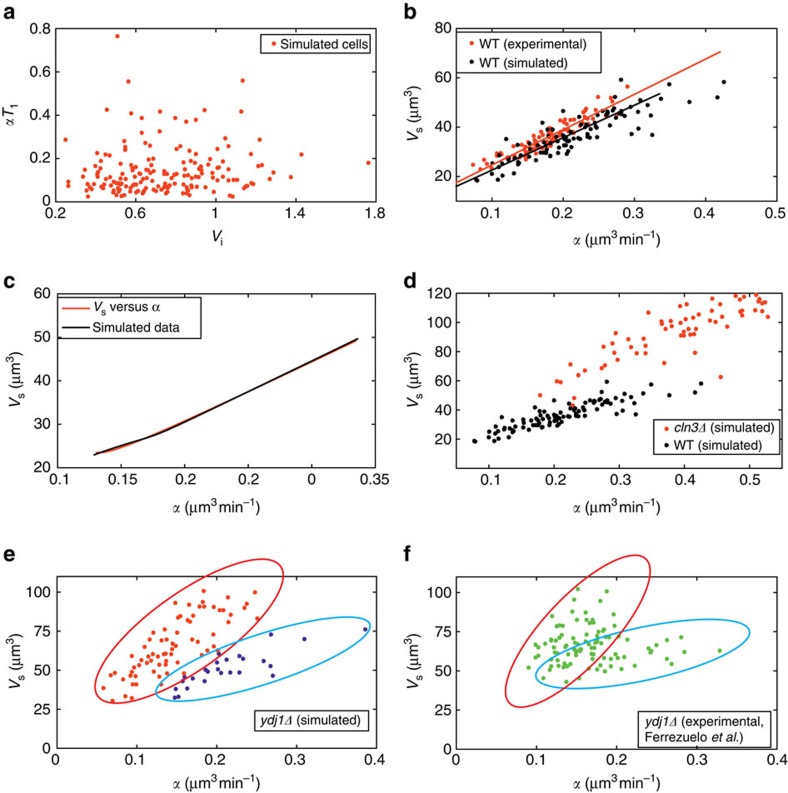
Linear growth rate and critical cell size. (**a**) Correlation between the product *αT*_1_ and the initial volume *V*_i_, 100 simulated cells. (**b**) Correlation between the volume at the end of *T*_1_ (*V*_s_) and the linear volume growth rate (*α*). Each black point represents one cell from a population of 100 daughter cells. All parameters were allowed to vary (log-normal distribution) with a 12% CV over their average values (see Supplementary Table 10 for details). Red points are experimental data from ref. 29. (**c**) Volume at the end of *T*_1_ versus linear growth rate (*α*). The black line corresponds to the simulated data. The red line corresponds to the relationship of *V*_s_ versus *α*, which was determined by exploiting the best-fitting hyperbola of Fig. 3e between *λT*_G1_ and *P*(0). (**d**) Correlation between the volume at the end of *T*_1_ and the linear volume growth rate (*α*) for mutant *cln3* (red) and WT cells (black circles, same data as in **b**). (**e**,**f**) Correlation between the volume at the end of *T*_1_ and the linear volume growth rate (*α*) for mutant *ydj1* (simulated data in **e**, experimental data in **f**). In **e**, clusters refer to cells simulated according to growth parameters providing an exponential growth rate of 0.0031, min^−1^ (red cluster) or 0.0063, min^−1^ (blue cluster). In **f**, the red cluster refers to cells of the experimental sample reported in ref. 29 that fall within the grid of Supplementary Fig. 10C. The blue cluster comprises the remaining cells.

Hence we considered a standard daughter cell population similar to that shown in Fig. 3a. Figure 4a reports the plot of computed *αT*_1_ versus *V*_i_ values, which have been used to construct the corresponding *V*_s_ (volume at the end of timer *T*_1_, proportional to critical cell size) versus *α* plot (black dots) in Fig. 4b. Simulated data of cells growing according to an exponential growth kinetics well approximate experimental data taken from ref. 29. The linear relationship between *V*_s_ and *α* can also be obtained by analytical approaches (Fig. 4c; see also Supplementary Notes 17–18 and Supplementary Fig. 10), starting from the best-fitting hyperbola shown in Fig. 3e. In conclusion, the observed linear relation between *V*_s_ and *α* does not have as unique possible interpretation the one proposed in ref. 29 and hence it is not necessary to hold that it is the linear growth rate that determines the critical cell size.

We next simulated the behaviour of two mutants with deletions in *CLN3* and *YDJ1* (Fig. 4d,e, respectively). Inactivation of Cln3 yields larger cells. The *V*_s_ versus *α* plot obtained by a simulated *cln3Δ* population is in good agreement with the experimental one^29^ (compare Fig. 4d to experimental data in Fig. 4d of ref. 29). Experimental deletion of the *YDJ1* gene results in a very disperse *V*_s_ versus *α* plot that suggests the existence of two subpopulations with different growth rates contained within the red and blue ellipses in Fig. 4f. Gene-dosage experiments are often accompanied by changes in growth rate and large transcriptional reprogramming^49^,^50^,^51^. Ydj1 interacts either physically or genetically with almost 500 unique genes or gene products and deletion of its encoding gene induces slow growth and reduced fitness and lifespan (www.yeastgenome.org; Supplementary Fig. 11). Accordingly, simulation of two subpopulations differing in growth rate is required to obtain a good fitting to experimental data (Fig. 4e compared with Fig. 4f).

### Parameter-sensitivity analysis

The quantifiable outputs of a multiparameter model that aims—as our model does—to describe complex biochemical networks may not contain enough information to ensure assignment of a univocal value to each parameter in the parameter space. Application of a formal Bayesian approach^52^ is hindered (i) by the difficulties in achieving any *a priori* characterization of the probability density of parameters like time constants, kinetic coefficients and thresholds and (ii) by the observation that systems biology models share the sloppiness property^53^,^54^, with the term ‘sloppiness' referring to ‘the highly anisotropic structure of parameter space, wherein the behaviour of models is highly sensitive to variation along a few stiff directions (combinations of model parameters) and more or less insensitive to variation along a large number of ‘sloppy' directions'^55^.

**Figure 5:**
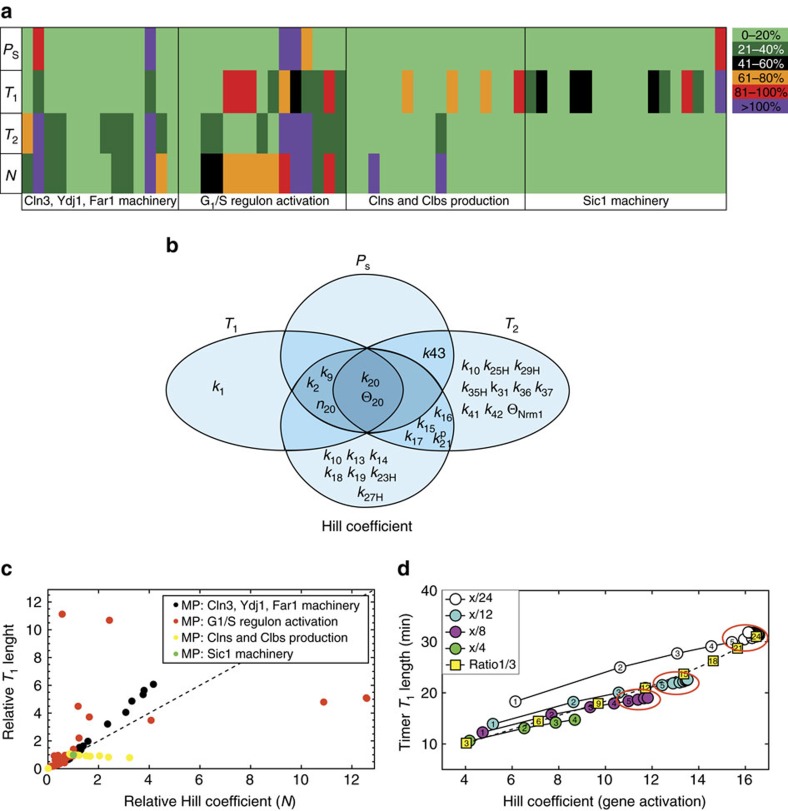
Parameter-sensitivity analysis. (**a**) Heat map describing the effect of parameter variation on the outputs of the G_1_/S transition model. (**b**) Venn chart summarizing the parameters whose variation affects one or more outputs of the G_1_/S transition model. (**c**,**d**) Correlation analysis of the changes in *T*_1_ length as a function of the changes in the Hill coefficient *N*. Each point in **c** refers to a single-cell simulation in which one model parameter—colour-coded by functional class—was altered. The *x* and *y* axes are the relative output values, with the value of the standard parameter set to 1. The black dotted line represents the expected output for a 1-to-1 correlation. Each point in **d** refers to a single-cell simulation in which different Whi5 phosphorylation settings were considered. Coloured circles refer to simulation sets with a fixed total number of phosphorylation sites and a variable number of functional sites (indicated within the circle itself). Yellow boxes refer to simulation with fixed (1/3) ratio of functional/total phosphorylation sites. The total number of sites is indicated within the square.

Using sensitivity analysis, we assessed how parameter variation affects four major outputs of the model: the length of Timer *T*_1_ and *T*_2_, the critical size *P*_s_ and the Hill coefficient (*N*) of the best Hill function fitting the G_1_/S regulon activation curve. Alteration of only two parameters (*k*_20_ and *θ*_20_, which affect Whi5 phosphorylation) impacts on all four tested outputs (Fig. 5a,b). The Timer lengths and the sharpness of the G_1_/S regulon activation curve are nontrivially sensitive to a small set of parameters, in keeping with the sloppiness hypothesis (see also Supplementary Figs 12–15). Only the length of *T*_1_, but not of *P*_s_ and *T*_2_, shows some correlation with changes in the Hill coefficient *N* (Fig. 5c; Supplementary Fig. 16), notably so for parameters related to the Cln3, Ydj1, Far1 machinery and for some parameters related to G_1_/S regulon activation (Fig. 5c, black and red points, respectively).

We then analysed the impact of the functional (4) to decoy (8) configuration of Whi5 on the coherence of the G_1_/S transcription (quantified by *N*, the Hill coefficient for transcriptional activation) and on the timing delay, monitored as duration of *T*_1_ period. The connection between *N* and *T*_1_ is shown in Fig. 5d. Increasing the total number of sites (from 4 to 24) with a variable number of functional sites, there is a sizable increase of both *N* and *T*_1_, till functional sites reach 4. Above this value, saturation is obtained. Increasing the total number of sites from 3 to 24 and keeping a constant ratio (1/3 as in 4/12 Whi5) between functional and total sites, the increase in total sites correlates with an increase of both *T*_1_ length and Hill coefficient value.

### The Whi5^4E^ mutant anticipates entrance into S phase

To be fully accepted, a new model is required both to account for large sets of existing data and to make predictions qualitatively distinct from its predecessors, which need to be validated by new, independent sets of experimental findings.

**Figure 6:**
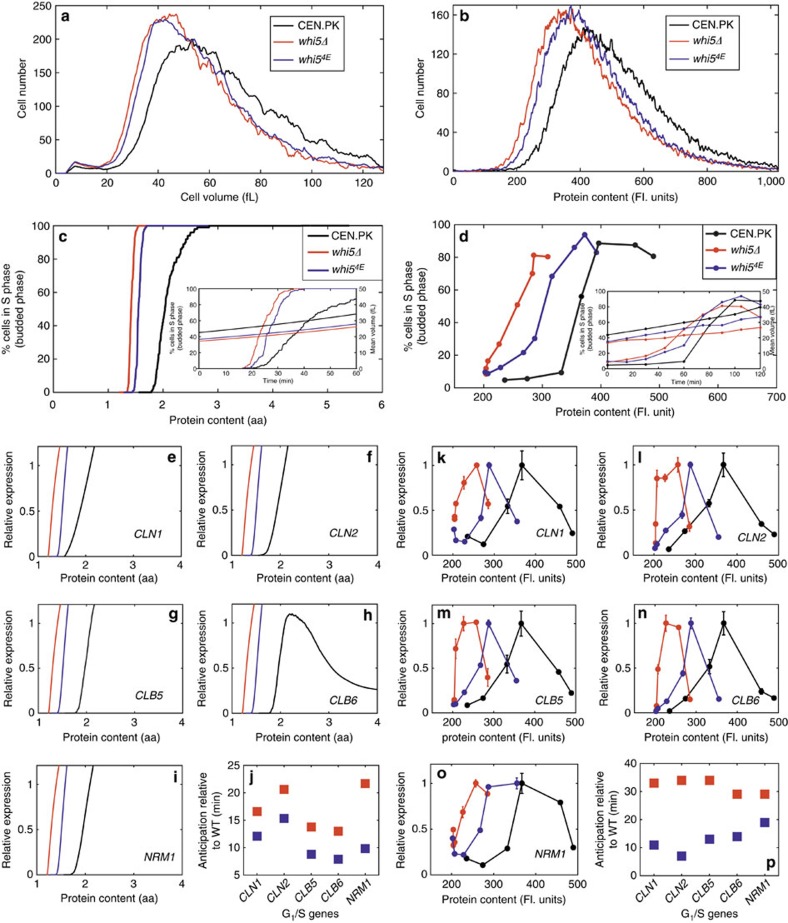
Whi5^4E^ mutant anticipates entrance into S phase. (**a**,**b**) Cell volume and protein distributions of wild type, *whi5Δ* and *whi5*^*4E*^ cells exponentially growing in glucose-supplemented medium. Data representative from three independent experiments are shown. Predicted (**c**) and experimental (**d**) kinetics of cell cycle entry for wild type (black), *whi5Δ* (red) and *whi5*^*4E*^ (blue) cells. Predicted (**e**-**j**) and experimental (**k**-**p**) transcriptional activation of selected genes of the G_1_/S regulon in wild type (black), *whi5Δ* (red) and *whi5*^*4E*^ (blue) cells expressed as a function of cell size. Experimental data in **k**–**p** represent means±s.d.'s (*n*=3). Cell cycle entry and transcriptional activation are shown as a function of cell size; inserts in **c**,**d** show cell cycle entry expressed as a function of time (time 0 being reinoculation in fresh medium after elutriation). (**j**,**p**) summarize the time for which each gene anticipates the reaching of 50% expression in *whi5Δ* (red) and *whi5*^*4E*^ (blue) mutants in comparison with wild type.

Glutamate may mimic phosphoserine or phosphothreonine^56^. A Whi5^4E^ protein in which the four functional phosphosites have been mutated to glutamate should dissociate from SBF nearly as well as Whi5 in which the functional phosphosites have been phosphorylated by Cdk1. Since (pseudo)phosphorylation is constitutive and unregulated, yeast cells harbouring the mutated Whi5^4E^ protein should exhibit a small (Whi) phenotype similar to the *whi5Δ* mutant. Our model further predicts that Whi5^4E^ G_1_ cells would anticipate the activation of the G_1_/S regulon, entering the S (budded) phase at a much smaller size than their wild-type counterparts (Fig. 6c,e–j). Since our model does not include events following the G_1_/S transition that down-regulate the G_1_/S regulon and lead to cell division, the fraction of budded cells remains high and transcription of genes of the G_1_/S phase steadily increases, except for *CLB6*, whose expression is downregulated by Nrm1.

We constructed a strain expressing the mutated Whi5^4E^ protein. The transcript levels of *WHI5*^*4E*^ and *WHI5* were undistinguishable (Supplementary Fig. 17). Cell volume and cell protein distributions (Fig. 6a,b) confirmed that the *whi5*^*4E*^ strain maintains a Whi phenotype, being much closer to the *whi5Δ* mutant than to wild type. When reinoculated in fresh glucose medium *whi5*^*4E*^ small G_1_ cells (isolated by elutriation) showed an anticipated entry in the budded, S phase (Fig. 6d) and an early activation of G_1_/S regulon (Fig. 6k–p) relative to wild-type cells. In keeping with model predictions, both cell cycle entry and gene expression kinetics of the *whi5*^*4E*^ mutant are intermediate between those of the wild type and of the *whi5Δ* mutant, being more similar to the latter.

Taken together these results validate the central notion of our model: dissociation of Whi5 from SBF is promoted by phosphorylation of its functional phosphoacceptor sites.

## Discussion

The cell-fate decision given by the mitosis/mating switch and the connected critical cell size control—the gate-keeper for the commitment of G_1_ cells to cell division—has been for decades one of the most studied examples of complex biological functions. Genetic and biochemical analyses have identified many of the involved players without being able to propose a fully satisfactory, comprehensive molecular model. By utilizing a systems biology approach which integrates mathematical modelling and computational analysis with experimental validation, this paper proposes a precise, quantitative, predictive and unifying mechanism of the molecular events involved in this cell fate decision, accounting also for a large set of experimental data, which have been used to propose different and even conflicting conceptual models for critical cell size control^13^,^26^,^29^.

The novelty proposed by our paper is given by the multisite phosphorylation of Whi5 by Cln3–Cdk1 (first) and Cln1,2–Cdk1 (later). Our model assumes that Cln3 concentration is constant during G_1_. Our simulations show that the specific pattern of Cln3 accumulation has negligible effects on the outputs of the model (Supplementary Figs 1 and 2), which are instead affected by Cln3 average content. Nuclear Cln3 import is favored by the Yjd1 chaperone^27^,^29^, the amount of nuclear Cln3 being larger in big cells than in small ones. We show that Cln3–Cdk1 is rate limiting for Whi5 phosphorylation, except than in very large cells. Cdk phosphoacceptor sites inside Whi5 include both decoy and functional sites^42^,^43^. Decoy sites fruitlessly engage Cln3–Cdk1 complexes, delaying phosphorylation of the four functional sites. Full phosphorylation of Whi5 (or of the Swi6 subunit of SBF) is required to release Whi5 from SBF. This mechanism originates synchronous dissociation of Whi5 from SBF at the hundreds of different promoters of the G_1_ regulon present in each cell, ensuring the coherence of the G_1_/S regulon transcription, essential for an ordered cell cycle progression and fully accounting for the observed role of Whi5 as the integrator of the signalling generated by Cln3 (ref. 57).

As an experimental validation, we report that Whi5^4E^—a mutant protein mimicking constitutive phosphorylation of the four functional residues—causes anticipated activation of the G_1_/S regulon and S phase entrance at a reduced size, close to that of the *whi5Δ* strain. Pseudophosphorylation of the functional residues likely induces a change in folding of Whi5, an intrinsically disordered protein^43^, that would act as a regulatory switch^58^, by reducing the binding affinity of Whi5 to SBF.

As anticipated in the Introduction, our paper does not cover the modulation of critical cell size by nutrients, but we may offer a working hypothesis, elaborated following the idea^2^,^26^ that the mechanism that sets the critical cell size in response to nutrients adds molecular components to the basic mechanism (the dynamic interaction of Cln–Cdk1 and Whi5) by which cells gauge their size. Ribosome biosynthesis, regulated by cAMP/PKA and Tor pathways (sensors of nutrient availability^22^,^26^,^59^,^60^), has been identified as the primary actor in the setting of the critical sell size by nutrients^26^,^61^,^62^. Mutations in ribosome biogenesis strongly affect Whi5 nuclear retention^63^. Whi5 has many phosphorylation sites, specific not only for Cdk1, but also for other pathways^43^, and may therefore integrate contrasting inputs, as reported in osmostress that may affect Whi5 binding activity^64^. On the other hand, the Cln3 level is very sensitive to changes in ribosome activity^31^.Our model (in which the exponential growth rate is dependent on the rate of protein synthesis) predicts that critical cell size becomes larger as a cell increases its rate of protein synthesis (Supplementary Fig. 18). Thus, nutrients, through signalling, may affect ribosome biogenesis that, directly or indirectly, affects the Cln3/Whi5 interplay. The clarification of the proposed mechanism of critical cell size setting by nutrients may be brought on by new, quantitative, integrated, dynamic investigations performed taking into account previously collected and newly generated evidence.

## Methods

### Yeast experiments

*Whi5* mutants were generated in CEN.PK2-1C genetic background (*MATa ura3-52 trp1-289 leu2-3,112 his3Δ 1 MAL2-8*^*C*^ *SUC2*, www.euroscarf.de (ref. 65)). Recombinant DNA manipulation and yeast transformation were performed according to standard protocols.

A DNA fragment encoding the Whi5^4E^ CDK mutant was synthesized *de novo* by Eurofins (www.eurofins.com) and subcloned into the YIplac211 integrative plasmid^66^ under the *WHI5-545*_*pr*_ native promoter^42^, yielding the construct YIplac211-*545*_*pr*_*-WHI5*^*4E*^. Single-copy genomic integration of the construct at the *URA3* locus was verified by quantitative PCR.

Yeast cultures were grown in synthetic complete minimal medium, containing 0.67% (w/v) yeast nitrogen base (YNB), appropriate quantities of the ‘drop-out' amino acid–nucleotide mixture (Formedium) and supplemented with 2% (w/v) glucose.

Growth of cultures was monitored as increase in cell number using a Coulter Counter model Z2 (Coulter Electronics, Inc.). The fraction of budded cells was scored by direct microscopic observation on at least 400 cells, fixed in 3.6% formaldehyde and mildly sonicated. Cell size analysis was performed using a Coulter Z2 Particle Cell Analyzer (Beckman-Coulter).

For synchronization studies, yeast cells were grown till late exponential phase in 2 l of csm/YNB medium supplemented with 2% raffinose^38^. Small G_1_ phase cells were isolated by centrifugal elutriation using a 40-ml chamber elutriator (Beckman Coulter) and released into fresh 2% glucose medium at 5 × 10^6^ cells per ml. Samples were collected at appropriate intervals for determination of budding index, cell volume, DNA and protein content by flow cytometry and for quantitative PCR with reverse transcription (qRT–PCR).

At least 2 × 10^7^ cells were collected and fixed in 70% ethanol before the cytofluorimetric analysis. For RNA staining, cells were washed once with cold PBS (3.3 mM NaH_2_PO_4_, 6.7 mM Na_2_HPO_4_, 127 mM NaCl. 0.2 mM EDTA, pH 7.2), resuspended in 1 ml of Propidium Iodide staining solution (0.046 mM propidium iodide in 0.05 M Tris-HCl, pH=7.7; 15 mM MgCl_2_) and incubated for 30 min on ice/dark. To obtain protein distribution, cells were stained with fluorescein isothyocyanate solution (50 μg ml^−1^ FITC in 0.5M NaHCO_3_) for 30 min on ice/dark and washed three times in PBS before the analysis. For DNA staining, cells were washed once in PBS, resuspended in 1 ml of PBS containing 1 mg ml^−1^ RNAse and incubated at least 12 h at 37 °C. Cells were then washed once with PBS, resuspended in 1 ml of Propidium Iodide solution and incubated for 30 min on ice/dark. All cell suspensions were sonicated 30 s before the analysis, which was performed with a FACSCalibur (Becton Dickinson) instrument equipped with a 488 nm Ion-Argon laser. Sample flow rate during analysis did not exceed 1,000 cells per s. Typically, 50,000 cells were analysed for each sample.

For RNA extraction, ∼2 × 10^8^ cells were collected by filtration, washed with 5% Trichloroacetic acid solution and rapidly freezed at −80 °C. Cells were resuspended in 500 μl of LETS buffer (200 mM LiCl, 20 mM EDTA, 20 mM Tris pH=7.4, 0.5% SDS), plus 300 μl of phenol:chloroform:isoamyl-alcohol solution (PCI) and lysed by glass beads. Two PCI extractions were performed on the recovered aqueous phase. RNA was subsequently isolated by ethanol precipitation in the of presence LiCl. Five hundred micrograms of RNA were treated with 9 units of DNase I (RNase-free, Qiagen) for 1 h at 37 °C. Total RNA was then purified with the RNeasy kit (QIagen) and 0.5 μg were used for cDNA synthesis using the iScript cDNA Synthesis Kit (BIO-RAD) according to the manufacturer's instructions. qRT–PCR reactions (final volume 15 μl) were performed in 48-well reaction microplates suitable for the MiniOpticon detection system (BIO-RAD) using the SsoFast EvaGreen Supermix (BIO-RAD). Primers used for the assay were designed with the Beacon Designer software (PREMIER Biosoft).

*CLN1*: forward 5′-TGACAGTGCCATAAGCGTAA-3′; reverse 5′-GTTGTGGTATTTCAGCGGATG-3′

*CLN2*: forward 5′-TCTCAAAGCCACACTCCAAT-3′; reverse 5′-ACGGTGCTACCACATATACTG-3′

*CLB5*: forward 5′-ATGAAAATGAGAGGCAGTTGTG-3′; reverse 5′-CTGTTAAAGCCCTTCTTGGTTT-3′

*CLB6:* forward 5′-CTTGACACCTCACTCTACGAAT-3′; reverse 5′-AAATGGGTGGAATCTCTTTGC-3′

*NMR1:* forward 5′-GAGCACTACCGCAGATATGA-3′; reverse 5′-GCTTGTAGGTGTTTCTTGTCTC-3′

*WHI5:* forward 5′-GTGTATATGACCATAACGACGAC-3′; reverse 5′-TGGCAACAAAGGCATACTAAC-3′

Data obtained were analysed with the CFX Manager software (BIO-RAD) and normalized by including *TAF10* and *UBC6* as reference genes in each plate. At least two biological replicates were analysed in triplicate for each gene^49^.

### Model construction

The proposed mathematical model links a basic set of growth equations to the ones related to the molecular player dynamics promoting the G_1_/S transition. The growth equations are taken from ref. 30, and deal with the ribosome and protein content. The molecular model equations take into account the following classes of events:

- Regulation of Cln3–Cdk1 activity, that is, Cln3 synthesis (in the cytoplasm), its subsequent retention in the endoplasmic reticulum and its transport in the nucleus by the Ydj1 chaperone as well as Cln3 inhibition by Far1.
- Transcriptional activation of the G_1_/S regulon, first by Cln3–Cdk1 kinase activity, which includes a computationally effective description of regulon activation—including Cln1,2-mediated positive feedback—and multisite phosphorylation of Whi5.
- Regulation of Clb5,6–Cdk1 activity—that triggers entrance into S phase—by binding of Sic1 whose nuclear level and inhibitory activity are regulated by Cln1,2–Cdk1 AND by Clb5,6–Cdk1 phosphorylation.

The link between the growth equations and the molecular model is given by the synthesis of the upstream cyclin, Cln3, since its total amount is constrained to be proportional to the overall protein content.

The model—which takes into account cytoplasm, endoplasmic reticulum and nucleus sub-cellular compartments—is a hybrid deterministic–stochastic model. The amount and sub-cellular localization of the molecular players involved in the activation of the regulon is modelled by a set of ODE. Regulon activation depends on a correct sequence of multisite phosphorylation steps—catalysed by Cln1,2,3–Cdk1 complexes. A stochastic model that calculates probability distributions of the different phosphorylation states describes the phosphorylation states of DNA-bound SBF and MBF transcriptional activators, as well as of the transcriptional inhibitor Whi5. Due to the abundance of Cdk1 and to the fast dynamics of Cdk1 binding to its cyclin targets, the molecular model does not explicitly consider the binding dynamics for Cdk1 and Cln and Clb cyclins The model accounts for the percentage of the activated genes of the G_1_/S regulon (235 genes), as well as for the evolution of five of the expressed genes, Cln1, Cln2, Cln5, Clb6 and Nrm1.

From a numerical viewpoint, the model sums up a set of ordinary differential equation, whose order depends of the chosen phosphorylation configuration. For instance, in case of wild-type cells (that is, 4 functional phosphorylation sites for Swi6, 4 functional/12 total phosphorylation sites for Whi5), we have a set of 264 ordinary differential equations. To simulate these nonlinear dynamics requires the setting of about 100 model parameters/initial conditions.

Essential biological evidence, supporting references, and an outline of the reconstruction and computational representation of the G_1_/S transition model are detailed in Supplementary Notes 1–10. Parameter setting and *in silico* experimental procedures are reported in Supplementary Note 12 and 13, respectively. The parameters are presented in Supplementary Tables 1–7.

### Simulation of the G_1_/S model

Single-cell simulations are carried out in Matlab, by means of the built-in ODE Matlab solver ‘ode15.s'. See Supplementary Table 9 for specific growth model parameters in single-cell simulations. From an implementation viewpoint, all the model parameters are sampled from random distributions before running of a population simulation, thus allowing to simulate different cells for different runs, all belonging to the same population. These parameters include the order of proper expression and the times of weak expression for the molecular players Cln1, Cln2, Cln5, Clb6 and Nrm1 (Supplementary Note 9).

The model provides in output ribosome and protein content as well as all the involved molecular player time courses according to which the following relevant outputs are computed:

- length of timer *T*_1_, when cytoplasmic Whi5 exceeds 50% of total Whi5.
- length of Timer *T*_2_, when cytoplasmic Sic1 exceeds 50% of total Sic1.
- critical size *P*_s_, the protein content at the onset of the budded phase.

A further output is given by the time course of the percentage of activated genes. The Hill function best-fitting such a percentage provides a fourth relevant output: the Hill coefficient aiming at measuring the coherence of the G_1_/S regulon activation.

### Code availability

The MatLab file is available at http://dx.doi.org/10.5281/zenodo.46955. This link allows to obtain all the software, in a zip file, necessary to simulate our model plus a readme file containing the procedure to run the code. All the model parameters are defined in the d1_one_cell.m file. The MatLab version used was the R2014b.

### Populations

Cell populations were obtained according to the following rules:

1. The initial protein content *P*(0) of each cell was drawn from a log-normal distribution of a given average value (*η*_P_) and s.d. (*σ*_P_). Different values of *η*_P_ allowed to build populations of smaller or larger cells, and different values of *σ*_P_ allowed to build populations with a larger or smaller cell protein dispersion.
2. The initial ribosome content *R*(0) was straightforwardly set from *P*(0) using the equation: *R*(0)=*ρ* × *P*(0).
3. The order of proper expression and the times of weak expression for the molecular layers Cln1, Cln2, Clb5, Clb6 and Nrm1 were drawn from chosen log-normal distributions (Supplementary Table 5).
4. All other model parameters reported in Supplementary Tables 1–4 and 6–7 were drawn from log-normal distributions of chosen average values, sharing the same CV.
5. Parameters specific for each simulation of cell population are reported in Supplementary Table 10.

Model complexity and simulation times are discussed in Supplementary Note 24, and Supplementary Fig. 19.

### Sensitivity analysis

For sensitivity analysis, tested parameters were grouped in four sets: Cln3 production and nuclear import (all parameters in Supplementary Table 3); G_1_/S regulon activation (all parameters in Supplementary Table 4 except for Swi6_tot_, Swi4_tot_, Mbp1_tot_ and Whi5_tot_); Cln/Clb function (all parameters in Supplementary Table 6); Sic1 function (all parameters in Supplementary Table 7 except for Sic1_tot_). Taking the standard value as 1, each parameter was either increased (up to 81-fold the standard value, step 3) or decreased (down to 1/81-fold the standard value, step 1/3). The impact of each of these changes in parameter value was tested on four significant outputs: *N*, the Hill coefficient for G_1_/S regulon activation curve, the length of the *T*_1_ and *T*_2_ period and the critical size *P*_s_. The sensitivity analysis was done by varying each parameter individually, associating it to the run of a single-cell deterministic simulation, with the proper order of activation and the weak activation times fixed to their average values, i.e. *β*_Cln1_, *β*_Cln2_, *β*_Nrm1_, *β*_Clb5_, *β*_Clb6_ (Supplementary Table 5). Results and further details are reported in Supplementary Figs 12–15 and Supplementary Notes 19–23.

## Additional information

**How to cite this article:** Palumbo, P. *et al*. Whi5 phosphorylation embedded in the G1/S network dynamically controls critical cell size and cell fate. *Nat. Commun.* 7:11372 doi: 10.1038/ncomms11372 (2016).

## Supplementary Material

Supplementary Information Supplementary Figures 1-19, Supplementary Tables 1-10, Supplementary Notes 1-24 and Supplementary References

Supplementary Movie 1 Role of functional and decoy phosphosites in the control of timing and coherence of multi-target regulatory events in cell cycle progression. Upper and lower left panels of the movie show a schematic cell nucleus containing SBF-Whi5-controlled G1/S regulon genes. Open circles represent non-transcribed genes, while green circle transcribed genes. Right panels represent cumulative probability of the activation of the genes. The upper row represents a configuration of 4 functional/8 decoys sites. The bottom row represents the configuration of 1 functional/8 decoys sites.

## Figures and Tables

**Table 1:** Decomposition of G_1_ variability.

	**G**_**1**_ **noise for λT**_**G1**_	**Noise due to size control**	**Size-independent noise**
WT (exp)	0.55	0.31 (32%)	0.45 (68%)
WT (sim)	0.52	0.23 (19%)	0.47 (81%)
6 × Cln3 (exp)	0.44	0.25 (32%)	0.36 (68%)
6 × Cln3 (sim)	0.33	0.13 (15%)	0.31 (85%)
6 × Cln2 (exp)	0.48	0.30 (39%)	0.37 (61%)
6 × Cln2 (sim)	0.43	0.25 (35%)	0.34 (65%)
6 × Cln3, 6 × Cln2 (exp)	0.37	0.18 (24%)	0.32 (76%)
6 × Cln3, 6 × Cln2 (sim)	0.40	0.10 (6%)	0.39 (94%)

WT, wild type.

Decomposition of G_1_ variability in terms of deterministic size control and a residual that is attributable to molecular noise under the assumption that *λT*_G1_ can be decomposed into the sum of a suitable deterministic function of the initial protein content *f*(*P*(0)) plus a stochastic variable. The chosen function for *f*(*P*(0)) is the hyperbola described in Fig. 3e. The experimental data are from ref. 13.
