# Supplementary material for: Whi5 phosphorylation embedded in the G1/S network dynamically controls critical cell size and cell fate
Source: Nat Commun. 2016 Apr 20;7:11372. doi: 10.1038/ncomms11372 (PMC4843020; doi:10.1038/ncomms11372)
Supplement: Supplementary Information — Supplementary Figures 1-19, Supplementary Tables 1-10, Supplementary Notes 1-24 and Supplementary References [file ncomms11372-s1.pdf]

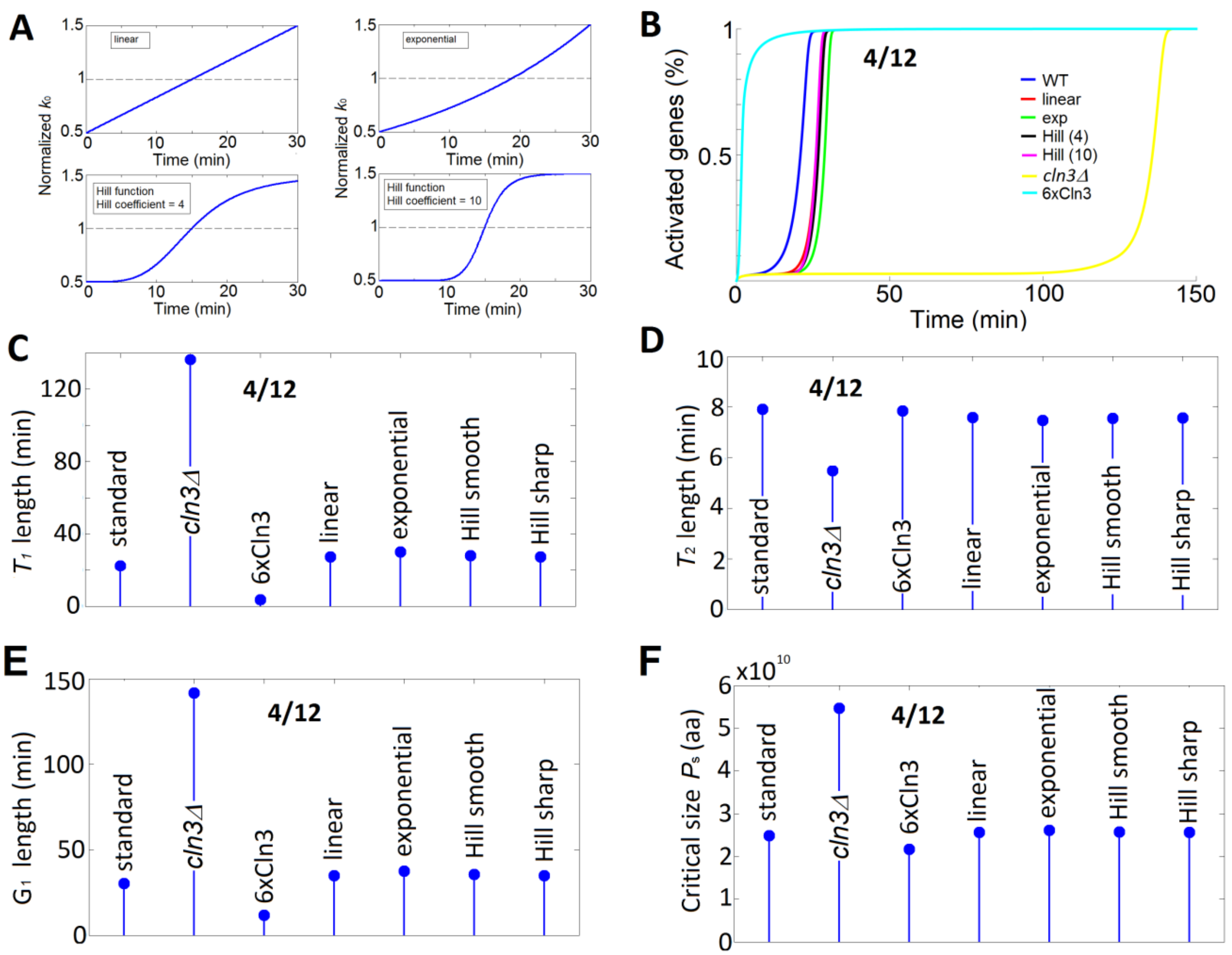

Supplementary Figure 1 – Effect of pattern of accumulation of Cln3 on activation of the G_1/S regulon, on temporal parameters and on critical cell size: the 4/12 phosphorylation scheme. A. Patterns of accumulation of Cln3 over time according to different functions. In the standard model Cln3 concentration is constant (horizontal dashed line). Effect of the pattern of Cln3 accumulation on activation of the G_1/S regulon (panel **B**) and on temporal parameters of the G_1/S transition (panels **C, D, E** refer to T_1, T_2, G_1 respectively) and on the critical cell size (panel **F**). The simulations indicate that each parameter is barely affected by the pattern of Cln3 accumulation. For reference, the same outputs in *cln3Δ* and 6xCln3 virtual cells are reported, showing the major effects provoked by either Cln3 ablation or overexpression. The same behavior is obtained in the 1/12 phosphorylation configuration (Supplementary Fig. 2).

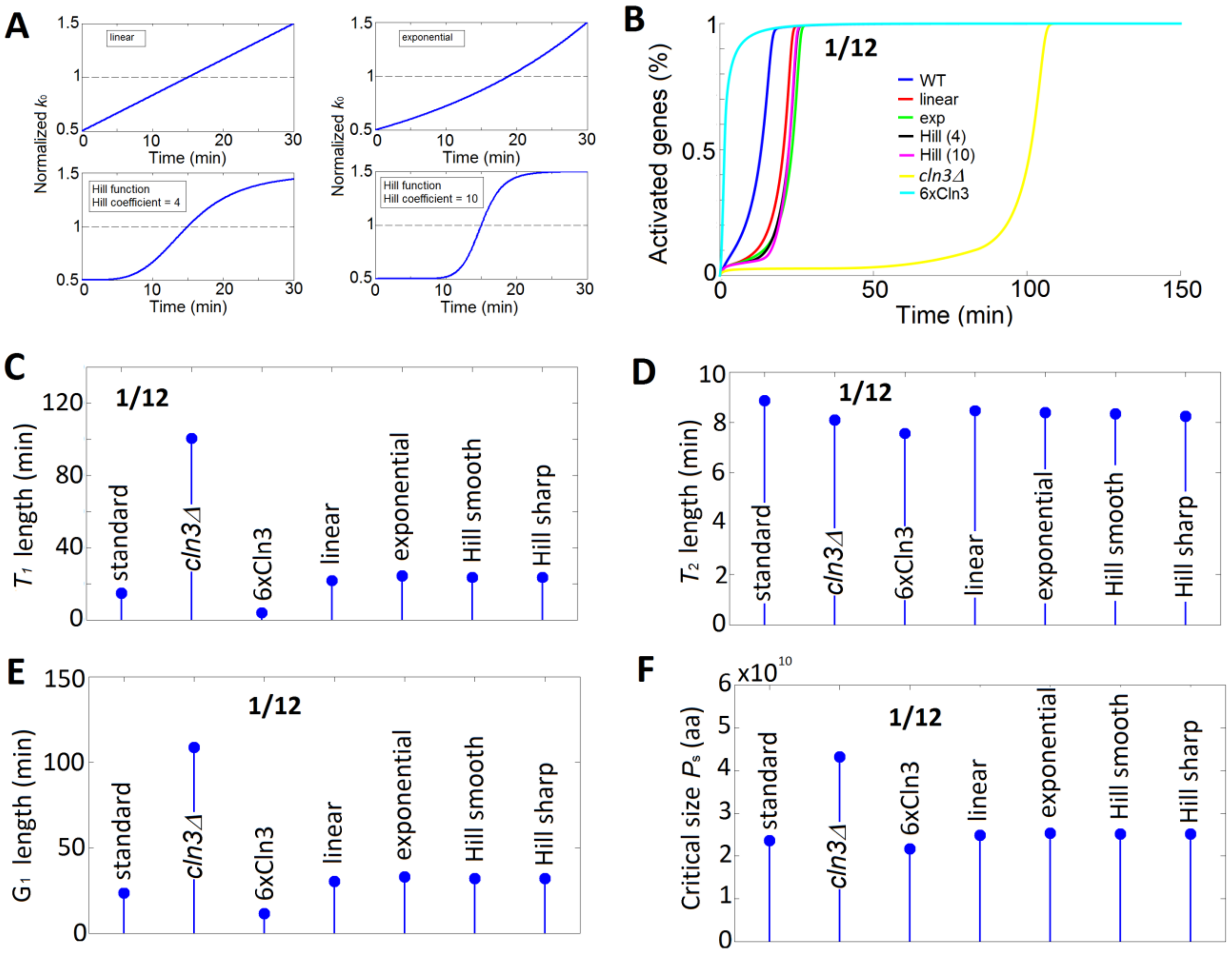

Supplementary Figure 2 – Effect of pattern of accumulation of Cln3 on activation of the G_1/S regulon, on temporal parameters and on critical cell size: the 1/12 phosphorylation scheme. **A.** Patterns of accumulation of Cln3 over time according to different functions. In the standard model Cln3 concentration is constant (horizontal dashed line). Effect of the pattern of Cln3 accumulation on activation of the G_1/S regulon (panel **B**) and on temporal parameters of the G_1/S transition (panels **C, D, E** refer to T_1, T_2, G_1 respectively) and on the critical cell size (panel **F**). The simulations indicate that each parameter is barely affected by the pattern of Cln3 accumulation. For reference, the same outputs in *cln3Δ* and 6xCln3 virtual cells are reported, showing the major effects provoked by either Cln3 ablation or overexpression. The same behavior is obtained in the 4/12 phosphorylation configuration (Supplementary Fig. 1).

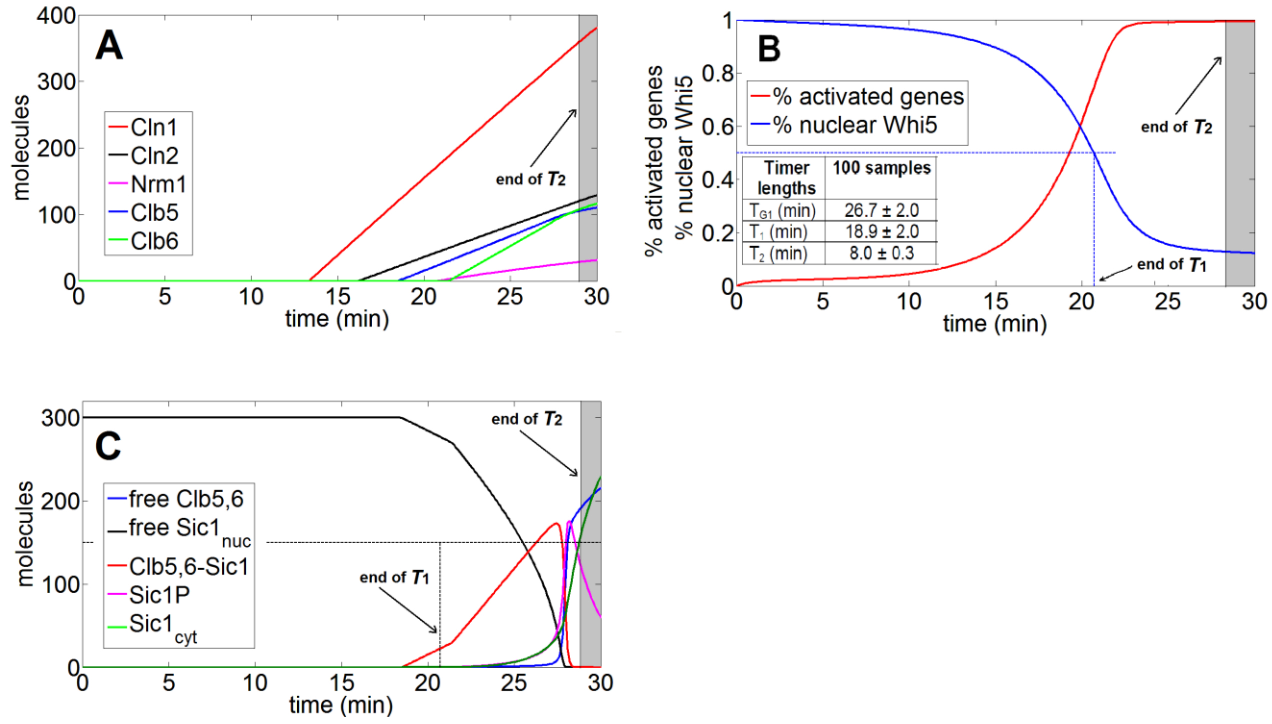

Supplementary Figure 3 - Time course of relevant players in the G$_1$/S transition model. A Time course of the accumulation of molecular players explicitly encoded by the G$_1$/S regulon: predicted dynamics of various cyclins (Cln1, Cln2, Clb5 and Clb6) and of Nmr1, whose timing and extent of production are in agreement with the literature [60,70]. **B** Time course of the fraction of the activated genes of the G$_1$/S regulon (red line) and the fraction of nuclear Whi5 (blue line). The inset reports the statistics for the T_1 and T_2 values, which were derived from a set of 100 independent simulations, each run with the same parameter set provided by the initial conditions and average values of Supplementary Tables 1-4, 6-7, 9. Parameters in Supplementary Table 5 were the only allowed to stochastically vary. This analysis allows to estimate the effect of the stochastic mechanism that is intrinsic in our model, while showing the excellent agreement found between the simulated length of T_1 (18.9 min ± 2.0) and the experimental length of T_1 (20 min) [19]. **C** Time course of the accumulation of the cyclins Clb5,6 and their complexes with the inhibitor Sic1. The level of Clb5,6-Cdk1-Sic1 increases until the amount of Clb5,6 produced overcomes the amount that can be bound by the available Sic1, which therefore acts as a Sic1-dependent titration threshold. Free Clb5,6-Cdk1 is then available and, together with Cln2-Cdk1 (which is already present), starts to phosphorylate Sic1, acting as an AND logic gate. Phosphorylated Sic1 is exported into cytoplasm and degraded. Newly released Clb5,6-Cdk1 powers a feed forward loop that accelerates degradation of Sic1 and releases Clb5,6-Cdk1 in bulk, to activate the onset of DNA replication [24]. The end of the T_2 period (and hence of G$_1$ phase) is set at the time when cytoplasmic Sic1 exceeds 50% of total Sic1.

- The progression of yeast cell cycle requires that several steps, involving a large number of molecular triggers, take place at the appropriate timing and with a sharp synchrony (or **coherence**), as it happens for the onset of DNA replication – from about 200 origins – or for the activation of the transcription of the G1/S regulon from about 200 promoters.
  What is, then, the molecular mechanism that underlies proper timing and coherence?
  In both cases, a cyclin-dependent protein kinase phosphorylates a protein (or a protein complex) that is the master regulator of the controlled function. In the case of G1/S regulon transcription, Cln1,2,3.Cdk1 phosphorylates Whi5 which, when fully phosphorylated, releases its inhibitory binding to the transcription factor SBF, allowing RNA polymerase II to initiate G1/S regulon transcription.

- The protein Whi5 has 12 phosphosites for Cdk1, largely phosphorylated in proliferating cells.

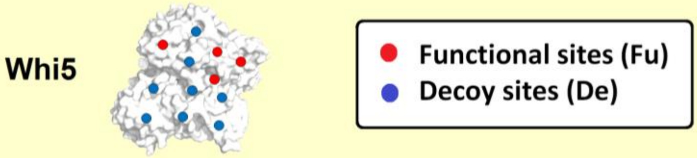

  Four of these sites are Functional (Fu), while eight are Decoy (De): when all 4Fu sites are phosphorylated, there is a conformational change which releases Whi5 from SBF and activates G1/S regulon transcription.

- The phosphorylation of the De sites does not induce conformational changes in Whi5, but it engages the Cln3/Cdk1 activity, diverting it from the phosphorylation of Fu sites.

- The molecular model of the G1/S transition presented in this paper may allow to test the effects that changes in the number of Fu sites or De sites may have in the functional properties: timing and coherence of a trigger molecule.

- The panels below show the activated (green circles) and inactivated (white circles) G1/S regulon genes (235 in amount) at different time instants and different Whi5 Fu/De configurations, clearly showing the coherence determined by the 4Fu/8De configuration

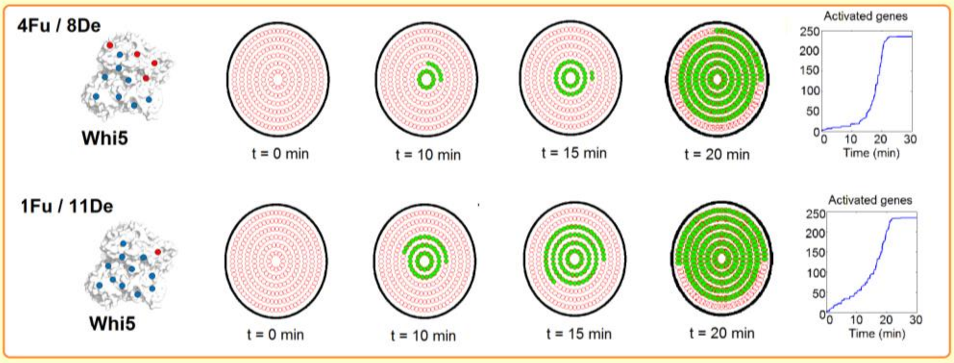

- The panels below show the **gene activation rate** in terms of the probability density function computed from the probability of gene activation for different Fu/De configurations. CV = σ/μ is the Coefficient of Variation, computed as the ratio of the standard deviation σ over the average mean μ of the distribution.

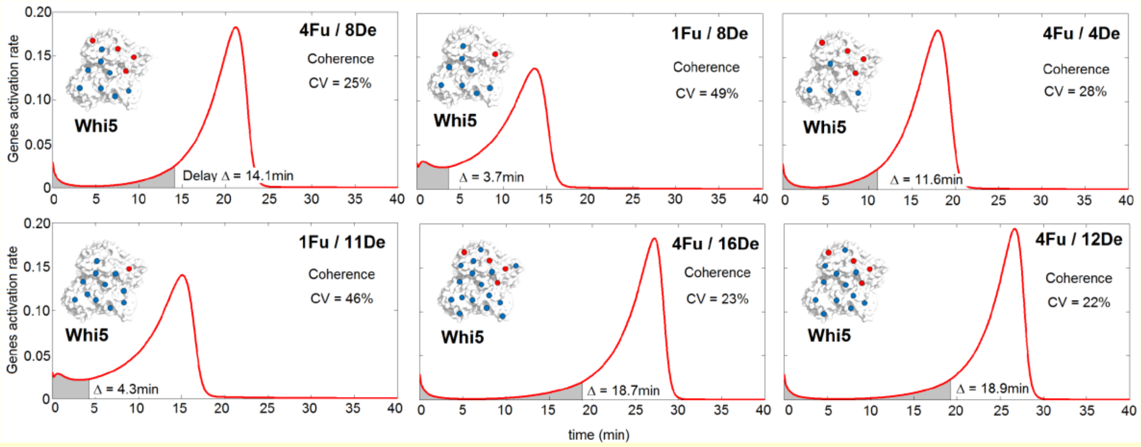

The CV provides an estimate of the loss of coherence in the transcriptional activation response, a more pronounced coherence being associated to a smaller CV. ***Fu sites play a major role***, since the CV substantially decreases by increasing the number of Fu sites (compare 4Fu/8De with 1Fu/8De, 4Fu/16De with 4Fu/12De). ***De sites provide a smaller (though apparent) contribute***, allowing smaller CVs for greater number of De sites, keeping fixed the number of Fu sites (compare for instance 4Fu/4De with 4Fu/16De).

The timing delay Δ is formally defined as the time period from the onset of Cln1,2,3.Cdk1 activity (time 0) to the activation of 10% (i.e. the grey area of the gene activation rate is 0.1).

It is clear that, keeping fixed Fu to 4, the time delay increases by increasing the number of De sites. On the other hand, reducing the number of Fu sites and keeping constant De = 8 substantially reduces the timing delay.

In conclusion: ***the number of Fu sites and De sites affect both functional properties, with Fu sites having a major role on coherence and De sites on time delay.***

Supplementary Figure 4 - Role of functional and decoy phosphosites in the control of timing and coherence of multi-target regulatory events in cell cycle progression (see also Supplementary Movie 1).

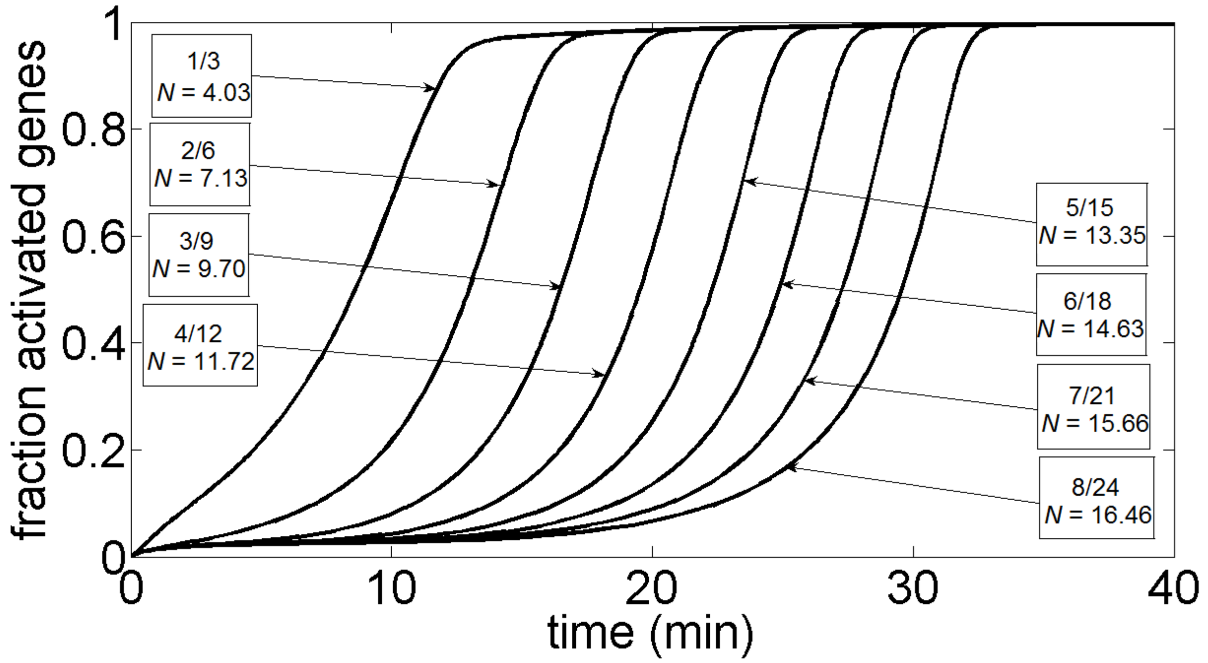

Supplementary Figure 5 - Transcriptional activation of the G_1/S regulon as a function of the phosphorylation scheme. Time course of the fraction of the activated genes of the G_1/S regulon with different phosphorylation configurations. In all configurations the ratio between the "functional" and total sites is 1/3. For each curve the actual ratio and the coefficient of the best fitting Hill curve (*N*) is shown. Initial conditions and parameters can be found in Supplementary Tables 1-4, 6-7, 9.

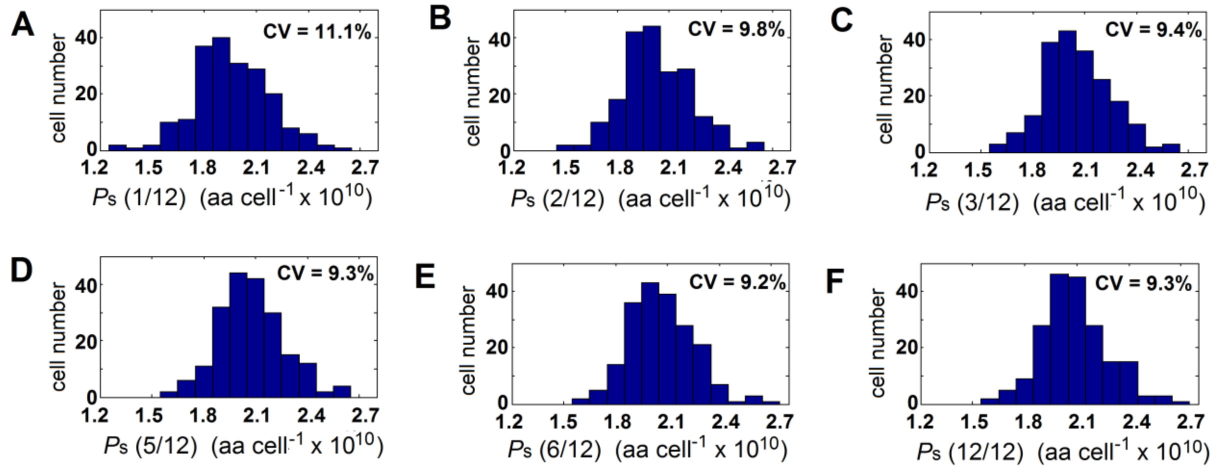

Supplementary Figure 6 - Size variability at the G_1/S transition as a function of phosphorylation configuration. Simulation of the G_1/S transition for a cohort of newborn daughter cells with the *P*(0) size distribution reported in Fig.1G was performed as summarized in Fig.1 with a varying number of functional Whi5 sites to obtain the size distribution of the critical cell size P_s. Different panels here refer to x/12 different Whi5 phosphorylation configurations. It is clear that starting with a CV of 15% at *P*(0), the size variability decreases by increasing the number of functional sites of Whi5 from 1 to 4 and remains constant thereafter (Fig. 1I). Population parameters can be found in Supplementary Table 10.

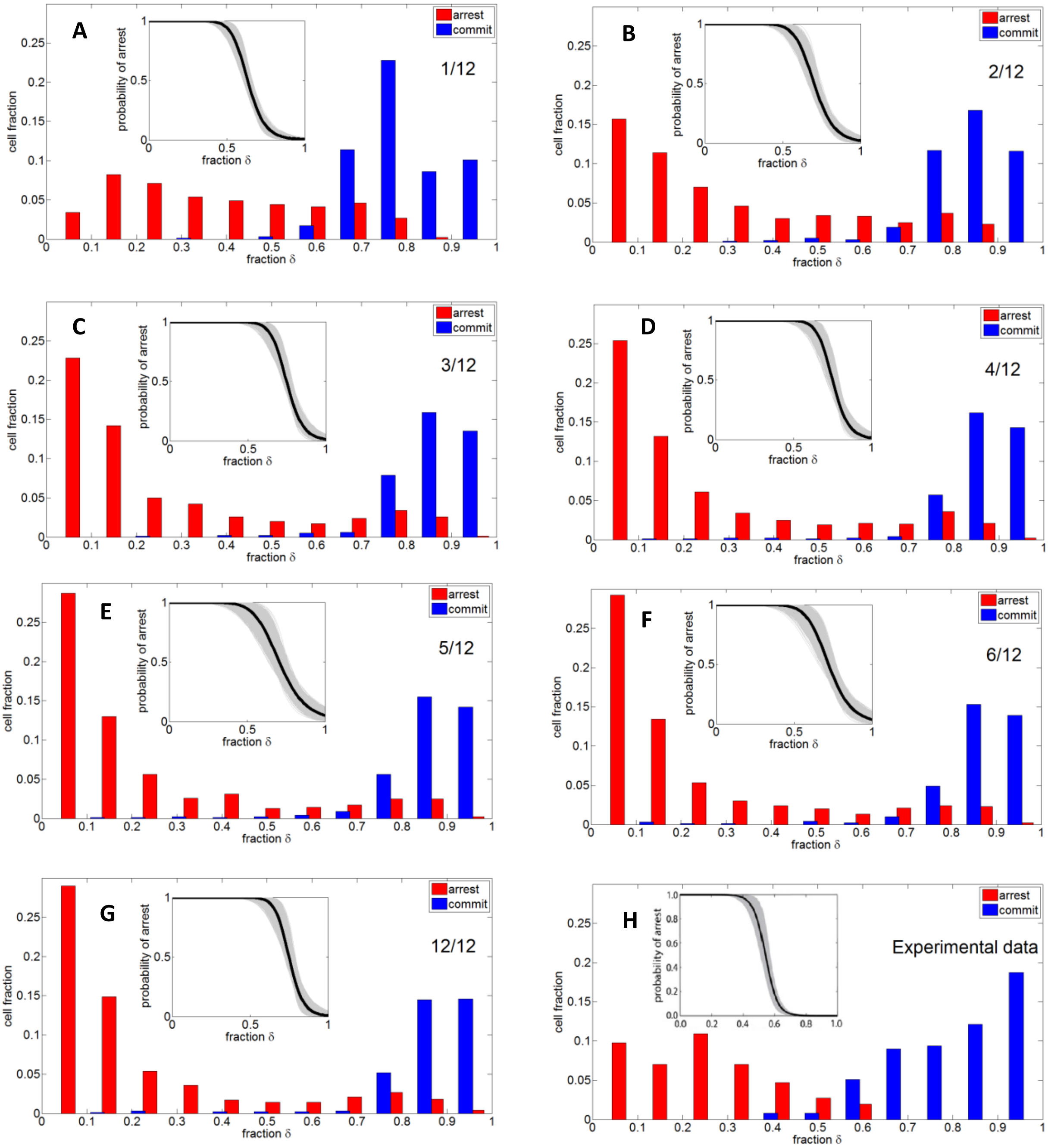

I	Hill coefficient	Median point	95% Hill coeff. Bootstrap	Mean Hill coeff. Bootstrap	Median Hill coeff. Bootstrap
1/12	11.8	0.63	> 14.2	11.7	11.6
2/12	11.1	0.70	> 13.0	10.8	10.7
3/12	14.6	0.75	> 17.6	14.1	13.9
4/12	15.0	0.75	> 19.2	14.7	14.40
5/12	8.4	0.71	> 10.6	8.3	8.2
6/12	10.0	0.72	> 12.5	9.8	9.7
12/12	16.7	0.75	> 21.9	16.5	16.1
Exper.	-	-	> 14	-	-

Supplementary Figure 7 - Effect of the multi-site phosphorylation configuration of Whi5 on α-factor -induced cell cycle arrest. Cell fraction histograms drawn with respect to parameter δ for simulated cells arrested in G_1 phase (red) and committed to bud (blue) for different Whi5 phosphorylation schemes (panels **A-H**). Simulations are run on a range of 1,000 cells. Population parameters can be found in Supplementary Table 10. The insets report the probability of arrest as well as the 95% of bootstrapping iterations. Last panel **I** reports a Table with parameters coming from bootstrapping iterations.

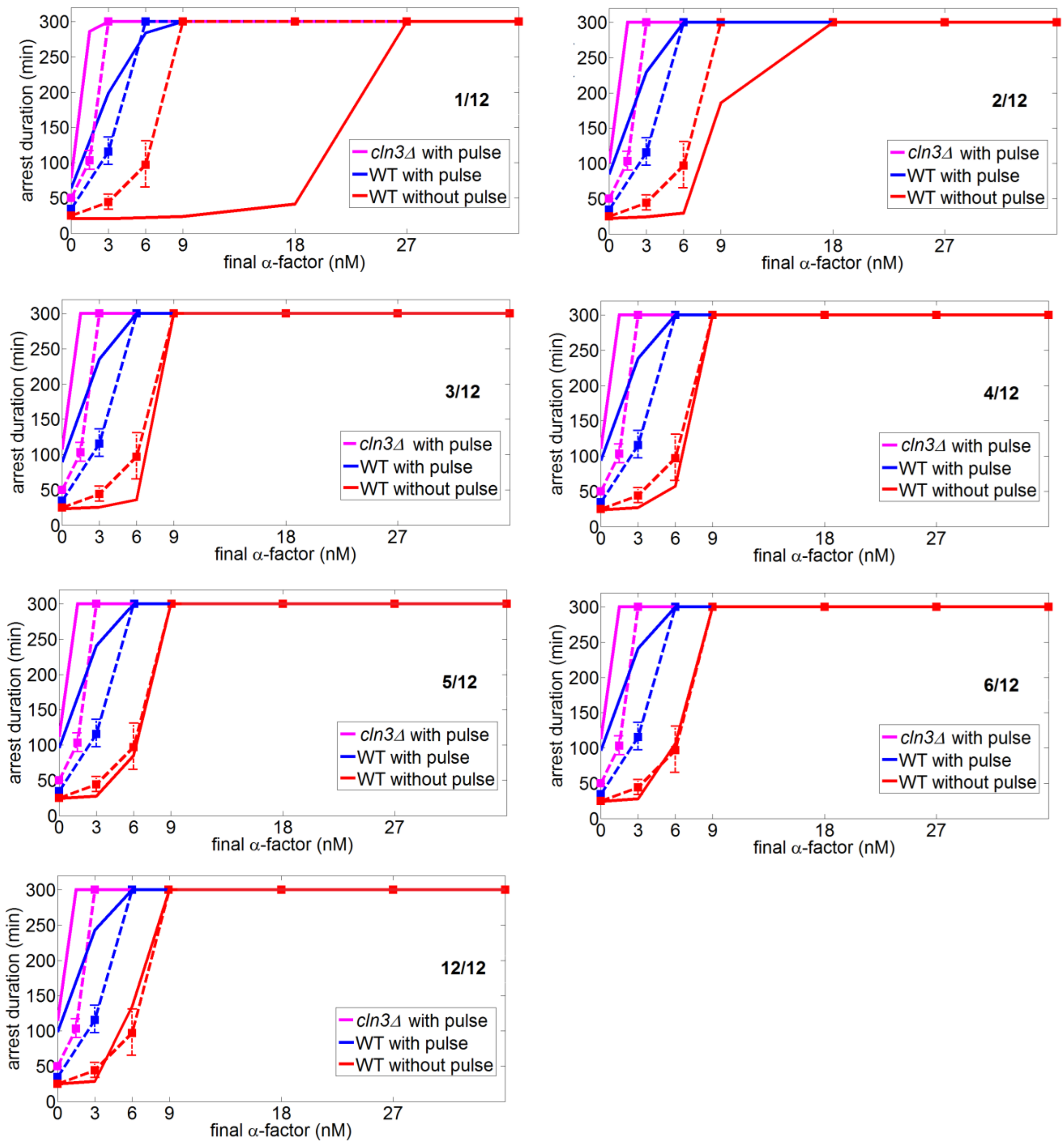

Supplementary Figure 8 - Effect of the multi-site phosphorylation configuration of Whi5 on α-factor - induced hysteresis. Arrest duration in G_1 phase versus a baseline continuous administration of α-factor, according to different Whi5 multi-site phosphorylation schemes. Continuous lines refer to simulations; dashed lines and squares refer to experimental data drawn from [75]. A high concentration, 30 min α-factor pulse is given when a very low fraction of Whi5 is removed from the nucleus (< 2%). The maximum waiting time has been set equal to 300min in simulation, according to the experimental framework of [20,75]. Different panels refer to x/12 different Whi5 phosphorylation configurations. Initial conditions and parameters can be found in Supplementary Tables 1-4, 6-7, 9.

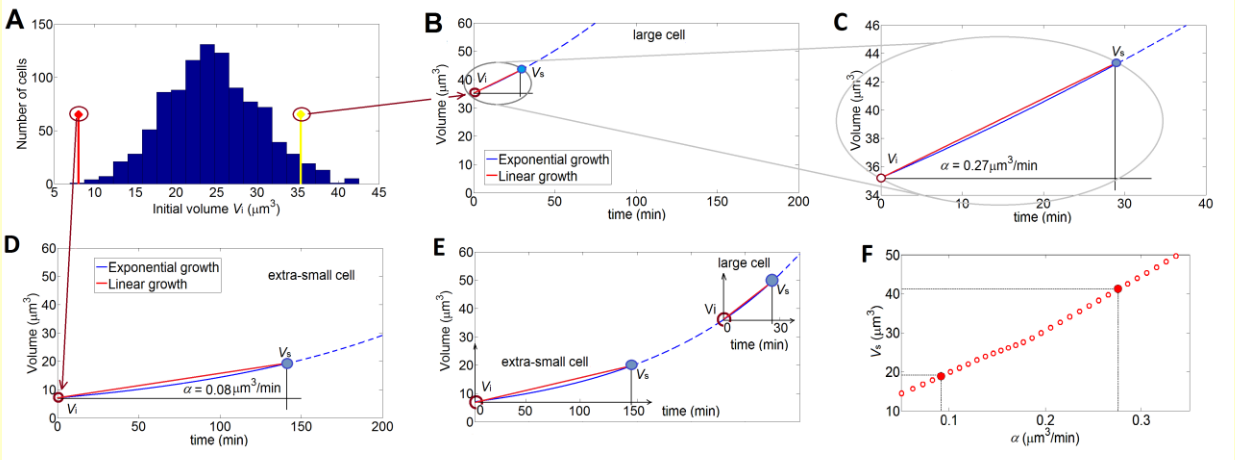

- Consider the population of cells of Ferrezuelo et al. growing in glucose environment, whose statistics are reported in Table S6 of [62]. For the ease of the Reviewer we have redrawn the distribution of initial volumes Vi in panel **A** according to mean value and coefficient of variation given in Table S6. The red and yellow spikes refer to an extra-small and a large cell hypothetically sampled from the population, with initial volumes V_i = 8 μm^3, and V_i = 35 μm^3 respectively.

- We simulate exponential growth of the 2 cells chosen in panel **A**: panels **B** and **D** refer to the volume time-course of the large and extra-small cell, respectively. Both cells grow with the same exponential growth rate (λ = 0.0063 min^{-1}). The continuous blue line refers to the cell evolution in exponential growth; the white circle indicates the initial volume, the blue circle indicates V_s the volume at the end of Timer T_1, according to Ferrezuelo et al. notation [62]. To make the comparison between the two different cells we have kept the same time scale and the same volume scale. This way it is apparent that the extra-small cell takes a longer time to exit timer T_1, about 140 min, than the large cell that takes about 30 min: indeed, larger cells have smaller T_1 periods. The dotted blue line refers to the time course of the cell volume after the exit from T_1 period.

- According to the definition of α, the linear growth rate in G_1 given by [62], the extra-small cell has α = 0.08 μm^3 min^{-1}, whilst the large cell has α = 0.27 μm^3/min. For the large cell the linear growth rate is quite undistinguishable from the exponential rate, because of the small T_1 period, panel **C**.

- To make it more evident that the extra-small and large cells share the same exponential growth rate, we have reported their volume evolution in a unique figure, panel **E**.

- Panel **F** correlates the volume Vs to the linear growth rate α for cells of different initial size, according to the same exponential growth rate. Red circles denote the initial volumes of the extra-small and large cells detailed above.

Supplementary Figure 9 – Linear vs exponential growth.

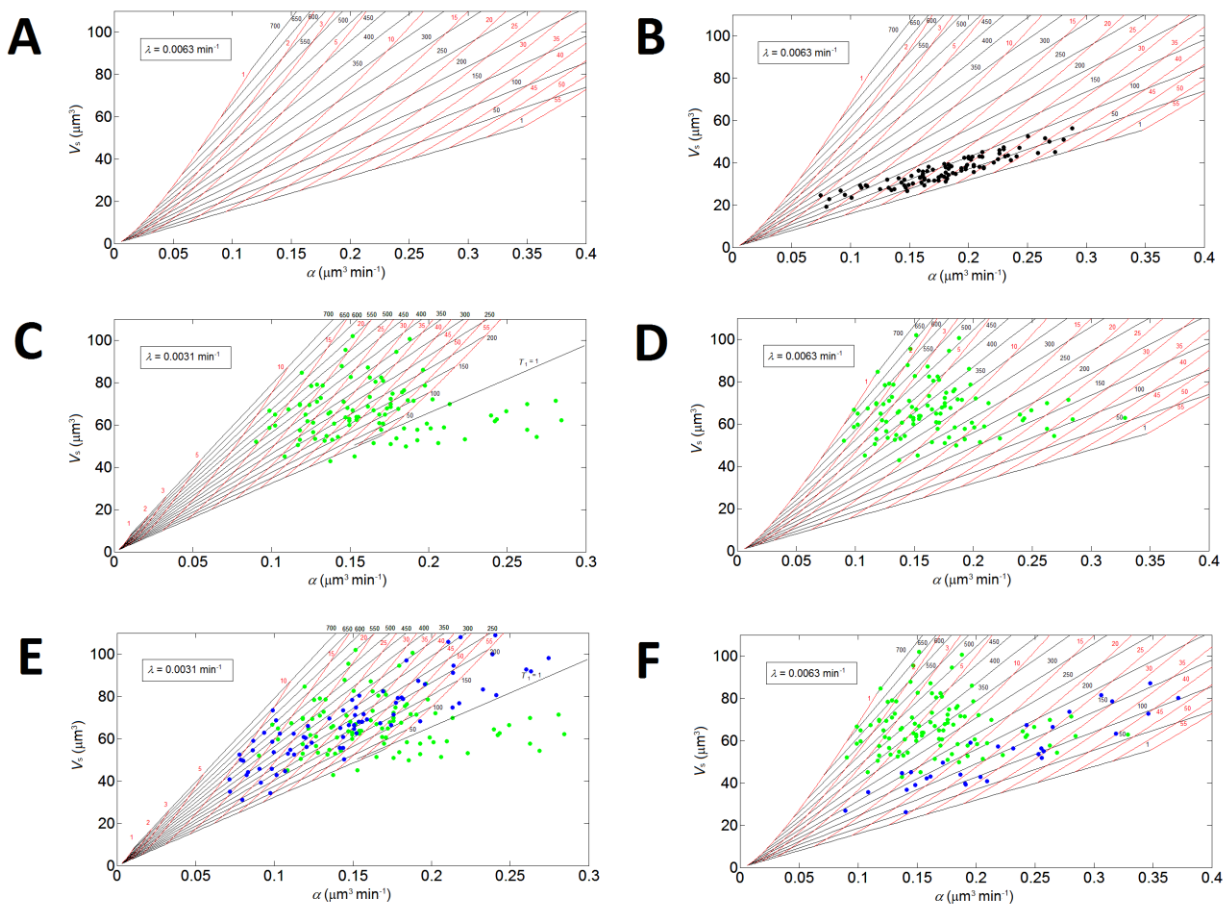

Supplementary Figure 10 – Critical size vs linear growth rate plots. A. Grid of curves at fixed T_1 length (black lines) and at fixed initial volume V_i (red lines), drawn on the 'V_s-versus-α' plot for an exponential growth rate λ of 0.0063 min^{-1}. **B.** WT experimental cells from [62], reported on a grid at λ of 0.0063 min^{-1}. **C.** *ydj1Δ* experimental cells from [62], reported on a grid at λ of 0.0031 min^{-1}. **D.** *ydj1Δ* experimental cells from [62], reported on a grid at λ of 0.0063 min^{-1}. **E.** *ydj1Δ* experimental (from [62], green) and simulated (cluster 1, light blue) cells reported on a grid at λ of 0.0031 min^{-1}. **F.** *ydj1Δ* experimental (from [62], green) and simulated (cluster 2, blue) cells reported on a grid at λ of 0.0063min^{-1}.

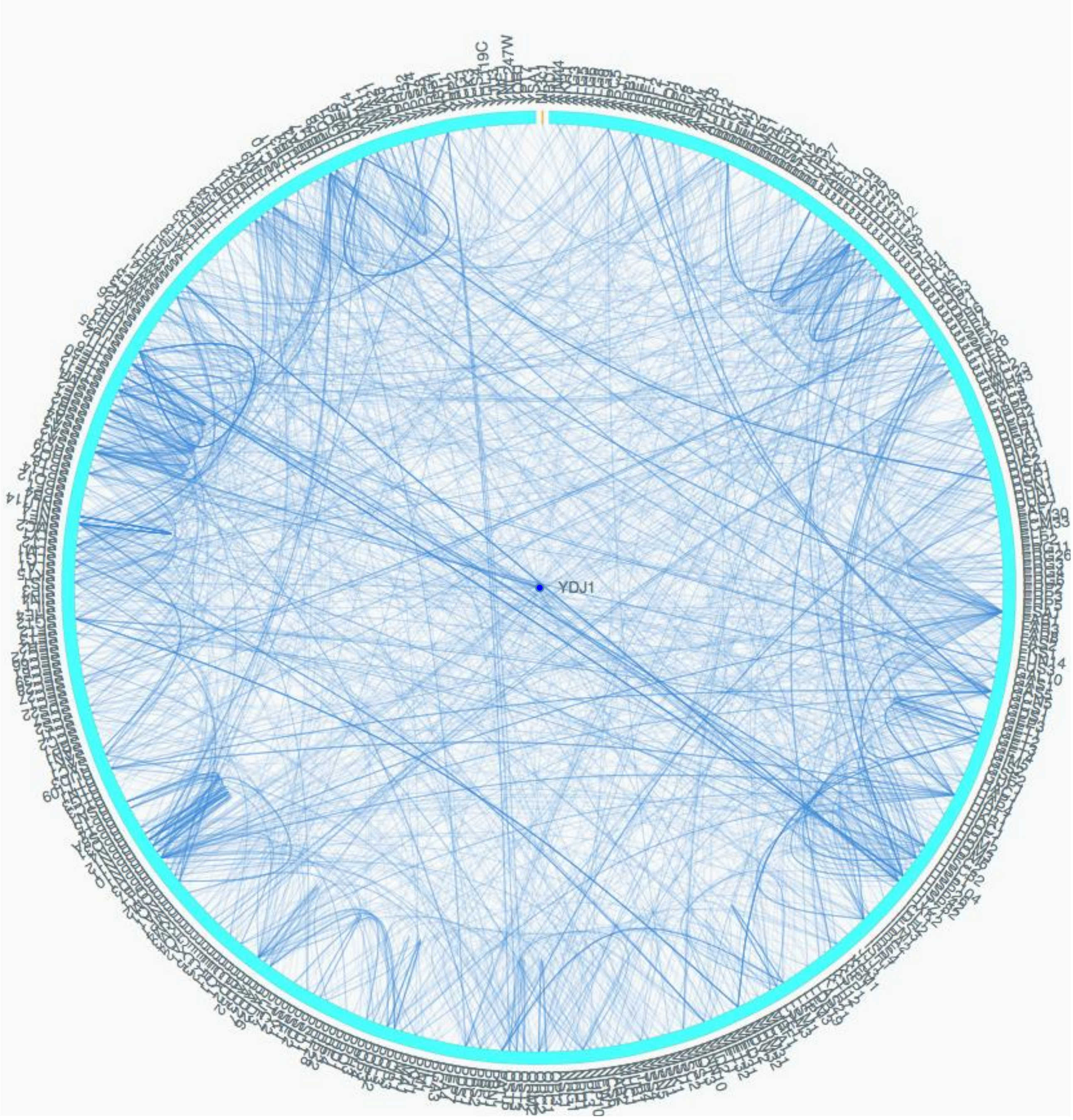

Supplementary Figure 11 – The Ydj1 interactome.

Cln3 production and nuclear import

G1/S regulon activation

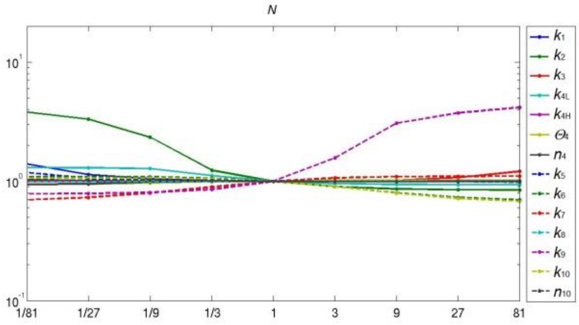

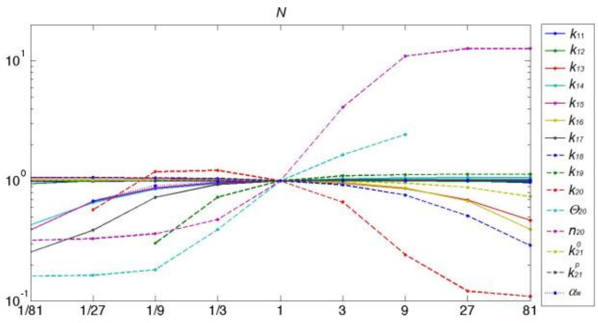

Cln/Clb functions

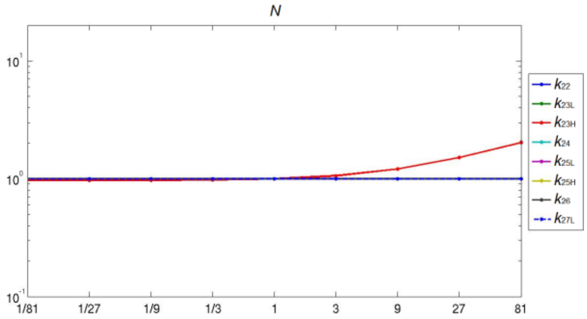

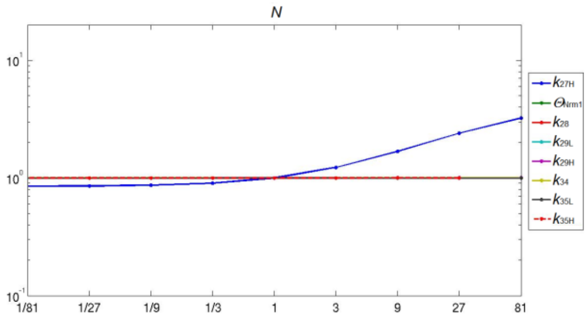

Sic1 functions

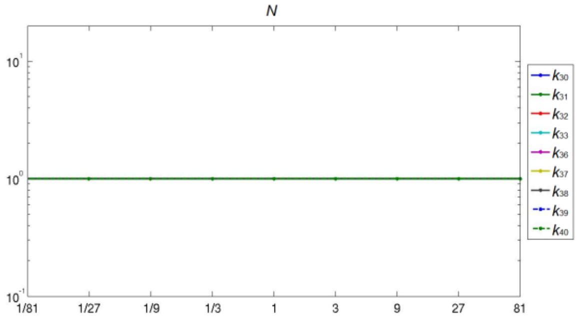

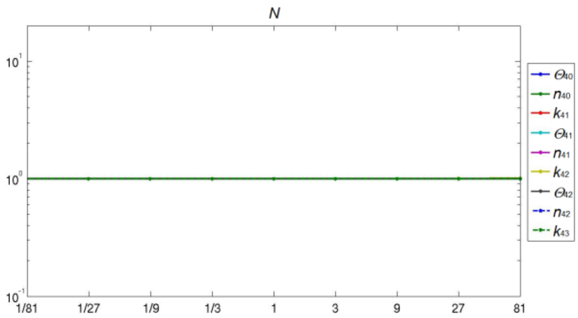

Supplementary Figure 12 - Sensitivity analysis for the coherence of activation (*N*).

Cln3 production and nuclear import

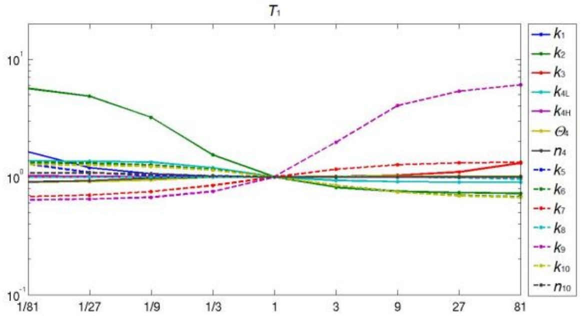

G_1/S regulon activation

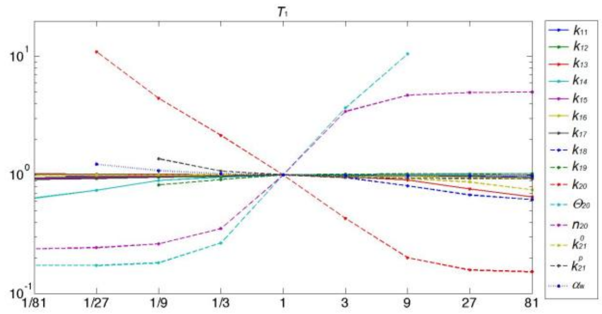

Cln/Clb functions

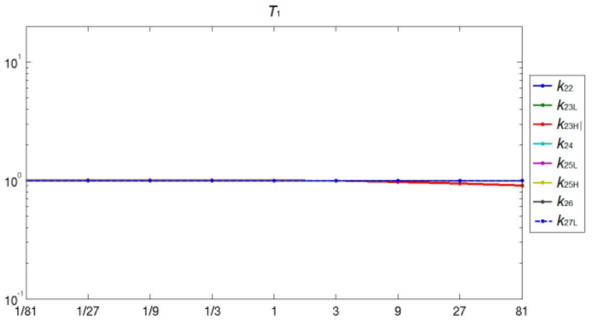

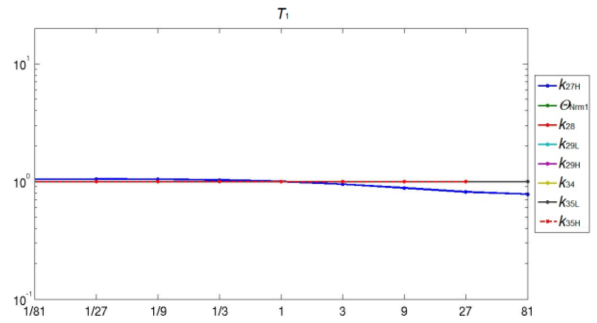

Sic1 functions

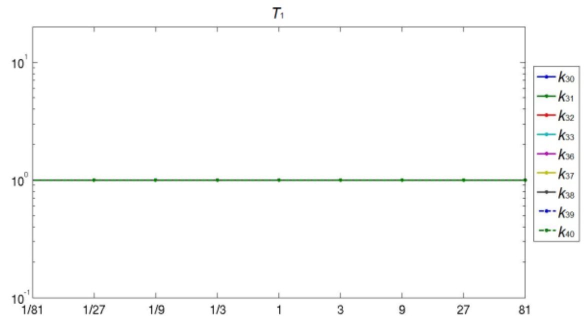

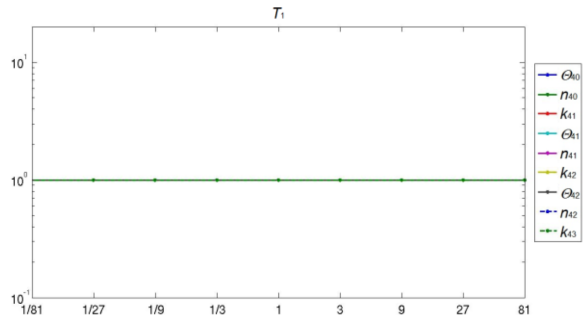

Supplementary Figure 13 - Sensitivity analysis for the length of Timer T_1.

Cln3 production and nuclear import

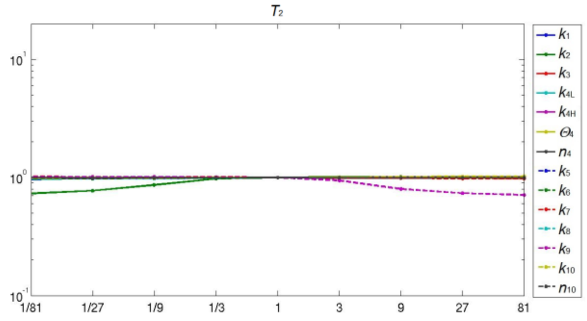

G_1/S regulon activation

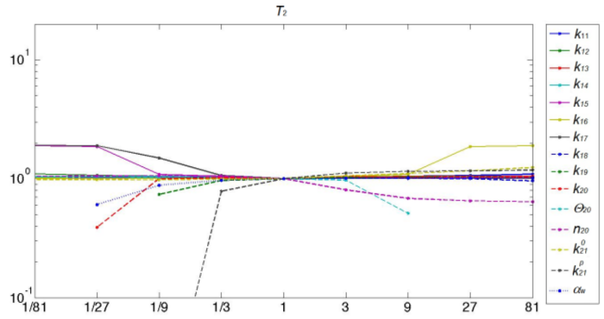

Cln/Clb functions

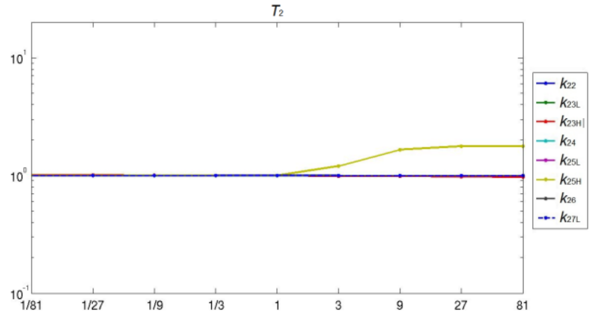

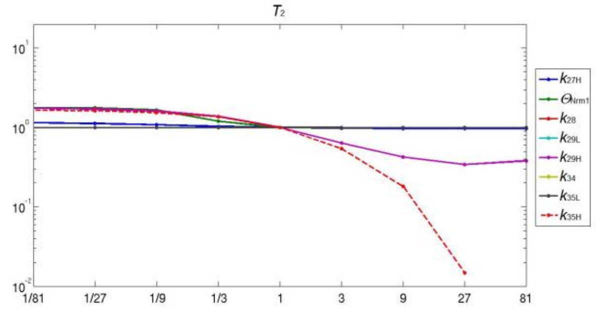

Sic1 functions

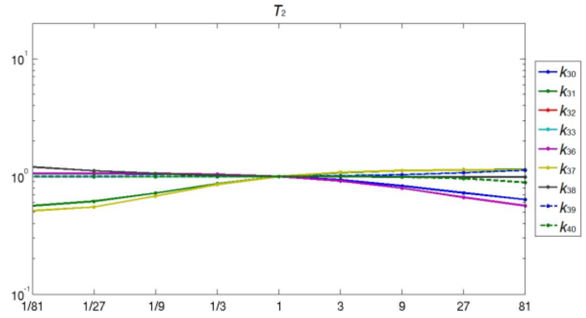

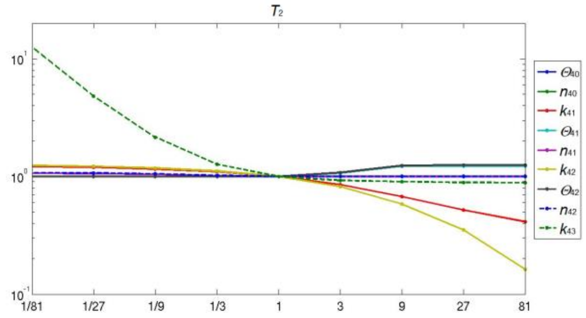

Supplementary Figure 14 - Sensitivity analysis for the length of Timer T_2.

Cln3 production and nuclear import

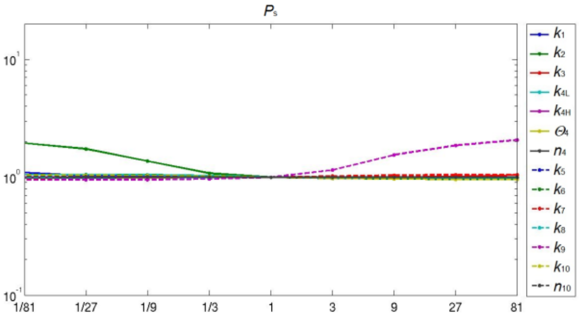

G$_1$/S regulon activation

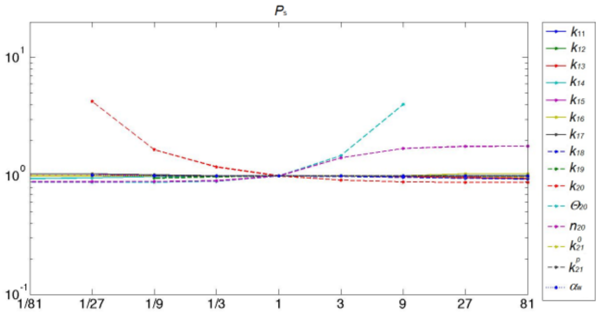

Cln/Clb functions

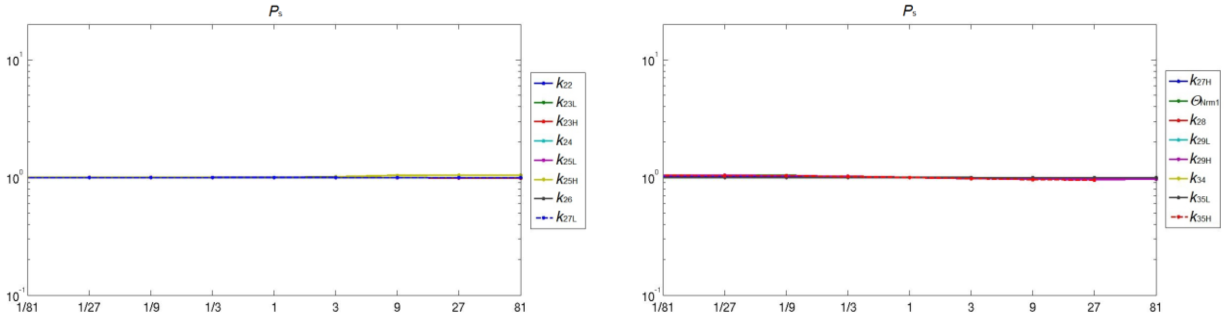

Sic1 functions

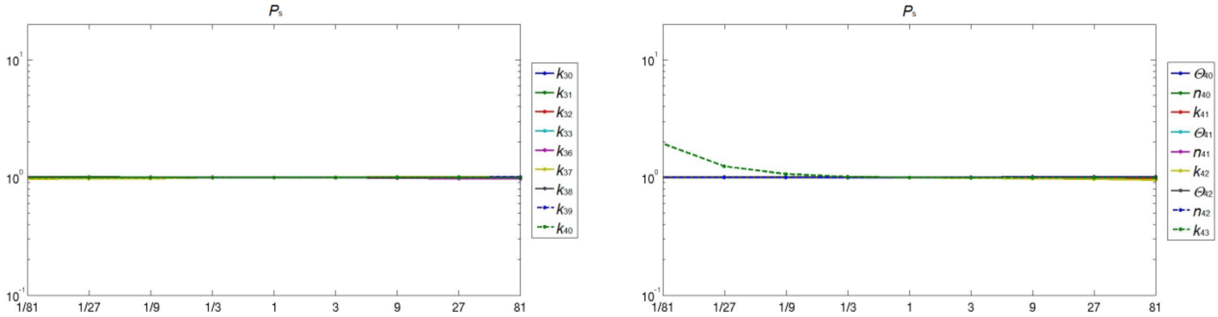

Supplementary Figure 15 - Sensitivity analysis for the critical size P_s.

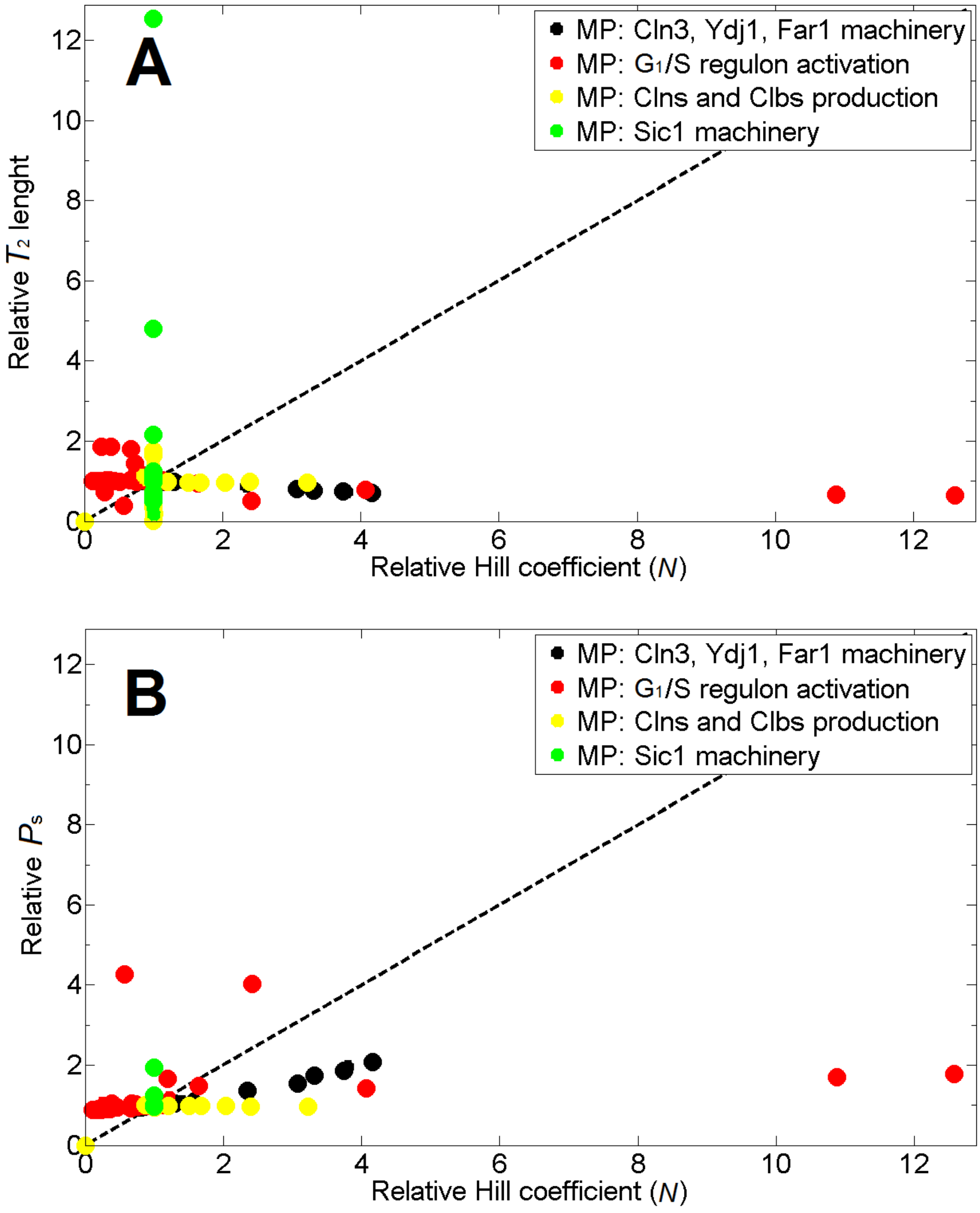

Supplementary Figure 16 - Correlation analysis of the changes in T_2 and P_S as a function of the changes in the coherence *N*. Each point in panels **A-B** refers to a single-cell simulation in which one model parameter - color-coded by functional class - was altered. The X and Y axes are the relative output values, with the value of the standard parameter set to 1. Little or no correlation was observed in the T_2 *vs*. *N* and P_s *vs*. *N* plots, while good correlation was observed in the T_1 vs *N* plot for parameters involved in Cln3 and Far1 production and G_1/S regulon activation (Fig.5C).

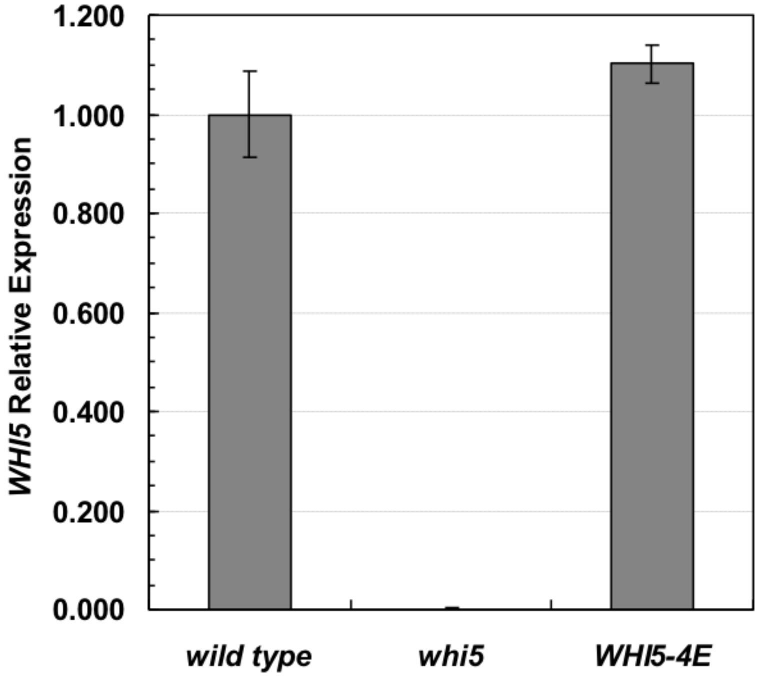

Supplementary Figure 17 – qRT-PCR analysis of WHI5 transcripts. Values plotted (means ± SDs of two independent experiments) are relative to the *WHI5* expression level measured in the wild type reference strain. *TAF10* was used as housekeeping gene for normalization.

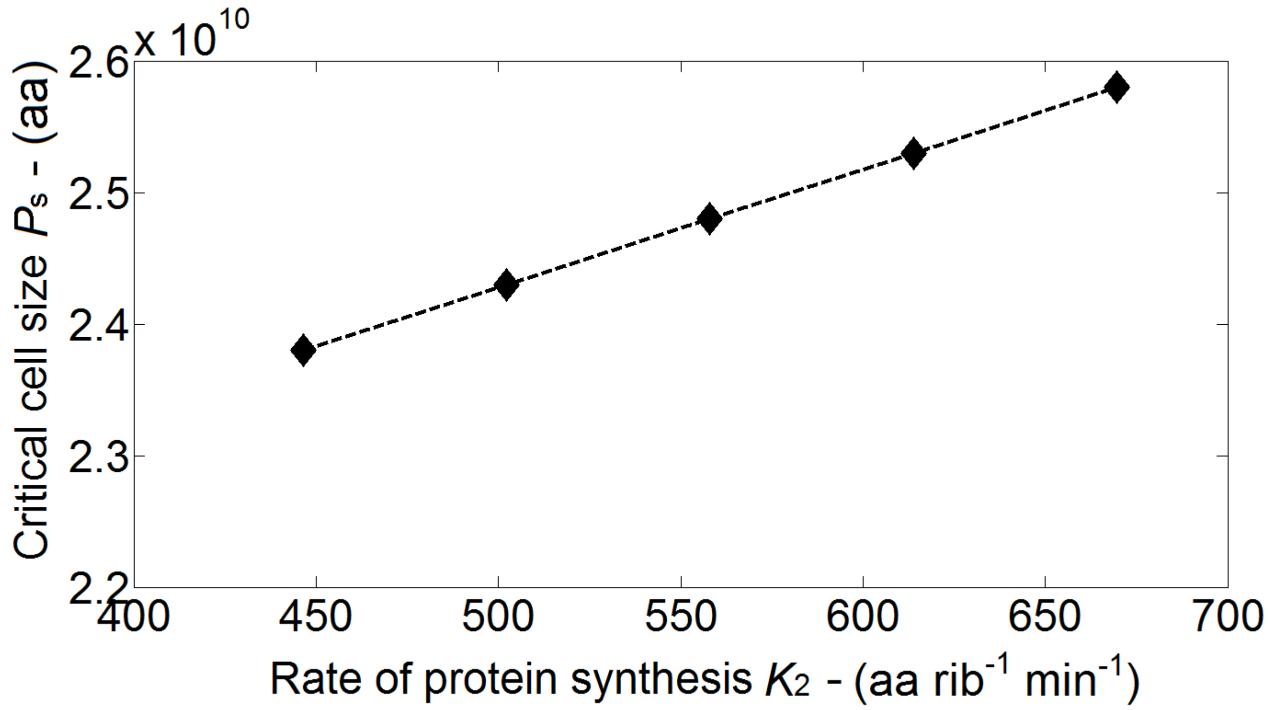

Supplementary Figure 18 – Critical size vs protein synthesis correlation. Cell growth contributes to nutritional modulation of cell size at *START*. Cell growth affects cell cycle progression at many different levels. A sizable part of energy and of building blocks utilized in cellular processes is used for the biosynthesis of informational and structural macromolecules, i.e., RNA and proteins[76]. Let's now assume that two cells are born with the same size in two different growth media: a rich medium supporting fast macromolecular syntheses (and hence a fast growth rate) and a poor medium supporting slow macromolecular syntheses (and hence a slow growth rate). After a given time interval, the cell grown in rich medium will be larger than the cell grown in poor medium. Accordingly, our model (that includes cell growth) predicts that critical cell size becomes larger as a cell increases its rate of protein synthesis (as reported in the present figure). We expect that critical cell size originates as an emergent property of the complex network described in our model (which includes the rate of protein synthesis) and its regulation by nutrients and signaling pathways.

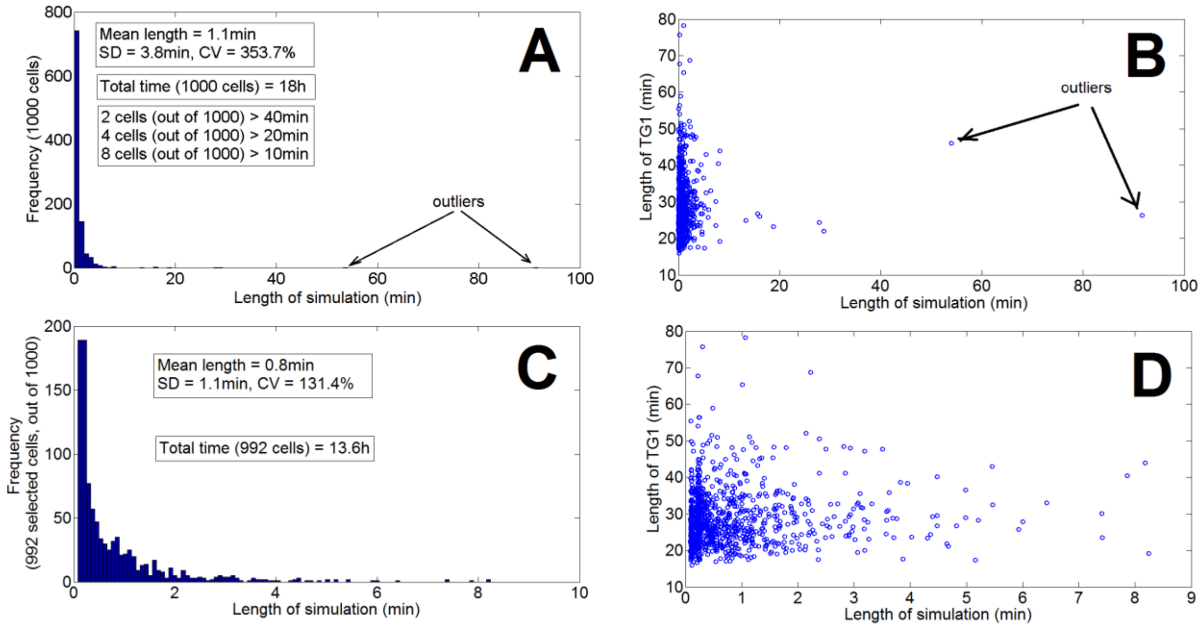

Supplementary Figure 19 – Computational time distribution. Computational time distribution for a single cell simulation (panel **A**). Panel **C** refers to the same population without the 8 higher times. Panels **B** and **D** reports the correlation between timer G_1 length and simulation times for the distributions reported in panels **A** and **C** respectively. Population parameters can be found in Supplementary Table 10.

Supplementary Table 1 - Initial conditions of the G_1/S transition model.

Name	Unit	Value	Description
$P(0)$	[aa]	$[0.5 - 3.2]*10^{10}$	Initial protein content
$R(0)$	[rib]	$[0.6 - 4.3]*10^{5}$	Initial number of ribosomes
$Cln3_{ER}(0)$	[mo]	k_0*P(0)	Newborn cells have all Cln3 in the ER
$Cln3Ydj1_{ER}(0)$	[mo]	0	
$Cln3Ydj1_{nuc}(0)$	[mo]	0	
$Cln3_{nuc}(0)$	[mo]	0	
Cln3Far1(0)	[mo]	0	Newborn cells have the total amount of Far1 free of Cln3
Far1(0)	[mo]	$Far1_{tot}$	
Swi6Swi4(0)	[mo]	0	Newborn cells have the total amount of Swi6, Swi4 and Mbp1 free of any binding, i.e.: Swi6(0) = $Swi6_{tot}$, Swi4(0) = $Swi4_{tot}$, Mbp1(0) = $Mbp1_{tot}$,
Swi6Swi4Whi5(0)	[mo]	0	
Swi6Mbp1(0)	[mo]	0	
Whi5(0)	[mo]	$Whi5_{tot}$	Newborn cells have the total amount of Whi5 not-phosphorylated and free of any binding
Whi5-$P^i(0)$, $i = 1, \ldots, n_{w1}$	[mo]	0	
$P^d_{-1}(0)$	-	1	Newborn cells have MBF-affine genes all free of transcription factors
$P^d_i(0)$, $i = 1, \ldots, n_s$	-	0	
$P^t_{-1,-1,-1}(0)$	-	1	Newborn cells have SBF-affine genes all free of transcription factors
$P^d_{j1,j2,j3}(0)$, $(j_1,j_2,j_3) \neq (-1,-1,-1)$	-	0	
Cln1(0)	[mo]	0	Newborn cells have no molecules of Cln1, Cln2, Nrm1, Clb5, Clb6
Cln2(0)	[mo]	0	
Nrm1(0)	[mo]	0	
Clb5(0)	[mo]	0	
Clb6(0)	[mo]	0	
Clb5Sic1(0)	[mo]	0	Newborn cells have the total amount of Sic1 not-phosphorylated and free of any binding
Clb6Sic1(0)	[mo]	0	
Clb5Sic1-P(0)	[mo]	0	
Clb6Sic1-P(0)	[mo]	0	
$Sic1_{nuc}(0)$	[mo]	$Sic1_{tot}$	
Sic1-P(0)	[mo]	0	

As far as the ‘Unit’ column of the following tables, [rib] stands for number of ribosomes and [mo] stands for number of molecules.

Supplementary Table 2 - Growth parameters of the G_1/S transition model.

Name	Unit	value	Description
K_1	[min^{-1}]	1.0	Ribosome production rate
ρ	[rib aa^{-1}]	[1.0 – 1.35]*10^{-5}	Ribosome-over-protein ratio
D_1	[min]	4000	Ribosome degradation time constant
K_2	[aa rib^{-1} min^{-1}]	[340 - 560]	Protein production rate per ribosomes
D_2	[min]	3000	Protein degradation time constant
h	-	0.07	Nuclear-over-cell volume ratio
H	[aa L^{-1}]	7.1*10^{23}	Protein-over-cell volume ratio
k_0	[mo aa^{-1}]	3.0*10^{-8}	$Cln3_{tot}$-over-proteins ratio

Different growth conditions account for different strains or environment conditions; therefore, some parameters are referred to in terms of intervals they may belong to.

Supplementary Table 3 - Cln3 production and nuclear import parameters

Name	Unit	Value	Description
k_1	[min^{-1}]	100	Rate constant for $Cln3Ydj1_{ER}$ formation
k_2	[min^{-1}]	1	Cln3 diffusion coefficient from cytoplasm into ER
k_{3M}	[mo/min]	200	Cln3 diffusion rate from ER into cytoplasm for values of $Cln3_{ER} > \Theta_3$
Θ_3	[mo]	10	Threshold for Cln3 diffusion rate from ER into cytoplasm
k_{4L}	[min^{-1}]	3	Cln3Ydj1 diffusion from ER into the nucleus for small values of $Cln3Ydj1_{ER}$
k_{4H}	[min^{-1}]	200	Cln3Ydj1 diffusion from ER into the nucleus for high values of $Cln3Ydj1_{ER}$
Θ_4	[mo]	180	Threshold for low/high values of Cln3Ydj1 diffusion from ER into nucleus
n_4	-	30	Hill coefficient for Cln3Ydj1 diffusion from ER into nucleus
k_5	[min^{-1}]	100	Rate constant for Cln3Ydj1 dissociation in the nucleus
k_6	[min^{-1}]	25	Rate constant for Cln3Far1 dissociation
k_7	[mo^{-1} L min^{-1}]	3.2*10^{-15}	Rate constant for Cln3Far1 formation
k_8	[min^{-1}]	0.0015	Ydj1-independent Cln3 diffusion coefficient from cytoplasm into nucleus
k_9	[min^{-1}]	0.4	Cln3 diffusion coefficient from nucleus into cytoplasm
k_{10}	[min^{-1}]	1	Maximum degradation rate for Far1
n_{10}	-	10	Hill coefficient for Far1 degradation rate
$Far1_{tot}$	[mo]	240	Total amount of Far1

Supplementary Table 4 - Input parameters for G_1/S regulon activation

Name	Unit	Value	Description
k_{11}	[min^{-1}]	25	Rate constant for Swi6Swi4 dissociation
k_{12}	[mo^{-1} L min^{-1}]	$1.6*10^{--15}$	Rate constant for Swi6Swi4 formation
k_{13}	[min^{-1}]	2.5	Rate constant for Swi6Swi4Whi5 dissociation
k_{14}	[mo^{-1} L min^{-1}]	$1.6*10^{--14}$	Rate constant for Swi6Swi4Whi5 formation. It reduces to $1.6*10^{-15}$ for mutant *whi5*^{4E}
k_{15}	[mo^{-1} L min^{-1}]	$1.6*10^{--15}$	Rate constant for Swi6Mbp1 formation
k_{16}	[min^{-1}]	25	Rate constant for Swi6Mbp1 dissociation
k_{17}	[mo^{-1} L min^{-1}]	10^{--17}	Binding coefficient to MBF-affine genes of Swi6Mbp1
k_{18}	[mo^{-1} L min^{-1}]	10^{--17}	Binding coefficient to SBF-affine genes of Swi6Swi4
k_{19}	[mo^{-1} L min^{-1}]	10^{--17}	Binding coefficient to SBF-affine genes of Swi6Swi4Whi5
k_{20}	[L mo min^{-1}]	$2.4*10^{--12}$	Maximum phosphorylation rate for Swi6 and Whi5
Θ_{20}	[mo]	1000	Threshold for Cln1,2,3 in Swi6 and Whi5 phosphorylation
n_{20}	-	2	Hill coefficient for Swi6 and Whi5 phosphorylation
N_{20}	[mo]	100	Minimum leakage factor for Swi6 and Whi5 phosphorylation
k_{21}^0	[min^{-1}]	0.07	Diffusion rate of Whi5 from the nucleus into the cytoplasm
k_{21}^i, $i = 1,\ldots, n_{w1}$	[min^{-1}]	0. 7	Diffusion rate of Whi5-P^i from the nucleus into the cytoplasm
α_w	[min^{-1}]	5	Rate constant for Whi5 dissociation from the gene, after activation. It reduces to 0.1 for the case of mutant Whi5-4E
$Swi6_{tot}$	[mo]	300	Total amount of Swi6
$Swi4_{tot}$	[mo]	200	Total amount of Swi4
$Mbp1_{tot}$	[mo]	200	Total amount of Mbp1
$Whi5_{tot}$	[mo]	200	Total amount of Whi5

Supplementary Table 5 - Proper activation orders and weak activation times

Name	Unit	Value	Description
$<\beta_{Cln1}>$	-	5	Average value for the activation order of Cln1 within the class C genes
$<\beta_{Cln2}>$	-	30	Average value for the activation order of Cln2 within the class A genes
$<\beta_{Nrm1}>$	-	110	Average value for the activation order of Nrm1 within the class A genes
$<\beta_{Clb5}>$	-	45	Average value for the activation order of Clb5 within the class B genes
$<\beta_{Clb6}>$	-	20	Average value for the activation order of Clb6 within the class C genes
σ_{Cln1}	-	10	Standard deviation for the activation order of Cln1
σ_{Cln2}	-	10	Standard deviation for the activation order of Cln2
σ_{Nrm1}	-	10	Standard deviation for the activation order of Nrm1
σ_{Clb5}	-	5	Standard deviation for the activation order of Clb5
σ_{Clb6}	-	2	Standard deviation for the activation order of Clb6
$<\Delta_{Cln1}>$	[min]	30	Average value for the weak activation time of Cln1
$<\Delta_{Cln2}>$	[min]	30	Average value for the weak activation time of Cln2
$<\Delta_{Nrm1}>$	[min]	60	Average value for the weak activation time of Nrm1
$<\Delta_{Clb5}>$	[min]	60	Average value for the weak activation time of Clb5
$<\Delta_{Clb6}>$	[min]	60	Average value for the weak activation time of Clb6
σ_{Δ}	[min]	10	Standard deviation for the weak activation time of Cln1,2, Nrm1, Clb5,6

Supplementary Table 6 - Input parameters for Cln1,2, Clb5,6 and Nrm1 dynamics

Name	Unit	Value	Description
k_{22}	[min^{-1}]	0.01	Clearance rate of Cln2
k_{23L}	[mo L^{-1} min^{-1}]	$7.2*10^{14}$	Low Cln2 production rate (due to weak activation without SBF)
k_{23H}	[mo L^{-1} min^{-1}]	$4.3*10^{15}$	Regular Cln2 production rate (due to SBF activation)
k_{24}	[min^{-1}]	0.05	Clearance rate of Nrm1
k_{25L}	[mo L^{-1} min^{-1}]	$1.8*10^{13}$	Low Nrm1 production rate (due to weak activation without SBF)
k_{25H}	[mo L^{-1} min^{-1}]	$1.8*10^{15}$	Regular Nrm1 production rate (due to SBF activation)
k_{26}	[min^{-1}]	0.01	Clearance rate of Cln1
k_{27L}	[mo L^{-1} min^{-1}]	$1.8*10^{15}$	Low Cln1 production rate (due to weak activation without SBF/MBF)
k_{27H}	[mo L^{-1} min^{-1}]	$1.08*10^{16}$	Regular Cln1 production rate (due to SBF/MBF activation)
Θ_{Nrm1}	[mo]	50	Number of molecules for Nrm1 to inhibit MBF
k_{28}	[min^{-1}]	0.05	Clearance rate of Clb6
k_{29L}	[mo L^{-1} min^{-1}]	$1.26*10^{13}$	Low Clb6 production rate (due to weak activation without SBF/MBF)
k_{29H}	[mo L^{-1} min^{-1}]	$4.5*10^{15}$	Regular Clb6 production rate (due to SBF/MBF activation)
k_{34}	[min^{-1}]	0.05	Clearance rate of Clb5
k_{35L}	[mo L^{-1} min^{-1}]	$1.26*10^{13}$	Low Clb5 production rate (due to weak activation without MBF)
k_{35H}	[mo L^{-1} min^{-1}]	$6.3*10^{15}$	Regular Clb5 production rate (due to MBF activation)

Supplementary Table 7 - Input parameters for Sic1 function

Name	Unit	Value	Description
k_{30}	[min^{-1}]	0.35	Rate constant for Clb6Sic1 dissociation
k_{31}	[mo^{-1} L min^{-1}]	10^{-15}	Rate constant for Clb6Sic1 formation
k_{32}	[min^{-1}]	50	Rate constant for Clb6Sic1-P dissociation
k_{33}	[mo^{-1} L min^{-1}]	10^{-16}	Rate constant for Clb6Sic1-P formation
k_{36}	[min^{-1}]	0.35	Rate constant for Clb5Sic1 dissociation
k_{37}	[mo^{-1} L min^{-1}]	10^{-15}	Rate constant for Clb5Sic1 formation
k_{38}	[min^{-1}]	50	Rate constant for Clb5Sic1-P dissociation
k_{39}	[mo^{-1} L min^{-1}]	10^{-16}	Rate constant for Clb5Sic1-P formation
k_{40}	[mo^{-1} L min^{-1}]	$1.3*10^{-18}$	Maximum phosphorylation rate for unbounded Sic1
Θ_{40}	[mo]	150	Threshold for Cln1,2,3 in unbounded Sic1 phosphorylation
n_{40}	-	5	Hill coefficient for unbounded Sic1 phosphorylation
k_{41}	[mo^{-1} L min^{-1}]	$1.7*10^{-16}$	Maximum phosphorylation rate for Clb6Sic1
Θ_{41}	[mo]	150	Threshold for Cln1,2,3 in Clb6Sic1 phosphorylation
n_{41}	-	5	Hill coefficient for Clb6Sic1 phosphorylation
k_{42}	[mo^{-1} L min^{-1}]	$1.7*10^{-16}$	Maximum phosphorylation rate for Clb5Sic1
Θ_{42}	[mo]	150	Threshold for Cln1,2,3 in Clb5Sic1 phosphorylation
n_{42}	-	5	Hill coefficient for Clb5Sic1 phosphorylation
k_{43}	[min^{-1}]	0.7	Diffusion rate of Sic1p out of the nucleus
$Sic1_{tot}$	[mo]	300	Total amount of Sic1

Supplementary Table 8 - Input parameters for α-factor-induced cell cycle arrest

Name	Unit	Value	Description
M_0	[mo^{-1} L min^{-1}]	$1.24*10^{17}$	Maximal Far1 production rate per volume unit, due to the α-factor
M_1	min^{-1}	12.4	Maximal phosphorylation rate for Far1, due to the α-factor
μ_0	nM	58	α-factor concentration according to which Far1 production rate is half of its maximum value
μ_1	nM	58	α-factor concentration according to which Far1 phosphorylation rate is half of its maximum value
d_2	min^{-1}	0.01	Cln1,2-independent Far1-P degradation
d_3	min^{-1}	1.0	Far1-P dephosphorylation coefficient
d_4	[mo^{-1} L min^{-1}]	10^{-15}	Cln1,2-dependent Far1-P degradation
d_5	[mo^{-1} L min^{-1}]	$4.0*10^{-16}$	Far1-P-dependent Cln1,2 degradation

Supplementary Table 9 - Growth model parameters for single cells

Name	Unit	Value	Figure	Description
$P(0)$	[aa]	$1.2*10^{10}$	1B	Initial protein content
$P(0)$	[aa]	$2.0*10^{10}$	1C-F, 2J-K, S.3, S.5, S.8	Initial protein content
$R(0)$	[rib]	$1.61*10^{5}$	1B	Initial number of ribosomes
$R(0)$	[rib]	$2.68*10^{5}$	1C-F, 2J-K, S.3, S.5, S.8	Initial number of ribosomes
ρ	[rib aa^{-1}]	$1.34*10^{-5}$	1B-F, 2J-K, S.3, S.5, S.8-9	Ribosome-over-protein ratio
K_2	[aa rib^{-1} min^{-1}]	558	1B-F, 2J-K, S.3, S.5, S.8-9	Protein production rate per ribosomes

Supplementary Table 10 - Model parameters for simulations of populations of cells

Name	Unit	Value	Figure	Description
<*P*(0)>	[aa]	$1.6*10^{10}$	1G-I, S.6	Initial protein content
<*P*(0)>	[aa]	$2.0*10^{10}$	2C, 2F, 2H, 3A(m),3C-D, S.7, S.19	Initial protein content
<*P*(0)>	[aa]	$1.2*10^{10}$	3A(xs),3H	Initial protein content
<*P*(0)>	[aa]	$1.8*10^{10}$	3G	Initial protein content (WT)
<*P*(0)>	[aa]	$2.1*10^{10}$	3G	Initial protein content (extra-SBF)
<*P*(0)>	[aa]	$1.75*10^{10}$	4A-B	Initial protein content
<*P*(0)>	[aa]	$1.75*10^{10}$	4D	Initial protein content (WT)
<*P*(0)>	[aa]	$2.75*10^{10}$	4D	Initial protein content (*cln3Δ*)
<*P*(0)>	[aa]	$2.6*10^{10}$	4E	Initial protein content (*ydj1Δ*-a)
<*P*(0)>	[aa]	$1.6*10^{10}$	4E	Initial protein content (*ydj1Δ*-b)
<*P*(0)>	[aa]	$1.55*10^{10}$	6C, 6E-J	Initial protein content (WT)
<*P*(0)>	[aa]	$1.18*10^{10}$	6C, 6E-J	Initial protein content (*whi5Δ*)
<*P*(0)>	[aa]	$1.24*10^{10}$	6C, 6E-J	Initial protein content (whi5^{4E})
<*R*(0)>	[rib]	$2.15*10^{5}$	1G-I, S.6	Initial number of ribosomes
<*R*(0)>	[rib]	$2.7*10^{5}$	2C, 2F, 2H, 3A(m),3C-D, S.7, S.19	Initial number of ribosomes
<*R*(0)>	[rib]	$1.61*10^{5}$	3A(xs),3H	Initial number of ribosomes
<*R*(0)>	[rib]	$2.4*10^{5}$	3G	Initial number of ribosomes (WT)
<*R*(0)>	[rib]	$2.8*10^{5}$	3G	Initial number of ribosomes (extra-SBF)
<*R*(0)>	[rib]	$2.31*10^{5}$	4A-B	Initial number of ribosomes
<*R*(0)>	[rib]	$2.31*10^{5}$	4D	Initial number of ribosomes (WT)
<*R*(0)>	[rib]	$3.7*10^{5}$	4D	Initial number of ribosomes (*cln3Δ*)
<*R*(0)>	[rib]	$2.6*10^{5}$	4E	Initial number of ribosomes (*ydj1Δ*-a)
<*R*(0)>	[rib]	$2.1*10^{5}$	4E	Initial number of ribosomes (*ydj1Δ*-b)
<*R*(0)>	[rib]	$2.1*10^{5}$	6C, 6E-J	Initial number of ribosomes (WT)
<*R*(0)>	[rib]	$1.6*10^{5}$	6C, 6E-J	Initial number of ribosomes (*whi5Δ*)
<*R*(0)>	[rib]	$1.7*10^{5}$	6C, 6E-J	Initial number of ribosomes (whi5^{4E})
CV_{P0}, CV_{R0}	-	15%	1G-I, 2C, 2F, 2H, S.6-7	CV for *P*(0) and *R*(0)
CV_{P0}, CV_{R0}	-	20%	3A, 3C-D	CV for P(0) and R(0)
CV_{P0}, CV_{R0}	-	40%	3G	CV for P(0) and R(0)
CV_{P0}, CV_{R0}	-	22.5%	4A-B	CV for P(0) and R(0)
CV_{P0}, CV_{R0}	-	22.5%	4D	CV for P(0) and R(0), WT
CV_{P0}, CV_{R0}	-	25%	4D	CV for P(0) and R(0), *cln3Δ*
CV_{P0}, CV_{R0}	-	18%	4E	CV for P(0) and R(0), *ydj1Δ*-ab
CV_{P0}, CV_{R0}	-	12%	6C, 6E-J, S.19	CV for P(0) and R(0)
<*ρ*>	[rib aa^{-1}]	$1.34*10^{-5}$	1G-I, 2C, 2F, 2H, 3A-D, 3F-H, 6C, 6E-J, S.6-7, S.19	Ribosome-over-protein ratio

$<\rho>$	[rib aa^{-1}]	$1.32*10^{-5}$	4A-B	Ribosome-over-protein ratio
$<\rho>$	[rib aa^{-1}]	$1.32*10^{-5}$	4D	Ribosome-over-protein ratio (WT)
$<\rho>$	[rib aa^{-1}]	$1.34*10^{-5}$	4D	Ribosome-over-protein ratio (*cln3Δ*)
$<\rho>$	[rib aa^{-1}]	$1.0*10^{-5}$	4E	Ribosome-over-protein ratio (*ydj1Δ*-a)
$<\rho>$	[rib aa^{-1}]	$1.32*10^{-5}$	4E	Ribosome-over-protein ratio (*ydj1Δ*-b)
$<K_2>$	[aa rib^{-1} min^{-1}]	558	1G-I, 2C, 2F, 2H, 3A-D, 3F-H, 6C, 6E-J, S.6-7, S.19	Protein production rate per ribosome
$<K_2>$	[aa rib^{-1} min^{-1}]	500	4A-B	Protein production rate per ribosome
$<K_2>$	[aa rib^{-1} min^{-1}]	500	4D	Protein production rate per ribosome (WT)
$<K_2>$	[aa rib^{-1} min^{-1}]	530	4D	Protein production rate per ribosome (*cln3Δ*)
$<K_2>$	[aa rib^{-1} min^{-1}]	340	4E	Protein production rate per ribosome (*ydj1Δ*-a)
$<K_2>$	[aa rib^{-1} min^{-1}]	500	4E	Protein production rate per ribosome (*ydj1Δ*-b)
CV_{par}	-	5%	1G-I, S.6	CV for all but k_{3M} model parameters
CV_{par}	-	12%	2C, 2F, 2H, 3A-D, 3F, 4A-B,D-E, 6C, 6E-J, S.7, S.19	CV for all but k_{3M} model parameters
N	-	200	1G-I, S.6	Number of cells
N	-	1000	2C, 2F, 2H, S.7, S.19	Number of cells
N	-	90	3A(m)	Number of average cells
N	-	20	3A(xs)	Number of extra-small cells
N	-	100	3C-D, 3G-H, 4A-B,D-E, 6C, 6E-J	Number of cells
N	-	75	4E	Number of cells (*ydj1Δ*-a)
N	-	25	4E	Number of cells (*ydj1Δ*-b)

Supplementary Note 1

Notations

The following notations are adopted to denote molecules, reactions, equations and tables.

- The model comprises three subcellular compartments: cytoplasm, nucleus and endoplasmic reticulum. If a molecule considered in the model translocates from one compartment into another, a suffix ('cyt', 'nuc', 'ER') will be adopted to identify the molecule in the proper compartment; otherwise, no suffix will be added.
- The labels for biochemical reactions are preceded by an 'R'.
- The labels for the model equations are preceded by a 'D' or a 'C' according to whether it is a *Dynamical* equation or an algebraic *Constraint*.

Supplementary Note 2

Modeling the connection of Cell Growth with the G_1/S transition

Biology. The continuous increase of cell components sustains cell proliferation and the rate of cell growth sets the duration of the cell cycle [1-3]. As reviewed in [4], cell growth affects cell cycle progression at many different levels, being the entrance into – and execution of – the G_1/S transition the more relevant ones [5-9]. Thus in order to model the G_1/S transition, we first modeled the rate of cellular growth following a line of thought previously proposed. A large part of energy and of building blocks utilized in cellular processes is used for the biosynthesis of the informational macromolecules, i.e., RNA and proteins [10,11]. The increase of both protein and ribosome level results from the balance between the rate of protein/ribosome synthesis and degradation, the latter following a first order kinetics. Each exponential growth condition is characterized by a specific '*ribosomes-over-proteins*' ratio (ρ_{SS}) [12]. The amount of Sfp1 that localizes in the nucleus following TOR-dependent phosphorylation [13] sets the Rho value. Experimental evidence supports the notion that a negative feedback [14,15] – that involves the TOR pathway [13,16,17] – reduces the ribosome biosynthesis in presence of ribosomes not engaged in protein biosynthesis.

Reconstruction. The single cell Growth is an Ordinary Differential Equation (ODE) model, taken from [12], and dealing with the ribosome (expressed as number of ribosomes/cell,

state variable R) and protein content (expressed as number of polymerized amino acids/cell, state variable P). Both ribosome and protein dynamics are described by the balance between production and degradation rates. As far as the ribosome kinetics, the production rate is zero when the 'ribosome-over-protein' ratio exceeds parameter ρ; otherwise it is proportional (coefficient K_1) to the (positive) difference ρP-R, eqs.(D.2.1)-(C.2.1). The degradation rate is linear, with time constant D_1, eq.(D.2.1). As far as the protein accumulation kinetics, the production rate is proportional to the ribosome content through their average translational efficiency K_2 and the degradation rate is linear, with time constant D_2, eq.(D.2.2).

Computational representation. According to this biological reconstruction, the growth equations are taken to be

$$\frac{dR}{dt} = K_1\left[\rho P(t) - R(t)\right]^+ - \frac{R(t)}{D_1} \qquad \text{(D.2.1)}$$

$$\left[\rho P(t) - R(t)\right]^+ = \begin{cases} \rho P(t) - R(t), & \text{if} \quad \rho P(t) - R(t) \geq 0 \\ 0, & \text{otherwise} \end{cases} \qquad \text{(C.2.1)}$$

$$\frac{dP}{dt} = K_2 R(t) - \frac{P(t)}{D_2} \qquad \text{(D.2.2)}$$

According to a feasible setting of the input parameters (see, e.g. the set reported in Supplementary Table 2), it is

$$1/D_1 << K_1 \qquad 1/D_2 << K_1 \qquad \rho*K_2 << K_1 \qquad \text{(C.2.2)}$$

so that if the following inequality is satisfied

$$\rho*K_2 - 1/D_2 > 0 \qquad \textit{(exponential growth condition)} \qquad \text{(C.2.3)}$$

both ribosomes and proteins grow can be approximated (after a transient) by the same exponential law, with an exponential growth rate λ:

$$\lambda = \rho*K_2 - 1/D_2 \qquad \text{(C.2.4)}$$

according to which the *Mass Duplication Time*, *MDT*, is computed to be:

$$MDT = \ln(2)/\lambda \qquad \text{(C.2.5)}$$

It should be noted that λ is not hard-wired in the model, but rather it is linked to the macromolecular composition and biosynthetic activity of the cells, a connection that is made possible by the appropriate choice of the measurement units for ribosome and protein content, synthesis and degradation. The use of a standard molecular concentration to express RNA and protein content would not allow visualizing this connection. A further

result coming from the growth model is that the '*ribosome-over-protein*' ratio *R/P* asymptotically converges to parameter ρ (i.e. ρ_{ss} coincides with ρ). The reader may refer to [12] for all the details.

Supplementary Note 3

The Molecular Model of the G_1/S transition

To generate an updated model of the G_1/S transition in budding yeast, we heavily amended a previously proposed model of the G_1/S transition [18] introducing major experimental findings published since then. The basic, revised model does not account for regulatory signaling events, but it can be easily extended to incorporate them, as shown by the cell fate analysis presented in the results section (see Supplementary Note 11 of this document and Fig. 2 of the main text).

Recently, Cross and collaborators proposed to divide G_1 in two periods [19]: T_1 and T_2. The end of T_1 is set by nuclear exclusion of Whi5 [20], an inhibitor of G_1/S-specific transcription, which plays the same functional role of the retinoblastoma protein in mammalian cells [21,22] and whose function will be discussed in more detail in Supplementary Note 6. At the end of period T_1, a T_2 period starts. Major molecular events taking place during T_2 include synthesis of Cln1,2 and Clb5,6 cyclins and accumulation of Cln1,2-Cdk1 active complex that promotes budding. Instead Clb5,6-Cdk1 first interacts with Sic1 to form an inactive complex. The end of the T_2 period is set when 50% of Sic1 is exported from the nucleus following phosphorylation by Cln1,2-Cdk1 and by free Clb5,6-Cdk1 [23]. The bulk of Clb5,6-Cdk1 is released to promote DNA synthesis [24].

The molecular model takes into account the following classes of events:

i. Regulation of Cln3-Cdk1 activity, i.e. Cln3 synthesis (in the cytoplasm), its subsequent retention in the endoplasmic reticulum and its transport in the nucleus by the Ydj1 chaperone as well as Cln3 inhibition by Far1.
ii. Transcriptional activation of the G_1/S regulon, first by Cln3-Cdk1 kinase activity, which includes a computationally effective description of regulon activation - including Cln1,2-mediated positive feedback - and multisite phosphorylation of Whi5.

iii. Regulation of Clb5,6-Cdk1 activity - that triggers entrance into S phase - by binding of Sic1, whose nuclear level and inhibitory activity are regulated by both Cln1,2-Cdk1 AND Clb5,6-Cdk1 phosphorylation activity.

The model - which takes into account cytoplasm ('cyt'), endoplasmic reticulum ('ER') and nucleus ('nuc') sub-cellular compartments - is a hybrid continuous/discrete-event model. The amount and sub-cellular localization of the molecular players involved in the activation of the regulon is modeled by a set of Ordinary Differential Equations (ODE). Regulon activation depends on a correct sequence of multi-site phosphorylation steps - catalyzed by Cln1,2,3-Cdk1 complexes. A discrete-event stochastic model that calculates probability distributions of the different phosphorylation states describes the phosphorylation states of DNA-bound SBF and MBF transcriptional activators, as well as of the transcriptional inhibitor Whi5.

Due to the abundance of Cdk1 [25] and to the fast dynamics of Cdk1 binding to its cyclin targets, the molecular model does not explicitly consider the binding dynamics for Cdk1 and cyclin Clns and Clbs. Therefore, whenever Clns or Clbs are found in the following, they have to be considered as already bound to Cdk1. All the equations are written in terms of molecule amounts (instead of concentrations).

Supplementary Note 4

Cln3 synthesis and nuclear transport

Biology. The most upstream cyclin acting in the control of the G_1/S transition is Cln3, a weakly expressed, highly unstable protein whose abundance rapidly responds to changes in the nutritional status: Cln3 levels are higher in fast growing cells (for instance cells growing in the presence of glucose as a carbon source) than in slow growing cells (for instance cells growing in the presence of ethanol as a carbon source)[26-32]. Since Cln3 is a very low abundance protein[33], its accurate quantification is a non-trivial task. Early studies suggested that Cln3 levels do not oscillate significantly in synchronous cultures of elutriated G_1 daughter cells: Cln3 is already present in newborn G_1 phase cells and its concentration does not increase throughout G_1 phase[26]. These early findings appeared to be confirmed by recent single cell time-lapse microscopy studies, which demonstrated that Cln3 concentration (number of molecules/volume) remains constant as cells grow during

G_1 phase[34]. This study employed hyperstable mutant versions of the cyclin lacking the phosphosites that normally regulate the degradation of endogenous Cln3[35-37]: therefore, the physiological relevance of these observations is uncertain, since Cln3 turnover is essential for accurate regulation of cell cycle[36,38,39].

In contrast to previous findings, Zapata and coworkers recently showed that Cln3 is initially absent in small newborn G_1 cells and subsequently accumulates during growth in G_1 phase, reaching a peak level before bud emergence[40]. In that study, ethanol-growing cells were synchronized by centrifugal elutriation and released in glucose-supplemented medium. Besides recovering from elutriation-related stress (a problem common to all elutriation experiments), these cells need to adapt from a nutritional condition that is known to be characterized by a low Cln3 content (ethanol-supplemented medium, pre-elutriation) to a nutritional condition that has higher Cln3 level (glucose-supplemented medium, post-elutriation). Since an ethanol-to-glucose nutritional shift-up induces an increase in Cln3 content (as previously reported in asynchronous cell population (Fig. 4 in reference [28]), it is doubtful whether these results reflect a gradual accumulation of Cln3 along the G_1 phase, as suggested by the Authors[40]. The same study also reported a peak of Cln3 accumulation in late G_1 cells released from α-factor arrest, which had not been detected in previous works[26,36].

The Whi3 gene product inhibits translation of *CLN3* mRNA [41,42], while newly synthesized Cln3 is retained in the ER and its nuclear transport is promoted by Ydj1, a type I HSP40 co-chaperone member of the DnaJ family [43] in association with Whi7 [44].

__*Reconstruction*__. In our model Cln3 synthesis is not explicitly modeled. For this reason, the role of Whi3 is omitted. As discussed above, conflicting evidences regarding the precise pattern of Cln3 protein accumulation during the G_1 phase have been reported. Our simulations show that the G_1/S transition kinetics are quite insensitive to the specific pattern of Cln3 accumulation (Supplementary Figs. 1-2), while they are dependent on average Cln3 level during G_1 (*cln3Δ* and Cln3 overexpression cases). Therefore we used the simpler hypothesis, i.e. that Cln3 concentration is constant throughout the G_1 phase. Biochemical reactions involving Cln3 in the early G_1 phase are as follows (see also Fig.1A):

$$Cln3_{cyt} \leftrightarrows Cln3_{nuc} \qquad (R.4.1)$$

$$Cln3_{cyt} \leftrightarrows Cln3_{ER} \qquad (R.4.2)$$

$$Cln3_{ER} + Ydj1_{ER} \rightarrow Cln3Ydj1_{ER} \qquad (R.4.3)$$

$$Cln3Ydj1_{ER} \rightarrow Cln3Ydj1_{nuc} \quad (R.4.4)$$

$$Cln3Ydj1_{nuc} \rightarrow Cln3_{nuc} + Ydj1_{nuc} \quad (R.4.5)$$

The following simplifying assumptions have been incorporated into the model.

i. Cln3 is produced in the cytoplasm throughout the G_1/S transition so to keep fixed the ratio of total Cln3 versus the protein content
ii. $Cln3_{cyt}$ can either translocate to the nucleus, reaction (R.4.1), or to the ER, reaction (R.4.2), the latter reaction being favored
iii. $Cln3_{ER}$ translocation into the cytoplasm does not depend on $Cln3_{ER}$ amount and it occurs with a very high cell-to-cell variability
iv. Binding of chaperone Ydj1 to Cln3 takes place in the ER and is unidirectional, reaction (R.4.3)
v. Chaperone Ydj1 concentration is assumed to be in large excess, i.e. the binding of $Cln3_{ER}$ and Ydj1 does not depend explicitly on Ydj1
vi. Complex Cln3Ydj1 directly diffuses into the nucleus from the ER, through an irreversible, reaction (R.4.4)
vii. Dissociation of $Cln3Ydj1_{nuc}$ is irreversible, reaction (R.4.5)

Computational representation. Total Cln3 increases with total protein, newly formed Cln3 being cytoplasmic:

$$Cln3_{tot}(t) = k_0\, P(t) = Cln3_{nuc}(t) + Cln3_{cyt}(t) + Cln3_{ER}(t) + Cln3Ydj1_{ER}(t) + Cln3Ydj1_{nuc}(t) + Cln3Far1(t) \quad (C.4.1)$$

Translocation from cytoplasm into ER is reversible, last term in eq.(D.4.1). The diffusion rate from the ER into the cytoplasm is independent of the Cln3 amount for values greater than a minimum threshold Θ_3. Below such a threshold the diffusion rate is proportional to $Cln3_{ER}$. In summary:

$$k_3(Cln3_{ER}) = k_{3M}, \qquad for \quad Cln3_{ER} > \Theta_3$$

$$k_3(Cln3_{ER}) = (k_{3M}/\Theta_3)Cln3_{ER}, \qquad for \quad Cln3_{ER} \leq \Theta_3 \quad (C.4.2)$$

Cln3 translocation into the nucleus follows two routes. From one hand, a chaperone-independent, reversible reaction (R.4.1) yields a net nuclear diffusion (last two terms in eq.(D.5.1)). Such diffusion yields a modest contribution to Cln3 nuclear localization in wild type cells, instead of the chaperone-mediated nuclear transport. It is a two-step process that entails non-reversible formation of the Cln3Ydj1 complex – reaction (R.4.3), that effectively depletes $Cln3_{ER}$ – followed by non-reversible Cln3Ydj1 diffusion into the nucleus, reaction (R.4.4). Cln3Ydj1 formation (first and last terms in eqs.(D.4.1) and

(D.4.2) respectively) is modeled by a function of only $Cln3_{ER}$, since Ydj1 is taken to be in large excess (parameter k_1 accounting for Ydj1).

The diffusion of Cln3Ydj1 into the nucleus is ruled by a sharp Hill function (first terms in eqs.(D.4.2-3), with a high Hill coefficient n_4), i.e. until $Cln3Ydj1_{ER}$ is close enough to the threshold Θ_4 the rate of diffusion into the nucleus is approximately fixed to the low value k_{4L}; on the other hand, the rate of diffusion may be approximated by the high value k_{4H} when cytoplasmic Cln3Ydj1 is far beyond the threshold Θ_4. At the very beginning of the cycle, wild type cycling cells are assumed to have an initial amount of $Cln3_{ER}$ high enough to exceed such a threshold. Therefore such a machinery plays an effective role especially in extra-small or starved cells.

Once in the nucleus, complex Cln3Ydj1 dissociates irreversibly, reaction (R.4.5), thus releasing Cln3 (last and first terms in eqs.(D.4.3) and (D.5.1) respectively). Below are reported the dynamics concerning $Cln3_{ER}$ and complex Cln3Ydj1:

$$\frac{dCln3_{\mathrm{ER}}}{dt} = -k_1 Cln3_{\mathrm{ER}} + k_2 Cln3_{\mathrm{cyt}} - k_3 \left(Cln3_{\mathrm{ER}}\right) \tag{D.4.1}$$

$$\frac{dCln3Ydj1_{\mathrm{ER}}}{dt} = -\left(k_{4\mathrm{L}} + \left(k_{4\mathrm{H}} - k_{4\mathrm{L}}\right)\frac{\left(\frac{Cln3Ydj1_{\mathrm{ER}}}{\Theta_4}\right)^{n_4}}{1+\left(\frac{Cln3Ydj1_{\mathrm{ER}}}{\Theta_4}\right)^{n_4}}\right) Cln3Ydj1_{\mathrm{ER}} + k_1 Cln3_{\mathrm{ER}} \tag{D.4.2}$$

$$\frac{dCln3Ydj1_{\mathrm{nuc}}}{dt} = \left(k_{4\mathrm{L}} + \left(k_{4\mathrm{H}} - k_{4\mathrm{L}}\right)\frac{\left(\frac{Cln3Ydj1_{\mathrm{ER}}}{\Theta_4}\right)^{n_4}}{1+\left(\frac{Cln3Ydj1_{\mathrm{ER}}}{\Theta_4}\right)^{n_4}}\right) Cln3Ydj1_{\mathrm{ER}} - k_5 Cln3Ydj1_{\mathrm{nuc}} \tag{D.4.3}$$

Supplementary Note 5

Inhibition of nuclear Cln3Cdk1 kinase activity by Far1

Biology. The protein-protein interaction (PPI) network that includes proteins involved in cell growth and cell cycle is composed by more than 900 proteins [4,45]. About 20% of the arcs within the Growth & Cycle PPI network connect proteins of the *Cell Cycle* sub-network with proteins of the *Cell Growth* sub-network, indicating a strong interconnection between the processes of cell growth and cell cycle [4], as envisioned by earlier work [46]. The more relevant connection is given by the control that growth has on the G_1/S transition [3,5].

In budding yeast a cell sizer control appears to be operative over the entrance into S phase and budding [19]. Gene dosage data strongly support a promoting role of Cln3 in the

sizer mechanism [39,47,48]. After mitosis, a given amount of nuclear Far1 - an inhibitor of all Cln-Cdk complexes - is endowed to each nucleus [49]. Experimental findings support the notion that the cyclin-dependent kinase inhibitor Far1 is involved in the cell sizer mechanism in cycling cells - as well as it is involved in the mating program [50] - by inhibiting Cln3Cdk1 in early G_1 [28,51]. Eventually, continuous Cln3 production and nuclear transport (mainly due to the chaperone Ydj1) allows to overcome Far1 inhibition, which is made irreversible by Far1 degradation primed by the increasing Cln-Cdk1 activity. A mathematical model of the G_1 to S transition, that considers the overcoming of a Cln3/Far1 threshold as the beginning of G_1 phase in cycling cells, has been reported in [18].

Reconstruction. The Cln3Far1 complex formation/dissociation occurs in the nucleus according to standard mass-action law.

$$\mathrm{Cln3_{nuc}} + \mathrm{Far1} \leftrightarrows \mathrm{Cln3Far1} \qquad \text{(R.5.1)}$$

Synthesis and transport of Cln3 have been described in Supplementary Note 4, reactions (R.4.1-5). Far1 degradation is controlled by the interplay between free nuclear Cln3 and the inactive complex Cln3Far1, and is formally triggered when active nuclear Cln3 exceeds the inhibited complex Cln3Far1.

Computational representation. By denoting with k_6, k_7 the rate constants (the former for complex Cln3Far1 unbinding, the latter for Cln3Far1 binding) for reaction (R.5.1), below follow the dynamical equations for nuclear Cln3 and Far1:

$$\frac{dCln3_{\mathrm{nuc}}}{dt} = k_5 Cln3Ydj1_{\mathrm{nuc}}(t) - \frac{k_7}{V_{\mathrm{nuc}}(t)} Cln3_{\mathrm{nuc}}(t) \cdot Far1(t) + k_6 Cln3Far1(t) - k_9 Cln3_{\mathrm{nuc}}(t) + k_8 Cln3_{\mathrm{cyt}}(t) \qquad \text{(D.5.1)}$$

$$\frac{dFar1}{dt} = -\frac{k_7}{V_{\mathrm{nuc}}(t)} Cln3_{\mathrm{nuc}}(t) \cdot Far1(t) + k_6 Cln3Far1(t) - k_{10} \frac{\left(\frac{Cln3_{\mathrm{nuc}}(t)}{Cln3Far1(t)}\right)^{n_{10}}}{1 + \left(\frac{Cln3_{\mathrm{nuc}}(t)}{Cln3Far1(t)}\right)^{n_{10}}} Far1(t) \qquad \text{(D.5.2)}$$

$$\frac{dCln3Far1}{dt} = \frac{k_7}{V_{\mathrm{nuc}}(t)} Cln3_{\mathrm{nuc}}(t) \cdot Far1(t) - k_6 Cln3Far1(t) \qquad \text{(D.5.3)}$$

Notice that the choice to write the equations in terms of number of molecules, instead of concentrations, introduces the requirement of volume determination for the second order terms (accounting for the Cln3/Far1 binding). In the present model the cell volume is constrained to be proportional to the overall protein content and the nuclear volume is constrained to be a proper fraction of the overall cell volume [52]: therefore, both cell and

nuclear volume increase according to the same exponential growth rate of the protein content:

$$V_{cell}(t) = P(t) / H, \qquad V_{nuc}(t) = h*V_{cell}(t) \qquad (C.5.1)$$

with parameter H being the 'protein-over-cell volume' ratio and parameter h being the 'nucleus-over-cell volume' ratio.

Far1 degradation, last term in eq.(D.5.2), increases to a very high value (parameter k_{10}) as soon as free nuclear Cln3 exceeds its inhibited form Cln3Far1: to this aim a Hill function has been used to implement such a saturating function.

Supplementary Note 6

Multi-site phosphorylation of Whi5 by Cln1,2,3-Cdk1

Biology. Whi5 is a highly disordered, fungal-specific transcriptional inhibitor with analogous function, but no sequence homology - to the retinoblastoma protein in higher eukaryotes. Genetic data outlined in Results and fully discussed in a recent publication [22] indicate that the transcriptional inhibitory activity of Whi5 is controlled through multisite phosphorylation mediated by Cln1-3-Cdk1. Among the twelve total Cdk1 phospho-acceptor sites present in Whi5, four specific sites clustered in an evolutionary conserved C terminal part of the protein are functionally relevant when four specific Cdk1 phospho-acceptor sites are concurrently mutated in Swi6 [53]. Phosphorylation of the 4 phospho-acceptor sites in Whi5 alters intra-molecular interaction, ultimately resulting in Whi5 dissociation from the Swi6-Swi4 (SBF) complex, freeing it to activate G_1/S-specific transcription [22]. *WHI5* overexpression yields an increase in cell size and an extension of G_1 phase, whereas its deletion yields cells that are smaller than wild type [54]. The Whi5 domain from residues 181 to 205, called the "GTB motif" (**G**$_1$/S **T**ranscription factor **B**inding), binds the carboxyl terminal of Swi6 and is required for transcriptional repression [55]. Recent data confirm that Swi4 and Mbp1 interact with Swi6 but not with each other and support the notion that the binding of Whi5 is limited to SBF, while the repressor Nrm1, which is a product of the G_1/S regulon, interacts with MBF [56], repressing transcription of the G_1/S regulon as cells progress through S phase [57].

The activation of both SBF and MBF requires Cdk1-dependent phosphorylation, but the molecular details differ. Although Swi6, the co-activator that is common to both SBF and MBF, contains several Cdk1 phospho-acceptor sites that may contribute to the activation

of both SBF and MBF, SBF regulation takes place chiefly through the phosphorylation of the transcriptional repressor Whi5, whose twelve Cdk1 phospho-acceptor sites are mostly occupied *in vivo* in asynchronously growing cells [53]. Whi5 phosphorylation, first by Cln3-Cdk1 [21,58] and later by Cln1,2-Cdk1 [59], promotes the dissociation of Whi5 from SBF bound to its cognate promoters, thus activating the transcription of the G_1/S regulon [60]. A positive feedback loop that relies on *CLN1,2* transcription [61] ultimately commits yeast cells to the cell cycle while phosphorylated Whi5 is exported from the nucleus [21,62].

Mutational analysis has detected four specific sites required for Whi5 function, easily evidenced *in vivo* when the four Cdk1 phospho-acceptor sites of Swi6 are all mutated to alanine. When the four critical phosphosites of Whi5 are completely phosphorylated, Whi5 is released from Swi6 - likely following a conformational change - so that transcription may start from the SBF-bound promoters. Dissociation of the SBF-Whi5 complex may also derive from the full phosphorylation of the critical Swi6 phosphosites [22,53]. Thus, conformational changes that disrupt the Whi5-Swi6 interaction and eventually cause the activation of the SBF branch of the G_1/S regulon can be achieved through phosphorylation of a precise pool of Cdk1 sites belonging to a trans-modular domain formed by the disordered motifs of both Whi5 and Swi6. In order to investigate the role of the specific configuration of functional to total Cdk1 phosphorylation sites in Whi5 (4/12 in the wild type), other configurations have been considered, by varying the number of functional sites, as schematically outlined in the scheme of Fig. 6.1.

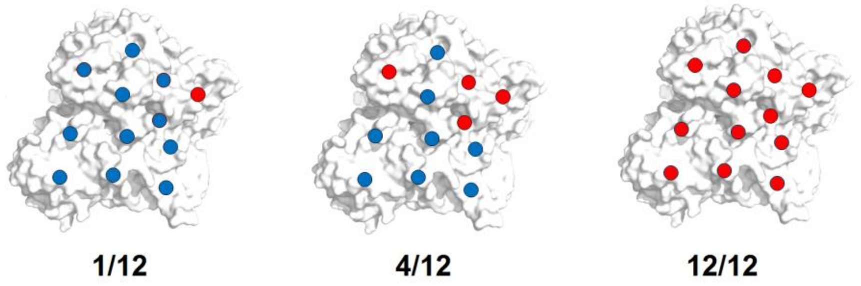

Figure 6.1 Functional (red) vs decoy (blue) distribution of the Whi5 phosphorylation sites.

<u>*Reconstruction.*</u> A multi-site phosphorylation model has been considered to control how transcription factors SBF and MBF are activated to regulate the G_1/S regulon, that includes a total of 362 genes [60]. Our model accounts for the subset of 235 whose transcriptional regulation is better understood. These genes can be divided in 3 major classes according to the SBF/MBF binding [60]. Class A consists of N_A = 136 genes whose transcription is regulated by the transcription factor SBF. Class B consists of N_B = 63 genes and is

regulated by the transcription factor MBF. Class C consists of N_C = 36 genes and is regulated by the OR logic function of MBF and SBF. Class A and C genes will also be called SBF-affine genes, while class B and C genes will also be called MBF-affine genes.

As far as the multi-site phosphorylation, there are three distinct phosphorylation sites: one concerns Swi6 and involves both SBF and MBF, the other two concern Whi5 and involve only SBF, target of Whi5 action. The site involving Swi6 provides a pattern of n_s = 4 phosphorylations, at the end of which SBF is activated. The two Whi5 sites consist of n_{w1} = 4 functional and n_{w2} = 8 decoy phosphorylation sites: the functional sites are effectively responsible for the Whi5 inactivation, while the decoys play a role of competitor in terms of kinase demand. Whi5 is inactivated (and, then, released from the binding site) as soon as the pattern of n_{w1} functional phosphorylations is completed. All phosphorylations are catalyzed by the same pool of cyclins: Cln3 is the main and upstream kinase, starting to work since the beginning of Timer T_1, as soon as it diffuses into the nucleus and gets rid of its inhibitor Far1; Cln1 and Cln2 act in a positive feedback loop with Cln3 soon after their expression. The overall contribution of G_1 cyclins to phosphorylation is given by the following relation:

$$f_{20}(Cln_{1,2,3}) = Cln3_{\text{nuc}} + \frac{1}{2}(Cln1 + Cln2) \tag{C.6.1}$$

The cyclin-driven phosphorylation coefficient α_k defined in (C.6.2) accounts also for the following facts:

- saturation in the cyclins phosphorylation effectiveness as soon as their combination in (C.6.1) approaches and exceeds a given accumulation level Θ_{20}; thus cyclins enter α_k by means of a smooth Hill function (low Hill coefficient n_{20});
- reduction in the cyclins phosphorylation effectiveness by means of a factor proportional to the overall number of free phosphorylation sites (N_{free}, in (C.6.2)). This fact is substantially ruled by the decoy Whi5 phosphorylation sites. The computation of N_{free} will be discussed in the next section.

$$\alpha_k(t) = \frac{k_{20}}{V_{\text{nuc}}(t)} \cdot \frac{X(t)^{n_{20}}}{\Theta_{20}^{n_{20}} + X(t)^{n_{20}}} \cdot \frac{1}{N_{20} + N_{\text{free}}(t)}, \quad X(t) = f_{20}\big(Cln_{1,2,3}(t)\big) \tag{C.6.2}$$

Notice that α_k is also divided by the nuclear volume since the phosphorylation contribute to the dynamics occurs as a second order term (there is the product of substrate and kinase).

<u>*Computational representation.*</u> In our phosphorylation model the state variables are given by the probabilities an SBF/MBF-affine gene has of being free of (or bound to) the corresponding transcription factor and, in this latter case, with how many phosphorylations

out of a maximum number. Thus we can write two vectors of probabilities: one for the MBF-affine genes (denoted P^d, where the superscript *d* refers to the *dimer* Swi6Mbp1) and the other for the SBF-affine genes (denoted P^t, where the superscript *t* refers to the *trimer* Swi6Swi4Whi5). The probability vector $P^d(t)$ has $n_s + 2$ components, namely $P^d_i(t)$, $i = -1, 0, 1, \ldots, n_s$, with $P^d_{-1}(t)$ being the probability that, at time *t*, an MBF-affine gene is free of Swi6/Mbp1, and the other components $P^d_i(t)$ being the probabilities that, at time *t*, an MBF-affine gene is bound to Swi6-P^i/Mbp1, with Swi6 *i*-times phosphorylated. As far as the SBF-affine genes, the components of the probability vector $P^t(t)$ will be addressed using three subscripts (j_1,j_2,j_3). For $j_1 = 0, \ldots, n_s$, $j_2 = 0, \ldots, n_{w1}$, $j_3 = 0, \ldots, n_{w2}$, $P^t_{j1,j2,j3}(t)$ denotes the probability that, at time *t*, an SBF-affine gene is bound to Swi6-P^{j1}/Swi4/Whi5-$P^{j2,j3}$ where (j_1,j_2,j_3) are the number of phosphorylations: j_1 of Swi6, j_2 of the functional sites of Whi5 and j_3 of the decoy sites of Whi5. Whi5 detaches only when the SBF-affine gene reaches one of the states $\{(n_s,j_2,j_3), j_2 = 0, \ldots, n_{w1}, j_3 = 0, \ldots, n_{w2}\}$ or $\{(j_1,n_{w1},j_3), j_1 = 0, \ldots, n_s, j_3 = 0, \ldots, n_{w2}\}$. If $(j_1, j_2, j_3) = (-1, -1, -1)$, then $P^t_{-1,-1,-1}(t)$ is the probability that at time *t* an SBF-affine gene is not bound to the transcription factor. For $(j_1, j_2, j_3) = (j_1, -1, -1)$, with $j_1 \neq -1$, we have the probability that an SBF-affine gene is bound to the transcription factor with the inhibitor Whi5 unbound (i.e. Swi6-P^{j1}/Swi4). Notice that not all the triples $(j_1, j_2, j_3) \in \{-1, 0, \ldots, n_s\} \times \{-1, 0, \ldots, n_{w1}\} \times \{-1, 0, \ldots, n_{w2}\}$ are allowed. The *impossible* states are listed below:

- $j_1 = -1$ AND $(j_2, j_3) \neq (-1, -1)$: Whi5 cannot be bound (with any phosphorylation combination) if SBF is not bound
- $[j_2 = -1$ AND $j_3 \neq -1]$ OR $[j_2 \neq -1$ AND $j_3 = -1]$: the two phosphorylation regions of Whi5 must be both bound or both unbound.

Probabilities P^d and P^t provide an estimate of the SBF/MBF-affine gene percentage distribution. Therefore, by multiplying P^d by $N_B + N_C$, i.e. the number of MBF-affine genes, we have an estimate of MBF-affine gene distribution, as well as by multiplying P^t by $N_A + N_C$, i.e. the number of SBF-affine genes, we have an estimate of the SBF-affine gene distribution.

Transcription factors binding as well as phosphorylation processes are properly exploited in the dynamical equations providing $P^d_i(t)$ and $P^t_{j1,j2,j3}(t)$, and will be described in details in the following subsection. See [63-65] for recent relevant references on modeling of stochastic processes.

Supplementary Note 7

Transcriptional activation of the G_1/S regulon

<u>*Biology*</u>. In *S.cerevisiae*, commitment to the mitotic cell cycle takes place at START [60], a regulatory area in the G_1 phase of the cell cycle where a yeast cell integrates intracellular and extracellular signals. It has been recently proposed that passage through START corresponds to the activation of a G_1 cyclin positive feedback loop that involves Cln3 – the most upstream acting G_1 (Cln type) cyclin - and the downstream Cln1,2 cyclins [60]. A major Cln3 target at START is Whi5, a transcriptional inhibitor whose inactivation starts expression of the so-called G_1/S regulon, i.e. the group of genes whose expression is coordinately up-regulated in cells entering S phase. The ensuing expression of the *CLN1* and *CLN2* genes driven by Cln3-mediated phosphorylation completes the positive feedback loop through further inactivation and nuclear exclusion of Whi5 and full activation of two transcription factors sharing the same Swi6 regulatory subumit: SBF (Swi4-Swi6) that binds to - and is inhibited by Whi5 - and MBF (Mbp1-Swi6). Mutational analysis has defined that SBF activation requires phosphorylation of specific residues either in Whi5 OR in the Swi6 subunit of SBF itself [53]. Concurrent MBF also requires Cdk1 activity, likely through phosphorylation of the shared component Swi6 [66]. At later times, MBF activity is down-regulated by the product of the *NRM1*, encoding a protein related to Whi5 [57,67].

In early G_1 cells Whi5 binds to SBF, inhibiting transcription. Whi5 carries 12 Cdk1 phospho-acceptor sites, all of which have been found to phosphorylate *in vivo*. Genetic evidence suggests that four of these sites are functionally relevant and that phosphorylation of *either* these specific Whi5 sites *or* four sites on the Swi6 subunit of SBF is required to trigger transcription of the SBF-affine genes of the G_1/S regulon [53]. In our model, Cln-Cdk1-mediated phosphorylation of the functional four sites of Whi5 is the central device supporting coherent initiation of G_1/S regulon transcription, conferring robust timing to cell cycle events, in keeping with a pattern previously described for another multisite protein phosphorylation [24,68]. Cdk1-dependent activation of MBF involves phosphorylation of four Swi6 sites as well. Since dual-regulated targets are activated by the earliest active transcription factor, transcriptional activation of the dual-target genes functions as a logical OR gate, a single active transcription factor - either SBF or MBF - being sufficient to activate their transcription. Notably, *CLN1* is a dual regulated gene, so that - within experimental error - it is the earliest activated gene under a variety of

experimental conditions. *CLN2*, that is an SBF-only regulated gene, is activated a few minutes after *CLN1* [60].

CLB5, whose product promotes DNA synthesis [69], is activated about 10 minutes later than *CLN1*, while *CLB6*, a gene whose product has redundant functions with those of Clb5 is transcribed even later. *NMR1*, the gene encoding the negative feedback co-repressor which down-regulates transcription of MBF regulated genes [57], is transcribed about 15 min later than *CLN1*, so to allow enough time to the accumulation of Cln1 and Cln2 to sustain the positive feedback on G_1/S regulon transcription prior to Nrm1 dependent transcriptional inactivation [60].

<u>Reconstruction.</u> The binding of Swi6 and Swi4 to form SBF = Swi6Swi4 follows the usual mass action laws of complex formation, as well as the binding of Swi6 and Mbp1 to form MBF = Swi6Mbp1

$$\text{Swi6} + \text{Swi4} \leftrightarrows \text{Swi6Swi4} \qquad \text{(R.7.1)}$$

$$\text{Swi6} + \text{Mbp1} \leftrightarrows \text{Swi6Mbp1} \qquad \text{(R.7.2)}$$

Moreover, complex SBF is made inactive by the S-phase inhibitor Whi5, by forming the trimer Swi6Swi4Whi5:

$$\text{Swi6Swi4} + \text{Whi5} \leftrightarrows \text{Swi6Swi4Whi5} \qquad \text{(R.7.3)}$$

A newborn cell has a large amount of nuclear Whi5 so that most SBF is inhibited in the early G_1 phase.

The activation of the G_1/S regulon is simulated by means of a stochastic model, that obeys the following basic rules:

i) the binding of complexes SBF and SBFWhi5 takes place on class A and class C genes; MBF binds to class B and class C genes; the binding of SBF, SBFWhi5 and MBF are independent events

ii) class A genes are activated when SBF is bound to the promoter region free of its inhibitor Whi5: either it binds directly, or it first binds inhibited by Whi5 and then gets rid of the inhibitor at the end of a Cln-Cdk1-mediated phosphorylation process

iii) class B genes are activated when MBF bound to the promoter region is activated at the end of a Cln-Cdk1-mediated phosphorylation process

iv) class C genes are activated by the OR logic function of items ii) and iii)

v) the Swi6 subunit of SBF and MBF has n_s (n_s = 4 in the standard data set for simulation of wild type cells) phosphorylation sites in Swi6; Whi5 has two classes of phosphorylation sites whose phosphorylation has different physiological

outcomes: the first class encompasses n_{w1} (n_{w1} = 4 in the standard data set for simulation of wild type cells) functional phosphorylation sites. These sites are physiologically relevant and lead to dissociation of the SBFWhi5 complex and transcriptional activation of the bound cognate genes; the second class encompasses n_{w2} (n_{w2} = 8 in the standard data set for simulation of wild type cells) decoy phosphorylation sites. Phosphorylation of these sites does not lead to dissociation of the SBFWhi5 complex and transcriptional activation of the bound cognate genes. It works by engaging Cdk1 phosphorylation activity.

vi) phosphorylations occur only when SBF, SBFWhi5 or MBF are bound to the promoter region of the corresponding genes

vii) all the phosphorylations are catalyzed by the same Cln1,2,3-driven coefficient, defined in (C.6.2)

viii) SBF-affine genes bound to SBFWhi5 are activated when Whi5 detaches from SBF. The detachment occurs (at a given rate) only after full phosphorylation of Swi6 or of the functional sites of Whi5. These state transitions are below summarized:

$(n_s,j_2,j_3) \rightarrow (n_s,-1,-1)$, $\quad j_2 = 0, \ldots, n_{w1}$, $\quad j_3 = 0, \ldots, n_{w2}$

$(j_1,n_{w1},j_3) \rightarrow (j_1,-1,-1)$, $\quad j_1 = 0, \ldots, n_s-1$, $\quad j_3 = 0, \ldots, n_{w2}$

Computational representation. The following equations refer to the SBF and MBF dynamics:

$$\frac{dSwi6Swi4}{dt} = \frac{k_{12}}{V_{\text{nuc}}(t)} Swi6(t) \cdot Swi4(t) - k_{11} Swi6Swi4(t) - \frac{k_{14}}{V_{\text{nuc}}(t)} Swi6Sw4(t) \cdot Whi5_{\text{nuc}}(t) + k_{13} Swi6Swi4Whi5(t) - \alpha_{\text{b}}(t)(N_A + N_C) P^{\text{t}}_{-1,-1,-1}(t) \tag{D.7.1}$$

$$\frac{dSwi6Swi4Whi5}{dt} = \frac{k_{14}}{V_{\text{nuc}}(t)} Swi6Sw4(t) \cdot Whi5_{\text{nuc}}(t) - k_{13} Swi6Swi4Whi5(t) - \alpha_{\text{t}}(t)(N_{\text{A}} + N_{\text{C}}) P^{\text{t}}_{-1,-1,-1}(t) \tag{D.7.2}$$

$$\frac{dSwi6Mbp1}{dt} = \frac{k_{15}}{V_{\text{nuc}}(t)} Swi6(t) \cdot Mbp1(t) - k_{16} Swi6Mbp1(t) - \alpha_{\text{d}}(t)(N_{\text{B}} + N_{\text{C}}) P^{\text{d}}_{-1}(t) \tag{D.7.3}$$

with k_{11}, k_{12} rate constants for reaction (R.7.1), k_{13}, k_{14} rate constants for reaction (R.7.3), k_{15}, k_{16} rate constants for reaction (R.7.2). The last terms in (D.7.1-3) involve the stochastic part of the model taking into account the probability distribution of the genes

bound to the corresponding transcription factors. To this aim, probability $P^{d}_{-1}(t)$ is exploited in eq.(D.7.3) to model the clearance rate of complex Swi6Mbp1, due the binding of MBF to the MBF-affine gene population, as a rate proportional to the average number of unbound MBF-affine genes (i.e. $(N_B + N_C)P^{d}_{-1}(t)$) and to the ligand concentration itself, by means of coefficient $\alpha_d(t)$ given by:

$$\alpha_{\mathrm{d}}(t) = \frac{k_{17}}{V_{\mathrm{nuc}}(t)} Swi6Mbp1(t) \tag{C.7.1}$$

A similar approach is followed for the SBF-affine genes. Therefore, vector $P^{t}_{-1,-1,-1}(t)$ is exploited to model the clearance rates of complexes Swi6Swi4 (eq.(D.7.1)) and Swi6Swi4Whi5 (eq.(D.7.2)), due the binding of SBF and SBFWhi5 to the SBF-affine gene population, as rates proportional to the average number of unbound SBF-affine genes (i.e. $(N_A + N_C)P^{t}_{-1,-1,-1}(t)$) and to the ligand concentration itself, by means of coefficient $\alpha_b(t)$ and $\alpha_t(t)$:

$$\alpha_{\mathrm{b}}(t) = \frac{k_{18}}{V_{\mathrm{nuc}}(t)} Swi6Swi4(t) \qquad \alpha_{\mathrm{t}}(t) = \frac{k_{19}}{V_{\mathrm{nuc}}(t)} Swi6Swi4Whi5(t) \tag{C.7.2}$$

Transcription factors binding as well as phosphorylation processes are properly exploited in the dynamical equations for $P^{d}_{i}(t)$ and $P^{t}_{j1,j2,j3}(t)$. Let us consider the dynamics of the probability distribution P^{d} of the MBF-affine genes. To this end, consider the following graph:

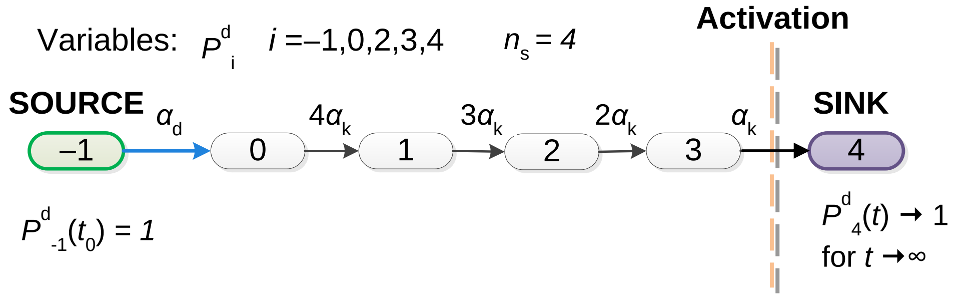

Figure 7.1 Graph representation of the transitions between the states of MBF-affine genes. The blue arrow represents the binding of the promoter to Swi6-P^0/Mbp, with rate α_d. The black arrows represent state transitions due to phospohorylation of free sites of Swi6-P^i, where the transition rate is the product of the phosphorylation rate of a single site, multiplied by the number of free sites. The gene activation happens when the last free site of Swi6 is phosphorylated.

Fig. 7.1 reports the graphic representations of the transitions among different states of MBF-affine genes in the form of a simple oriented graph (a chain). The source node (–1) represents the genes whose promoters are free of the transcription factor, and $P^{d}_{-1}(t)$ is the probability of an MBF-affine gene of being in such a state at time t. The first arc represents

the binding of the (unphosphorylated) Swi6Mbp1 to the promoter, and thus the second state represents all genes whose promoters are bound to unphosphorylated Swi6Mbp1 (probability $P^{d}_{0}(t)$). All the subsequent arcs represent phosphorylation processes, and the subsequent nodes represent increasing phosphorylation levels. The last state is a *sink*-state that represents activated genes with Swi6 fully phosphorylated. The last transition coincides with the gene activation. Thus, $P^{d}_{n_s}(t)$ is the probability of an MBF-affine gene of being activated at time t. According to the graph of Fig. 7.1, the following equations describe the dynamics of $P^{d}_{i}(t)$, i = -1, 0, …, n_s:

$$\frac{dP^{\rm d}_{-1}}{dt} = -\alpha_{\rm d}(t)P^{\rm d}_{-1}(t) \tag{D.7.4}$$

$$\frac{dP^{\rm d}_{0}}{dt} = \alpha_{\rm d}(t)P^{\rm d}_{-1}(t) - n_{\rm s}\alpha_{\rm k}(t)P^{\rm d}_{0}(t) \tag{D.7.5}$$

$$\frac{dP^{\rm d}_{\rm i}}{dt} = (n_{\rm s} - i + 1)\alpha_{\rm k}(t)P^{\rm d}_{\rm i-1}(t) - (n_{\rm s} - i)\alpha_{\rm k}(t)P^{\rm d}_{\rm i}(t), \qquad i = 1,\ldots,n_{\rm s}-1 \tag{D.7.6}$$

$$\frac{dP^{\rm d}_{n_{\rm s}}}{dt} = \alpha_{\rm k}(t)P^{\rm d}_{n_{\rm s}-1}(t) \tag{D.7.7}$$

with the binding coefficient $\alpha_d(t)$ given by (C.7.1) and the phosphorylation coefficient $\alpha_k(t)$ given by (C.6.2). Notice that the phosphorylation terms in eqs.(D.7.5-7) account for the fact that, being in state i (i.e. i sites of Swi6 already phosphorylated), there are $n_s - i$ available sites for further phosphorylations. The model equations are such that $P^{d}_{n_s}(t) \to 1$ as $t \to \infty$ and $P^{d}_{i}(t) \to 0$ for i = -1, 0,…, n_s – 1. For this reason we say that the state $i = n_s$ is a *sink*.

In compact form, recalling that the probability vector $\mathbf{P^d}(t)$ is

$$\mathbf{P^d}(t) = [P^{\rm d}_{-1}(t)\ \ P^{\rm d}_{0}(t)\ \ \cdots\ \ P^{\rm d}_{n_{\rm s}}(t)]^T \in R^{n_{\rm d}} \tag{C.7.3}$$

with $n_d = n_s + 2$ it is:

$$\frac{d\mathbf{P^d}}{dt} = \left(\alpha_{\rm d}(t)D^{(\alpha_{\rm d})} + \alpha_{k}(t)D^{(\alpha_{\rm k})}\right)\mathbf{P^d}(t) \tag{D.7.8}$$

where $D^{(\alpha_d)}$, $D^{(\alpha_k)}$ are proper matrices in $Z^{n_d \times n_d}$ and $\mathbf{P^d}(0) = [1\ 0\ \ldots\ 0\,]^T \in Z^{n_d}$.

Numbering rows and columns with indexes $(i,j) \in \{-1,0,\ldots,n_s\} \times \{-1,0,\ldots,n_s\}$, by exploiting eqs.(D.7.4-7) the elements of matrix $D^{(\alpha_d)}$ are computed as:

$D^{(\alpha_d)}(-1,-1) = -1$, $\quad D^{(\alpha_d)}(0,-1) = 1$, $\quad D^{(\alpha_d)}(i,j) = 0$ for any other pair (i,j) (C.7.4)

On the other hand, the elements of matrix $D^{(\alpha_k)}$ are computed as follows:

- row -1: $D^{(\alpha_k)}(-1,j) = 0$, for all j
- row 0: $D^{(\alpha_k)}(0,0) = -n_s$, and $D^{(\alpha_k)}(0,j) = 0$, for all $j \neq 0$.

- row $i = 1,\ldots, n_s - 1$: $D^{(\alpha_k)}(i,i-1) = (n_s - i + 1)$, $D^{(\alpha_k)}(i,i) = -(n_s - i)$, and $D^{(\alpha_k)}(i,j) = 0$ for $j \notin \{i,i-1\}$
- row $i = n_s$: $D^{(\alpha_k)}(n_s,n_s-1) = 1$, and $D^{(\alpha_k)}(n_s,j) = 0$ for $j \notin \{n_s-1\}$

Notice that the vector $\mathbf{P^d}(t)$ is a vector of probabilities, and therefore its components must be nonnegative and such that at any time t the sum is 1, i.e. $\mathbf{1}^T_{ns}\ \mathbf{P^d}(t) = 1$ ($\mathbf{1}_{ns}$ being the vector of 1's in R^{ns}). This property is guaranteed at time $t = 0$ by the system initialization, where we set $P^d_{-1}(0) = 1$ and $P^d_i(0) = 0$ for $i = 0, 1,\ldots, n_s$. The structure of equation (D.7.8), where both $D^{(\alpha_d)}$ and $D^{(\alpha_k)}$ are Metzler matrices and such that $\mathbf{1}^T_{ns}D^{(\alpha_d)} = \mathbf{0}$ and $\mathbf{1}^T_{ns}D^{(\alpha_k)} = \mathbf{0}$, guarantees that $\mathbf{1}^T_{ns}\ \mathbf{P^d}(t) = 1$ for all $t > 0$.

Now let us consider the dynamics of the probabilities $P^t_{j1,j2,j3}(t)$ of the states of the SBF-affine genes, characterized by the triple (j_1, j_2, j_3) as explained in the previous section. A compact form analogous to (D.7.8) can be obtained by aggregating the probabilities $P^t_{j1,j2,j3}(t)$, by means of a lexicographic order, $(j_1, j_2, j_3) \in \{-1, 0, \ldots, n_s\} \times \{-1, 0, \ldots, n_{w1}\} \times \{-1, 0, \ldots, n_{w2}\}$, into the vector $\mathbf{P^t}(t) \in R^{nt}$ reported in (C.2.4.5). As in the case of the dynamics of $\mathbf{P^d}(t)$, also $\mathbf{P^t}(t)$ obeys a linear differential model that can be put in the form:

$$\frac{d\mathbf{P^t}}{dt} = \left(\alpha_{\rm t}(t)R^{(\alpha_{\rm t})} + \alpha_{\rm b}(t)R^{(\alpha_{\rm b})} + \alpha_{\rm k}(t)R^{(\alpha_{\rm k})} + \alpha_{\rm w}R^{(\alpha_{\rm w})}\right)\mathbf{P^t}(t) \qquad \text{(D.7.9)}$$

where $R^{(\alpha_t)}$, $R^{(\alpha_b)}$, $R^{(\alpha_k)}$, $R^{(\alpha_w)}$ are suitable matrices in $Z^{n_t \times n_t}$. In order to better understand the equation describing the dynamics of each component of $\mathbf{P^t}(t)$, the reader should look at the graph of Fig. 7.2.

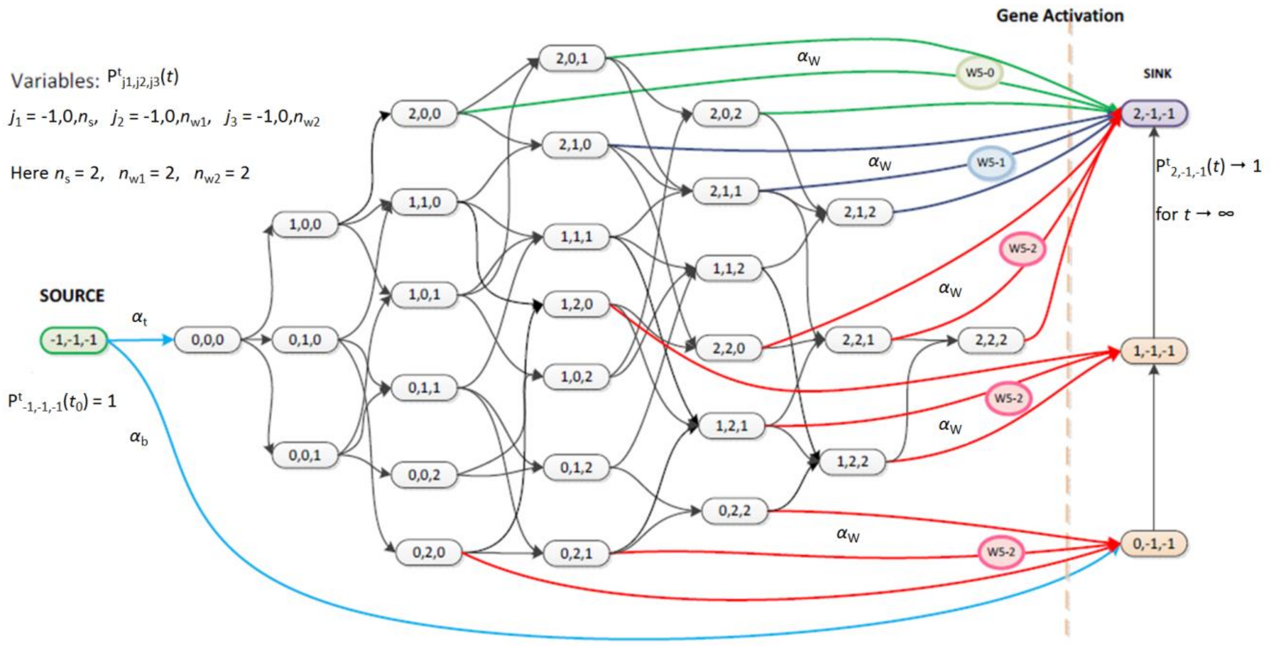

Figure 7.2. Graph representation of the transitions between states of SBF-affine genes for $n_s = n_{w1} = n_{w2} = 2$. The blue arrows represent the binding of the promoter to Swi6-P^0/Swi4/Whi5-P^{0,0}, with rate α_t, or to Swi6-P^0/Swi4, with rate α_b. The black arrows represent state transitions due to phospohorylation of free sites of Swi6-P^{j1}/Swi4/Whi5-P^{j2,j3}. The red, green and violet arrows represent the unbinding of Whi5 (and consequent gene activation), with different colors representing the release of Whi5 with different phosphorylation levels.

$$
\mathbf{P^t} = \left[\begin{array}{c}
P^{\mathrm{t}}_{-1,-1,-1} \\
P^{\mathrm{t}}_{0,-1,-1} \\
\vdots \\
P^{\mathrm{t}}_{n_{\mathrm{s}},-1,-1} \\
--- \\
P^{\mathrm{t}}_{-1,0,-1} \\
P^{\mathrm{t}}_{0,0,-1} \\
\vdots \\
P^{\mathrm{t}}_{n_{\mathrm{s}},0,-1} \\
--- \\
\vdots \\
--- \\
P^{\mathrm{t}}_{-1,n_{\mathrm{w1}}-1,-1} \\
P^{\mathrm{t}}_{0,n_{\mathrm{w1}}-1,-1} \\
\vdots \\
P^{\mathrm{t}}_{n_{\mathrm{s}},n_{\mathrm{w1}}-1,-1} \\
\hline
\bullet \\
\bullet \\
\bullet \\
\hline
P^{\mathrm{t}}_{-1,-1,n_{\mathrm{w2}}} \\
P^{\mathrm{t}}_{0,-1,n_{\mathrm{w2}}} \\
\vdots \\
P^{\mathrm{t}}_{n_{\mathrm{s}},-1,n_{\mathrm{w2}}} \\
--- \\
P^{\mathrm{t}}_{-1,0,n_{\mathrm{w2}}} \\
P^{\mathrm{t}}_{0,0,n_{\mathrm{w2}}} \\
\vdots \\
P^{\mathrm{t}}_{n_{\mathrm{s}},0,n_{\mathrm{w2}}} \\
--- \\
\vdots \\
--- \\
P^{\mathrm{t}}_{-1,n_{\mathrm{w1}}-1,n_{\mathrm{w2}}} \\
P^{\mathrm{t}}_{0,n_{\mathrm{w1}}-1,n_{\mathrm{w2}}} \\
\vdots \\
P^{\mathrm{t}}_{n_{\mathrm{s}},n_{\mathrm{w1}}-1,n_{\mathrm{w2}}}
\end{array}\right] \in R^{n_{\mathrm{t}}}
\tag{C.7.5}
$$

Notice that, in order to make the figure easy to understand, we have set $n_s = n_{w1} = n_{w2} = 2$ (instead of $n_s = n_{w1} = 4$ and $n_{w2} = 8$). The source node (–1, –1, –1) represents the genes whose promoters are free of the transcription factor SBF, and $P^t_{-1,-1,-1}(t)$ is the probability of an SBF-affine gene of being in such a state at time t. A gene can exit this state in two

ways: through the binding of Swi6/Swi4/Whi5 (transition (–1, –1, –1) → (0,0,0) with rate α_t) or through the binding of Swi6/Swi4 (transition (–1, –1, –1) → (0,–1, –1) with rate α_b). In this latter case, the gene activates. Thus the differential equation for $P^t_{-1,-1,-1}(t)$ is:

$$\frac{dP^{\mathrm{t}}_{-1,-1,-1}(t)}{dt} = -\big(\alpha_{\mathrm{t}}(t) + \alpha_{\mathrm{b}}(t)\big) P^{\mathrm{t}}_{-1,-1,-1}(t) \tag{D.7.10}$$

The equation for $P^t_{0,0,0}(t)$ is as follows:

$$\frac{dP^{\mathrm{t}}_{0,0,0}(t)}{dt} = -\alpha_{\mathrm{t}}(t) P^{\mathrm{t}}_{-1,-1,-1}(t) - (n_{\mathrm{s}} + n_{\mathrm{w1}} + n_{\mathrm{w2}})\alpha_{\mathrm{k}}(t) P^{\mathrm{t}}_{0,0,0}(t) \tag{D.7.11}$$

where the first term accounts for the increase of state (0,0,0) due to the binding of Swi6/Swi4/Whi5, and the second term describes the decrease of state (0,0,0) due to phosphorylations. More in details, we have three kind of transitions out of (0,0,0):

- (0,0,0) → (1,0,0) phosphorylation of Swi6 with rate $n_s\,\alpha_k$ (the free sites are n_s)
- (0,0,0) → (0,1,0) phosphorylation of functional sites of Whi5 with rate $n_{w1}\,\alpha_k$ (the free sites are n_{w1})
- (0,0,0) → (0,0,1) phosphorylation of decoy sites of Whi5 with rate $n_{w2}\,\alpha_k$ (the free sites are n_{w2})

The equations for the states $P^t_{j1,j2,j3}(t)$ when $0 < j_1 < n_s$, $0 < j_2 < n_{w1}$ and $0 < j_3 < n_{w2}$ are as follows. The probability of such a state can vary only because of phosphorylation processes:

$$\begin{aligned}\frac{dP^{\mathrm{t}}_{\mathrm{j_1,j_2,j_3}}(t)}{dt} =& -(n_{\mathrm{s}} - j_1 + n_{\mathrm{w1}} - j_2 + n_{\mathrm{w2}} - j_3)\alpha_{\mathrm{k}}(t) P^{\mathrm{t}}_{\mathrm{j_1,j_2,j_3}}(t)\\ &+ (n_{\mathrm{s}} - j_1 + 1)\alpha_{\mathrm{k}}(t) P^{\mathrm{t}}_{\mathrm{j_1-1,j_2,j_3}}(t)\\ &+ (n_{\mathrm{w1}} - j_2 + 1)\alpha_{\mathrm{k}}(t) P^{\mathrm{t}}_{\mathrm{j_1,j_2-1,j_3}}(t)\\ &+ (n_{\mathrm{w2}} - j_3 + 1)\alpha_{\mathrm{k}}(t) P^{\mathrm{t}}_{\mathrm{j_1,j_2,j_3-1}}(t)\end{aligned} \tag{D.7.12}$$

The first (negative) term describes the decrease of the number of genes in states (j_1, j_2, j_3) due to phosphorylation of one of the admissible sites: Swi6 with rate $(n_s - j_1)\alpha_k$, functional sites of Whi5 with rate $(n_{w1} - j_2)\alpha_k$, and decoy sites of Whi5 with rate $(n_{w2} - j_3)\alpha_k$. The following three positive terms account for the increase of the number of genes in state (j_1, j_2, j_3) coming from genes in state ($j_1 - 1, j_2, j_3$) (through phosphorylation of Swi6-P^{j1-1} with rate $(n_s - j_1 + 1)\alpha_k$), from genes in state ($j_1, j_2 - 1, j_3$) (through phosphorylation of Whi5-$P^{j2-1,j3}$ with rate $(n_{w1} - j_2 + 1)\alpha_k$), or from genes in state ($j_1, j_2, j_3 - 1$) (through phosphorylation of Whi5-$P^{j2,j3-1}$ with rate $(n_{w2} - j_3 + 1)\alpha_k$).

The dynamics of the probability relative to the state (0,–1,–1) (genes activated and bound

to Swi6-P^0/Swi4) is the following:

$$\frac{dP^{\mathrm{t}}_{0,-1,-1}(t)}{dt} = \alpha_{\mathrm{b}}(t)P^{\mathrm{t}}_{-1,-1,-1}(t) - n_{\mathrm{s}}\alpha_{\mathrm{k}}(t)P^{\mathrm{t}}_{0,-1,-1} + \alpha_{\mathrm{k}}(t)\sum_{j_3=0}^{n_{\mathrm{w}2}} P^{\mathrm{t}}_{0,n_{\mathrm{w}1}-1,\mathrm{j}_3}(t)$$

(D.7.13)

The first term explains the growth due to the direct binding of the promoter to Swi6/Swi4 (free of Whi5). The second term accounts for the phosphorylation of a site in Swi6 (transition (0,–1,–1) → (1,–1,–1)). The last summation collects the contributions due to all transitions from states (0,n_{w1},j_3) to (0,–1,–1) due to the release of Whi5-P^{n_{w1},j_3}, which occurs at rate α_{w}.

The states of the type (j_1,–1,–1) with j_1 = 1, …, n_{s} – 1, obey to an equation similar to the previous one, but where the contribution of transitions from (–1,–1,–1) is replaced by transitions from (j_1–1,–1,–1) (the first term in the equation):

$$\frac{dP^{\mathrm{t}}_{\mathrm{j}_1,-1,-1}(t)}{dt} = (n_{\mathrm{s}} - j_1 + 1)\alpha_{\mathrm{k}}(t)P^{\mathrm{t}}_{j_1,-1,-1}(t) - (n_{\mathrm{s}} - j_1)\alpha_{\mathrm{k}}(t)P^{\mathrm{t}}_{\mathrm{j}_1,-1,-1} + \alpha_{\mathrm{k}}(t)\sum_{j_3=0}^{n_{\mathrm{w}2}} P^{\mathrm{t}}_{\mathrm{j}_1,\mathrm{n}_{\mathrm{w}1}-1,\mathrm{j}_3}(t)$$

(D.7.14)

The state (n_{s}, –1,–1) is the *sink* state of the net, in that asymptotically all genes tend to be in such a state (i.e., $P^{\mathrm{t}}_{(n\mathrm{s},\,-1,-1)}(t) \to 1$ as $t \to \infty$). The dynamics of $P^{\mathrm{t}}_{(n\mathrm{s},\,-1,-1)}(t)$ is:

$$\frac{dP^{\mathrm{t}}_{n_{\mathrm{s}},-1,-1}(t)}{dt} = \alpha_{\mathrm{k}}(t)P^{\mathrm{t}}_{n_{\mathrm{s}},-1,-1}(t) + \alpha_{\mathrm{w}}\sum_{j_2=0}^{n_{\mathrm{w}1}-1}\sum_{j_3=0}^{n_{\mathrm{w}2}} P^{\mathrm{t}}_{n_{\mathrm{s}}-1,j_2,j_3}(t)$$

(D.7.15)

Only non-negative terms are present (monotonic growth of $P^{\mathrm{t}}_{(n_{\mathrm{s}},\,-1,-1)}(t)$). The first term is due to the phosphorylation of the last site of Swi6 in Swi6-P^{ns-1}/Swi4; the double summation accounts for the release of Whi5 according to different functional and decoy phosphorylation configurations.

Equations (D.7.10-15) can be put in the compact form (D.7.9) with suitable definitions of the matrices $R^{(\alpha_{\mathrm{t}})}$, $R^{(\alpha_{\mathrm{b}})}$, $R^{(\alpha_{\mathrm{k}})}$, $R^{(\alpha_{\mathrm{w}})}$. It can be shown that these matrices are all Metzler, and this guarantees that if at 0 the components of $\mathbf{P}^{\mathbf{t}}$ are non-negative, they remain non-negative for all $t > 0$. Moreover, they are such that $\mathbf{1}^{\mathrm{T}}R^{(\alpha_{\mathrm{t}})} = \mathbf{0}$, $\mathbf{1}^{\mathrm{T}}R^{(\alpha_{\mathrm{b}})} = \mathbf{0}$, $\mathbf{1}^{\mathrm{T}}R^{(\alpha_{\mathrm{k}})} = \mathbf{0}$, $\mathbf{1}^{\mathrm{T}}R^{(\alpha_{\mathrm{w}})} = \mathbf{0}$, and this ensures that if $\mathbf{1}^{\mathrm{T}}\mathbf{P}^{\mathbf{t}}(0) = 1$, then $\mathbf{1}^{\mathrm{T}}\mathbf{P}^{\mathbf{t}}(t) = 1$ for all $t > 0$. Of course, at $t = 0$ all SBF-affine genes are in the state (–1,–1,–1) (source state), and therefore $P^{\mathrm{t}}_{-1,-1,-1}(0) = 1$, and $P^{\mathrm{t}}_{\mathrm{j}1,\,\mathrm{j}2,\,\mathrm{j}3}(0) = 0$ for (j_1, j_2, j_3) ≠ (–1,–1,–1) (thus $\mathbf{1}^{\mathrm{T}}\mathbf{P}^{\mathbf{t}}(0) = 1$).

According to $\mathbf{P}^{\mathbf{d}}$(t) and $\mathbf{P}^{\mathbf{t}}$(t) definitions, the following quantities can be computed.

- a^{B}, probability that a class B gene is activated (providing an estimate of the percentage of class B activated genes):

$$a^{\mathrm{B}}(t) = P^{\mathrm{d}}_{n_{\mathrm{s}}}(t) = F^{\mathrm{d}} \cdot \mathbf{P^d}(t), \qquad F^{\mathrm{d}} = [0 \ \ \cdots \ \ 0 \ \ 1] \in R^{1\times n_{\mathrm{d}}} \tag{C.7.6}$$

Its computation comes from the fact that a class B gene is an MBF-affine gene
- a^{A}, probability that a class A gene is activated (providing an estimate of the percentage of class A activated genes). Since a class A gene is an SBF-affine gene, a^{A} is obtained by summing up all the SBF-affine gene probabilities to be bound to SBF free of Whi5:

$$a^{\mathrm{A}}(t) = \sum_{j_1=0}^{n_{\mathrm{s}}} P^{\mathrm{t}}_{j_1,-1,-1}(t) = F^{\mathrm{t}} \cdot \mathbf{P^t}(t), \qquad F^{\mathrm{t}} \in R^{1\times n_{\mathrm{t}}} \tag{C.7.7}$$

F^{t} is a suitably defined vector with entries in {0,1}.
- a^{C}, probability that a class C gene is activated (providing an estimate of the percentage of class C activated genes). Class C genes are given by the intersection of SBF-affine and MBF-affine genes. Thus, the probability that a class C gene is activated is given by the probability that an SBF-affine gene is activated + the probability that an MBF-affine gene is activated - the joint probability (given by the product of probabilities, due to the independence of the events):

$$a^{\mathrm{C}}(t) = a^{\mathrm{A}}(t) + a^{\mathrm{B}}(t) - a^{\mathrm{A}}(t)a^{\mathrm{B}}(t) \tag{C.7.8}$$

- u^{d}, mean value of free phosphorylation sites for an MBF-affine gene:

$$u^{\mathrm{d}}(t) = U^{\mathrm{d}} \cdot \mathbf{P^d}(t), \qquad U^{\mathrm{d}} = [0 \ \ n_{\mathrm{s}} \ \ n_{\mathrm{s}}-1 \ \ \cdots \ \ 1 \ \ 0] \in R^{1\times n_{\mathrm{d}}} \tag{C.7.9}$$

- f^{B}, free phosphorylation sites for class B genes:

$$f^{\mathrm{B}}(t) = N_{\mathrm{B}} \cdot u^{\mathrm{d}}(t) \tag{C.7.10}$$

- u^{t}, mean value of free phosphorylation sites for an SBF-affine genes:

$$u^{\mathrm{t}}(t) = \sum_{j_1=0}^{n_{\mathrm{s}}-1} \sum_{j_2=0}^{n_{\mathrm{w1}}-1} \sum_{j_3=0}^{n_{\mathrm{w2}}} \left((n_{\mathrm{s}} - j_1) + (n_{\mathrm{w1}} - j_2) + (n_{\mathrm{w2}} - j_3)\right) P^{\mathrm{t}}_{j_1,j_2,j_3}(t) = U^{\mathrm{r}} \cdot \mathbf{P^t}(t) \tag{C.7.11}$$

The sum is intended to contain only admissible states for triples (j_1, j_2, j_3). U^{t} is a suitable matrix in Z^{1xnt}.
- f^{A}, free phosphorylation sites for class A genes:

$$f^{\mathrm{A}}(t) = N_{\mathrm{A}} \cdot u^{\mathrm{t}}(t) \tag{C.7.12}$$

- f^{C} , free phosphorylation sites for class C genes:

$$f^{\mathrm{C}}(t) = N_{\mathrm{C}} \cdot \left(u^{\mathrm{t}}(t) + u^{\mathrm{d}}(t)\right) \tag{C.7.13}$$

The sum $f^A + f^B + f^C$ provides the sum of all the free phosphorylation sites, and is exploited for the computation of N^{free} in (C.6.2).

As for Whi5 dynamics, it is released from the genes where Swi6Swi4Whi5 is bound at the end of the complete phosphorylation process of either Swi6 or functional sites of Whi5 in class A and C genes. To model the Whi5 release, define the vector

$$\mathbf{W_p} = \begin{bmatrix} Whi5 \\ Whi5p \\ \vdots \\ Whi5p^{n_{\mathrm{w1}}} \end{bmatrix} \in R^{n_{\mathrm{w1}}+1} \tag{C.7.14}$$

where Whi5-P^{j_2}, j_2 = 0, …, n_{w1}, refers to a free molecule of Whi5 that is j_2-times phosphorylated on functional sites, and any time phosphorylated on decoy sites. As far as the case of Whi5-P^{j_2} for j_2 = 0, …, $n_{\mathrm{w1-1}}$, it is:

$$\frac{dWhi5p^{j_2}}{dt} = \cdots + \alpha_{\mathrm{w}}(N_{\mathrm{A}} + N_{\mathrm{C}}) \sum_{j_3=0}^{n_{\mathrm{w2}}} P^{\mathrm{t}}_{n_{\mathrm{s}},j_2,j_3}(t) \tag{D.7.16}$$

accounting for the release of Whi5-P^{j_2} after the last phosphorylation of Swi6 in SBF-affine genes; it sums for all the j_3 possible phosphorylation states of the decoy sites of Whi5. As far as the case $j_2 = n_{\mathrm{w1}}$, it is:

$$\frac{dWhi5p^{n_{\mathrm{w1}}}}{dt} = \cdots + \alpha_{\mathrm{w}}(N_{\mathrm{A}} + N_{\mathrm{C}}) \sum_{j_1=0}^{n_{\mathrm{s}}-1} \sum_{j_3=0}^{n_{\mathrm{w2}}} P^{\mathrm{t}}_{j_1,n_{\mathrm{w1}},j_3}(t) \tag{D.7.17}$$

accounting for the release of Whi5-P$^{n_{\mathrm{w1}}}$ after the last phosphorylation of the functional sites of Whi5; it sums for all the j_1 possible phosphorylation states of Swi6 and for all the j_3 possibilities of the decoy sites of Whi5. In a more compact form, there exists a suitably dimensioned matrix H_{t} such that:

$$\frac{d\mathbf{W_p}}{dt} = \cdots + \alpha_{\mathrm{w}}(N_{\mathrm{A}} + N_{\mathrm{C}}) H_{\mathrm{t}} \mathbf{P^t}(t), \qquad H_{\mathrm{t}} \in \{0,1\}^{(n_{\mathrm{w1}}+1)\times n_{\mathrm{t}}} \tag{D.7.18}$$

Nuclear Whi5 is released out of the nucleus according to diffusion coefficients increasing with the number of functional phosphorylated states. If we aggregate these coefficients in $\mathbf{k_{21}} = [k^0{}_{21}\ k^1{}_{21}\ \ldots\ k^{nw1}{}_{21}]^{\mathrm{T}}$, then we have that the contribute to $\mathbf{W_p}$ dynamics due to the diffusion out of the nucleus is:

$$\frac{d\mathbf{W_p}}{dt} = \cdots - \mathrm{diag}\{\mathbf{k_{21}}\} \mathbf{W_p}(t) \tag{D.7.19}$$

with $diag\{\mathbf{k_{21}}\}$ a diagonal matrix with the diagonal elements given by the entries of vector $\mathbf{k_{21}}$. Thus, in summary, taking into account also the binding dynamics of (R.7.3):

$$\frac{d\mathbf{W_p}}{dt} = \left(-\frac{k_{14}}{V_{\text{nuc}}(t)} Swi6Sw4(t) \cdot Whi5_{\text{nuc}}(t) + k_{13} Swi6Swi4Whi5(t) \right) \begin{bmatrix} 1 \\ 0 \\ \vdots \\ 0 \end{bmatrix} + \alpha_{\text{w}}(N_{\text{A}} + N_{\text{C}}) H_{\text{t}} \mathbf{P^t}(t) - \text{diag}\{\mathbf{k_{21}}\} \mathbf{W_p}(t) \tag{D.7.20}$$

Finally, the following equations refer to the constraints given by the fact that the total number of Swi6, Swi4, Mbp1 and Whi5 is constant within the G_1 period of interest:

$$Swi6(t) + Swi6Swi4(t) + Swi6Swi4Whi5(t) + (N_{\text{A}} + N_{\text{C}}) \cdot \left(1 - P^{\text{t}}_{-1,-1,-1}(t)\right) + (N_{\text{B}} + N_{\text{C}}) \cdot \left(1 - P^{\text{d}}_{-1}(t)\right) = Swi6_{\text{tot}} \tag{C.7.15}$$

$$Swi4(t) + Swi6Swi4(t) + Swi6Swi4Whi5(t) + (N_{\text{A}} + N_{\text{C}}) \cdot \left(1 - P^{\text{t}}_{-1,-1,-1}(t)\right) = Swi4_{\text{tot}} \tag{C.7.16}$$

$$Mbp1(t) + Swi6Mbp1(t) + (N_{\text{B}} + N_{\text{C}}) \cdot \left(1 - P^{\text{d}}_{-1}(t)\right) = Mbp1_{\text{tot}} \tag{C.7.17}$$

$$\mathbf{1}^T_{n_{\text{w1}}+1} \mathbf{W_p}(t) + Swi6Swi4Whi5(t) + (N_{\text{A}} + N_{\text{C}})\left(1 - P^{\text{t}}_{-1,-1,-1}(t) - a^{\text{A}}(t)\right) + Whi5_{\text{cyt}}(t) = Whi5_{\text{tot}} \tag{C.7.18}$$

Thus, according to eqs.(C.7.15-18) it is possible to compute Swi4, Swi6, Mbp1 and $Whi5_{cyt}$ from the other state variables.

Supplementary Note 8

Extension of the G_1/S model to Whi5 mutants

Biology. Data on *whi5* null mutant and mutants with alanine substitutions in either the functional and/or the decoy Cdk phosphorylation sites are available and have been described in the main text and this document (Supplementary Note 6). Data on mutants with phosphor-mimetic mutations in the functional sites were not previously available. Our newly generated data, that have been used for validation of our model are presented in the main text (Fig. 6).

Reconstruction. The G_1/S model accounts for numerical simulations involving the *whi5 deleto*, with no structure modifications, except for by setting the total amount of Whi5 equal to zero in constraint (C.7.18).

On the other hand, some modifications are required to model the mutant $Whi5^{4E}$ supposed to have its 4 functional sites 'naturally' phosphorylated. The working hypothesis is that, differently from WT cells where phosphorylated Whi5 does not bind to SBF, such a mutant Whi5 keeps unchanged the possibility to bind to (and therefore inhibit) SBF. Such a binding occurs with a lower affinity, that means reaction (R.7.3) occurs with a lower value of k_{14}. Moreover, because of the '*naturally*' phosphorylation state of Whi5, the binding of SBFWhi5 to the gene modifies its state from (-1,-1,-1) straightforwardly to $(0,n_{w1},0)$, which prevents to pass through most of the possible states (see the scheme of Fig. 8.1). Finally, soon after the binding of SBFWhi5, Whi5 is ready to be released without waiting for further phosphorylations. However, this happens at a lower rate.

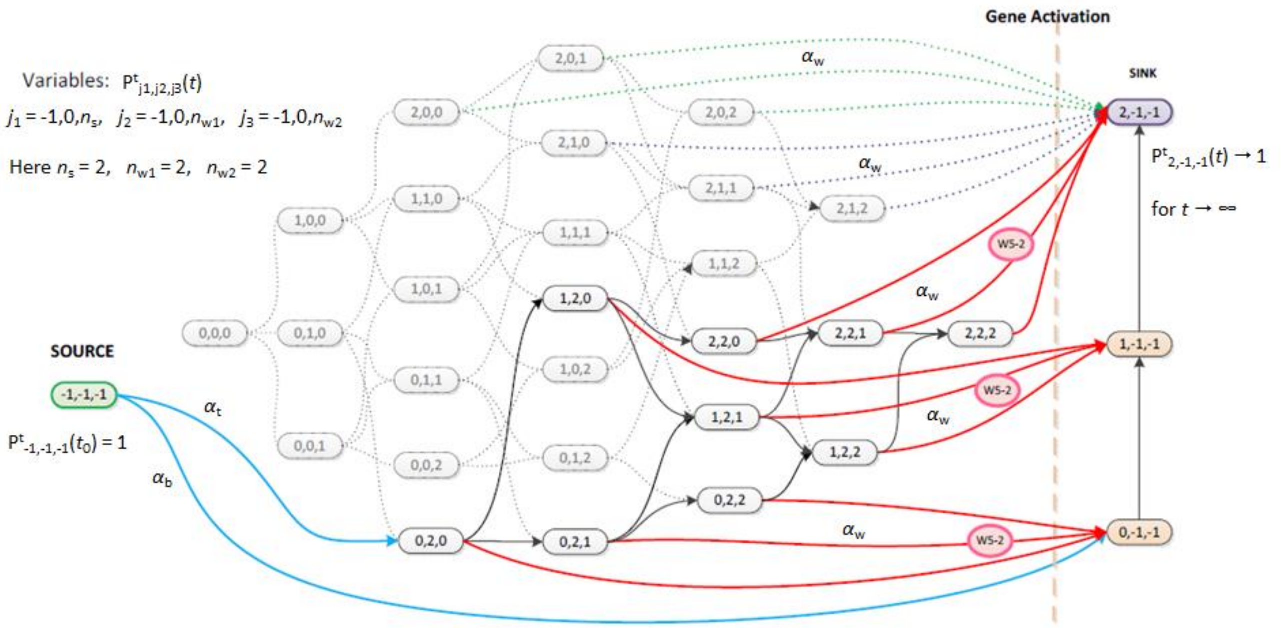

Figure 8.1. Graph representation of the transitions between states of a mutant cell with the Whi5 functional states already phosphorylated, for SBF-affine genes with $n_s = n_{w1} = n_{w2} = 2$. Notice that the figure reports all the states of the WT case reported in Fig. 1.2.4.2, keeping transparent the ones not compatible with the mutant case. Like in Fig. 1.2.4.2, the black arrows represent state transitions due to phospohorylation of free sites of Swi6-P^{j1}/Swi4/Whi5-$P^{j2,j3}$. The red, green and violet arrows represent the unbinding of Whi5 (and consequent gene activation), with different colors representing the release of Whi5 with different phosphorylation levels.

Notice that, according to the mutant properties, there exists only one state for Whi5 to be modeled, that is Whi5-P^{nw1}.

Computational representation. Besides the building of the matrices $R^{(\alpha_t)}$, $R^{(\alpha_b)}$, $R^{(\alpha_k)}$, $R^{(\alpha_w)}$ related to the vector $\mathbf{P^t}$, eq.(D.7.9) does not formally change. As for Whi5, eqs.(D.7.1-2) are still valid, with $Whi5_{nuc}$ meaning the mutant (and unique) form of $Whi5^{4E}$. On the other hand, eq.(D.7.20) simplifies in:

$$\frac{dWhi5_{\rm nuc}(t)}{dt} = -\frac{k_{14}}{V_{\rm nuc}(t)} Swi6Swi4(t)Whi5_{\rm nuc}(t) + k_{13}Swi6Swi4Whi5(t) + \alpha_{\rm w}(N_{\rm A}+N_{\rm C})H_{\rm t}\mathbf{P^t}(t) - k_{21}^4 Whi5_{\rm nuc}(t) \quad \text{(D.8.1)}$$

Supplementary Note 9

Expression of relevant controller genes of the G_1/S regulon

Biology. In any given growth condition or following release from any cell cycle arrest, genes within the G_1/S regulon have a well-defined distribution of transcriptional activation times. Namely, activation of the *CLN2* and – more so – *CLN1* genes consistently anticipates activation of the bulk of the G_1/S regulon, while activation of the *CLB5,6* genes – leading to synthesis of Clb5,6 cyclins required for S phase entrance - and of the *NRM1* gene encoding the MBF inhibitor protein follow at later and reproducible times [60].

Reconstruction. The last phase of the G_1/S transition is regulated by 5 of the 235 molecules encoded by the G_1/S regulon, namely Cln2 and Nrm1 (encoded by class A genes), Clb5 (encoded by class B genes), Cln1 and Clb6 (encoded by class C genes), [60]. Their temporal pattern of expression is robust and likely it is a function of the number and sequence of the SBF and/or MBF binding sites present upstream of each gene. We model the temporal pattern of expression of these 5 gene products as follows. Their production starts according to the transcriptional activation of the corresponding gene. To this aim, each of the 5 players is associated to a random distribution indicating the order β of activation with respect to the other genes of the class they belong. For any given cell, a sample is drawn from such distributions for each of the 5 players, and the corresponding gene is properly activated as soon as the percentage of activated genes of the class (estimated by the probability of activation of a gene of the class) includes the first β genes. Each distribution is chosen log-normal with average value η and standard deviation σ: $\beta = \log N(\eta,\sigma)$. This assumption clearly prevents the possibility to have negative values for

beta, but does not ensure that the sample is smaller than the maximum value of the corresponding class: as it will be clearer in the following, parameters are set in order to make negligible such a probability, which would mean that that gene is never activated. So, for example, the fact that *CLN1* gene is among the first few genes to be activated in each cell is modeled by setting a low value for the corresponding average value η, but whether it will be the first, the second or any order gene to be activated within its class, it depends from a random sample drawn from the corresponding distribution for each individual cell.

Besides the proper expression by means of their proper transcription factor activation, *CLN1*, *CLN2*, *NRM1*, *CLB5*, *CLB6* can be *weakly* expressed (i.e. with a production rate lower than that obtained after the proper activation) starting from a random time. For any given cell, a sample is drawn, for each of the five players, from a log-normal distribution assigning that player a time Δ, according to which the weak expression occurs in case the proper activation is delayed. In case the proper activation takes place before the random time (as it usually happens for wild type cells), the weak expression does not occur. On the other hand, if the weak expression occurs, then it may switch to the proper expression as soon as the proper activation starts.

Finally, Nrm1 has also the role to inhibit MBF, as soon as it accumulates over a given threshold. As a matter of fact, when this happens regular Clb5 production is switched off (since it belongs to class B genes, expressed by only MBF). However, Clb5 can still be weakly produced, due to the weak activation. Such a threshold is set, in average cells, so that Nrm1 exceeds it close to the end of the G_1 phase, or even later [60].

<u>*Computational representation*</u>. *CLN2* and *NRM1* are supposed to be expressed within the first half and the second half of activated class A genes, respectively. Thus, by defining β_{Cln2} and β_{Nrm1} the order of activation of *CLN2* and *NRM1*, respectively, they are set as follows:

$$\beta_{Cln2} = \log N(30,10), \quad \text{with } P(\beta_{Cln2} < 5) \approx 0\% \quad P(\beta_{Cln2} < 68) = 99.6\% \qquad (C.9.1)$$

$$\beta_{Nrm1} = \log N(110,10), \quad \text{with } P(\beta_{Nrm1} < 5) \approx 0\% \quad P(\beta_{Nrm1} > 68) \approx 100\% \qquad (C.9.2)$$

In this way, in wild type cases, there is a negligible probability to let them be activated by the direct binding of Swi6Swi4, if we set the model parameters in order to keep the percentage of class A genes activated by the direct binding of Swi6Swi4 less than 5%. Moreover, the probability for *CLN2* to be expressed within the first half of activated class A genes is very close to 100% as well as very close to 100% is the probability for *NRM1* to

be expressed within the second half of activated class A genes.

CLB5 is supposed to be expressed within the second half of activated class B genes, with the following distribution for its order of activation β_{Clb5}:

$$\beta_{Clb5} = \text{logN}(45,5), \quad \text{with} \quad P(\beta_{Clb5} > 32) = 99.9\% \qquad \text{(C.9.3)}$$

This way, the probability to be expressed within the second half of activated class B genes is very close to 100%.

CLN1 and *CLB6* are supposed to be expressed within the first half and the second half of activated class C genes, respectively, with the following distributions for the orders of activation β_{Cln1}, β_{Clb6}:

$$\beta_{Cln1} = \text{logN}(5,10), \quad \text{with} \quad P(\beta_{Cln1} < 2) = 46.5\% \quad P(\beta_{Cln1} < 18) = 95.0\% \qquad \text{(C.9.4)}$$

$$\beta_{Clb6} = \text{logN}(20,2), \quad \text{with} \quad P(\beta_{Clb6} < 5) \approx 0\% \quad P(\beta_{Clb6} > 18) = 84.3\% \qquad \text{(C.9.5)}$$

Following the chosen probability distribution, *CLN1* transcription has about 50% of probability to be expressed within the very first set of activated class C genes, and a high probability to be activated within the first half of class C genes. On the other hand, the probability for *CLB6* gene to be activated in the second half of class C genes is high, keeping negligible the probability to be activated within the very first genes.

The SBF/MBF-independent activation of *CLN1* and *CLN2*, due to the random times Δ_{Cln1} and Δ_{Cln2}, occurs according to the same following distributions:

$$\Delta_{Cln1} = \Delta_{Cln2} = \Delta_{Cln1,2} = \text{logN}(30,10), \quad \text{with} \quad P(\Delta_{Cln1,2} < 15) = 2.4\% \quad P(\Delta_{Cln1} < 50) = 95.9\% \qquad \text{(C.9.6)}$$

In this way, *CLN1* and *CLN2* would be weakly expressed within the first 15min (a time smaller than the proper activation in wild type cells) with a very low probability. On the other hand, the probability to have at least the weak activation within the first 50min is very high.

NRM1, *CLB5*, *CLB6* weak activation, due to the random times Δ_{Nrm1}, Δ_{Clb5}, Δ_{Clb6}, occurs according to the following distributions:

$$\Delta_{Clb5,6} = \text{logN}(60,10), \quad \text{with} \quad P(\Delta_{Clb5,6} < 40) = 0.9\% \quad P(\Delta_{Clb5,6} < 80) = 96.6\% \qquad \text{(C.9.7)}$$

$$\Delta_{Nrm1} = \text{logN}(60,10), \quad \text{with} \quad P(\Delta_{Nrm1} < 40) = 0.9\% \quad P(\Delta_{Nrm1} < 80) = 96.6\% \qquad \text{(C.9.8)}$$

This way, *NRM1*, *CLB5* and *CLB6* would be rarely expressed, even weakly, before 40min, that is a time greater than the proper activation. On the other hand, the probability to have at least the weak activation within the first 80min is very high.

Below, the equations for Cln1, Cln2 and Nrm1 follow:

$$\frac{dCln2}{dt} = k_{23}V_{\text{nuc}}(t) - k_{22}Cln2(t) \tag{D.9.1}$$

$$\frac{dNrm1}{dt} = k_{25}V_{\text{nuc}}(t) - k_{24}Nrm1(t) \tag{D.9.2}$$

$$\frac{dCln1}{dt} = k_{27}V_{\text{nuc}}(t) - k_{26}Cln1(t) \tag{D.9.3}$$

where k_{22}, k_{24}, k_{26} are the clearance rates and k_{23}, k_{25}, k_{27} are the production rates (these latter in terms of molarity min^{-1}: hence the necessity of the nuclear volume to build the production rate in terms of number of molecules min^{-1}) obeying the above mentioned rules for proper and weak activation, formally stated by the following equations:

$$k_{23} = \begin{cases} k_{23\text{H}} & \text{if} \quad a^{\text{A}}(t) \cdot N_{\text{A}} \geq \beta_{\text{Cln2}} \\ k_{23\text{L}} & \text{if} \quad (t > \Delta_{\text{Cln2}}) \quad AND \quad (a^{\text{A}}(t) \cdot N_{\text{A}} < \beta_{\text{Cln2}}) \\ 0 & \text{otherwise} \end{cases} \tag{C.9.9}$$

$$k_{25} = \begin{cases} k_{25\text{H}} & \text{if} \quad a^{\text{A}}(t) \cdot N_{\text{A}} \geq \beta_{\text{Nrm1}} \\ k_{25\text{L}} & \text{if} \quad (t > \Delta_{\text{Nrm1}}) \quad AND \quad (a^{\text{A}}(t) \cdot N_{\text{A}} < \beta_{\text{Nrm1}}) \\ 0 & \text{otherwise} \end{cases} \tag{C.9.10}$$

$$k_{27} = \begin{cases} k_{27\text{H}} & \text{if} \quad (\chi_{\{\text{Nrm1}<\Theta_{\text{Nrm1}}\}}(t)a^{\text{C}}(t) + \chi_{\{\text{Nrm1}\geq\Theta_{\text{Nrm1}}\}}(t)a^{\text{A}}(t))N_{\text{C}} \geq \beta_{\text{Cln1}} \\ k_{27\text{L}} & \text{if} \quad (t > \Delta_{\text{Cln1}}) \quad AND \quad \left((\chi_{\{\text{Nrm1}<\Theta_{\text{Nrm1}}\}}(t)a^{\text{C}}(t) + \chi_{\{\text{Nrm1}\geq\Theta_{\text{Nrm1}}\}}(t)a^{\text{A}}(t))N_{\text{C}} < \beta_{\text{Cln1}}\right) \\ 0 & \text{otherwise} \end{cases} \tag{C.9.11}$$

Function $\chi_A(t)$ exploited in (C.9.11) denotes a function that is equal to 1 for all the values of t according to which condition A is satisfied, and zero otherwise. It helps to properly take into account the inhibitory role of Nrm1, exerted when it exceeds the threshold Θ_{Nrm1}. Indeed, if $Nrm1(t) > \Theta_{\text{Nrm1}}$, then class C genes behave like class A genes because MBF is inhibited by Nrm1.

Supplementary Note 10

Inhibition of Clb5,6-Cdk1 kinase activity by Sic1

Biology. Clb5,6-Cdk1 (S-Cdk) activity is responsible for initiation of DNA replication [24,69]. As reviewed in [70], as soon as Clb5,6 molecules build-up, their catalytic activity is inhibited by Sic1, a stoichiometric inhibitor. In order for S-Cdk activity to be expressed, the Sic1

inhibitor needs to be degraded. Sic1 degradation is primed by Cdk1 phosphorylation. Phosphorylation of at least 6 out of 9 total phospho-degrons is required [71]. Recent experimental evidence indicates that, contrary to earlier belief, both Cln-Cdk1 and Clb-Cdk1 activity contribute to Sic1 phosphorylation thereby generating an AND control [23] which acts as a molecular threshold: in fact, Sic1 is degraded when the amount of Clb5,6-Cdk1 in the cell overcomes the amount that can be bound by Sic1. Following phosphorylation, Sic1 dissociates from Clb5,6-Cdk1, is exported from the nucleus and eventually is degraded. As a result, Clb5,6-Cdk1 activity sharply builds up allowing coherent firing of DNA replication origins [24]. The Sic1 control activity on cyclins Clb5,6 is exerted in Timer T_2, that starts when cytoplasmic Whi5 exceeds 50% of the total amount of Whi5 and ends when 50% of the total amount of Sic1 has been exported from the nucleus.

Reconstruction. A newborn cell does not contain any Clb5 and Clb6 at the beginning of the cycle, and it requires the activation of SBF/MBF before starting transcription from the respective encoding genes. However, free Clb5 and Clb6 are made inactive by the inhibitor Sic1 in the complexes Clb5Sic1 and Clb6Sic1, due to the large amount of Sic1 present in a newborn cell [70]:

Clb5 + Sic1 ⇆ Clb5Sic1 Clb6 + Sic1 ⇆ Clb6Sic1 (R.10.1)

Clb5 and Clb6 are freed from Sic1 by means of the phosphorylation of Sic1 alone and in the complexes Clb5Sic1, Clb6Sic1. Such phosphorylations are catalyzed by two sets of kinases. From one hand there are the cyclins Cln1,2,3 that work in sum according to the following proportion:

$$g\big(Cln_{1,2,3}(t)\big) = Cln1(t) + Cln2(t) + 0.1 \cdot Cln3(t) \tag{C.10.1}$$

In other words, Cln1,2-Cdk1 is taken to be the main Cln activity involved in Sic1 degradation, Cln3-Cdk1 taking over in case of deletion of the *CLN1,2* genes [71]. Moreover, their contribute passes through a smooth Hill function (see (C.10.2) below). From the other hand there are cyclins Clb5,6 who work in sum as well: Clb5 + Clb6. These two sets of kinases work according to an AND logic: both must be present in order for phosphorylation of Sic1 to occur [23,72]. Thus, the following phosphorylation coefficient is considered:

$$\gamma_i(t) = \frac{k_i}{V_{\text{nuc}}(t)} \cdot \frac{g\big(Cln_{1,2,3}(t)\big)^{n_i}}{\Theta_i^{n_i} + g\big(Cln_{1,2,3}(t)\big)^{n_i}} \cdot \big(Clb5(t) + Clb6(t)\big) \tag{C.10.2}$$

with the suffix i = 40, 41, 42 related to the phosphorylation of Sic1 alone and in the complexes Clb6Sic1 and Clb5Sic1, respectively.

Once phosphorylated, complexes Clb5Sic1-P and Clb6Sic1-P are degraded, releasing

active Clb5 and Clb6:

Clb5Sic1-P ⇆ Clb5 + Sic1-P Clb6Sic1-P ⇆ Clb6 + Sic1-P (R.10.2)

Finally, phosphorylated Sic1 is cleared out from the nucleus.

In keeping with formalism used for defining the end of the T_1 period, we assume that triggering of DNA replication takes place when the fraction of nuclear Sic1 drops below 50%. A more detailed model of the onset of DNA replication and of its dependence from the availability of Clb5,6-Cdk1 has been presented [24].

Below is the constraint referring to the fact that the total amount of Sic1 is kept constant:

$$Sic1_{\text{tot}} = Sic1_{\text{nuc}}(t) + Clb5Sic1(t) + Clb5Sic1p(t) + Clb6Sic1(t) + Clb6Sic1p(t) + Sic1p(t) + Sic1_{\text{cyt}}(t)$$ (C.10.3)

Computational representation. Below follow the dynamic equations for Clb6, Clb5, Clb6Sic1, Clb5Sic1, Clb6Sic1-P, Clb5Sic1-P, $Sic1_{nuc}$ and Sic1-P.

$$\frac{dClb6}{dt} = k_{29}V_{\text{nuc}}(t) - k_{28}Clb6(t) - \frac{k_{31}}{V_{\text{nuc}}(t)}Sic1_{\text{nuc}}(t)\cdot Clb6(t) + k_{30}Clb6Sic1(t) - \frac{k_{33}}{V_{\text{nuc}}(t)}Clb6(t)\cdot Sic1p(t) + k_{32}Clb6Sic1p(t)$$ (D.10.1)

$$\frac{dClb5}{dt} = k_{35}V_{\text{nuc}}(t) - k_{34}Clb5(t) - \frac{k_{37}}{V_{\text{nuc}}(t)}Sic1_{\text{nuc}}(t)\cdot Clb5(t) + k_{36}Clb5Sic1(t) - \frac{k_{39}}{V_{\text{nuc}}(t)}Clb5(t)\cdot Sic1p(t) + k_{38}Clb5Sic1p(t)$$ (D.10.2)

$$\frac{dClb5Sic1}{dt} = \frac{k_{37}}{V_{\text{nuc}}(t)}Clb5(t)\cdot Sic1_{\text{nuc}}(t) - k_{36}Clb5Sic1(t) - \gamma_{42}(t)\cdot Clb5Sic1(t)$$ (D.10.3)

$$\frac{dClb6Sic1}{dt} = \frac{k_{31}}{V_{\text{nuc}}(t)}Clb6(t)\cdot Sic1_{\text{nuc}}(t) - k_{30}Clb6Sic1(t) - \gamma_{41}(t)\cdot Clb6Sic1(t)$$ (D.10.4)

$$\frac{dClb5Sic1p}{dt} = \gamma_{42}(t)\cdot Clb5Sic1(t) - k_{38}Clb5Sic1p(t) + \frac{k_{39}}{V_{\text{nuc}}(t)}Clb5(t)\cdot Sic1p(t)$$ (D.10.5)

$$\frac{dClb6Sic1p}{dt} = \gamma_{41}(t)\cdot Clb6Sic1(t) - k_{32}Clb6Sic1p(t) + \frac{k_{33}}{V_{\text{nuc}}(t)}Clb6(t)\cdot Sic1p(t)$$ (D.10.6)

$$\frac{dSic1_{\text{nuc}}}{dt} = k_{36}Clb5Sic1(t) - \frac{k_{37}}{V_{\text{nuc}}(t)}Sic1_{\text{nuc}}(t)\cdot Clb5(t) + k_{30}Clb6Sic1(t) - \frac{k_{31}}{V_{\text{nuc}}(t)}Sic1_{\text{nuc}}(t)\cdot Clb6(t) - \gamma_{40}(t)\cdot Sic1_{\text{nuc}}(t)$$ (D.10.7)

$$\frac{dSic1p}{dt} = k_{38}Clb5Sic1p(t) - \frac{k_{39}}{V_{\text{nuc}}(t)}Clb5(t)\cdot Sic1p(t) + k_{32}Clb6Sic1p(t) - \frac{k_{33}}{V_{\text{nuc}}(t)}Clb6(t)\cdot Sic1p(t) + \gamma_{40}(t)\cdot Sic1_{\text{nuc}}(t) - k_{43}Sic1p(t)$$ (D.10.8)

The formation of complexes Clb6Sic1 (rate constants k_{30}, k_{31}), Clb5Sic1 (rate constants k_{36}, k_{37}) and dissociation of complexes Clb6Sic1-P (rate constants k_{32}, k_{33}), Clb5Sic1-P (rate constants k_{38}, k_{39}) are modeled by standard mass-action law. Parameters k_{28} and k_{34} refer to the clearance rate for Clb6 and Clb5, respectively, k_{43} is the diffusion coefficient of Sic1-P out of the nucleus and k_{29} and k_{35} are the production rates obeying the rules for proper and weak activation mentioned in Supplementary Note 9, formally stated by the following equations:

$$k_{29} = \begin{cases} k_{29\mathrm{H}} & \text{if} \quad \left(\chi_{\{\mathrm{Nrm1}<\Theta_{\mathrm{Nrm1}}\}}(t)a^{\mathrm{C}}(t) + \chi_{\{\mathrm{Nrm1}\geq\Theta_{26}\}}(t)a^{\mathrm{A}}(t)\right)N_{\mathrm{C}} \geq \beta_{\mathrm{Clb6}} \\ k_{29\mathrm{L}} & \text{if} \quad (t > \Delta_{\mathrm{Clb6}}) \quad AND \quad \left(\left(\chi_{\{\mathrm{Nrm1}<\Theta_{\mathrm{Nrm1}}\}}(t)a^{\mathrm{C}}(t) + \chi_{\{\mathrm{Nrm1}\geq\Theta_{\mathrm{Nrm1}}\}}(t)a^{\mathrm{A}}(t)\right)N_{\mathrm{C}} < \beta_{\mathrm{Clb6}}\right) \\ 0 & \text{otherwise} \end{cases} \quad (C.10.4)$$

$$k_{35} = \begin{cases} k_{35\mathrm{H}} & \text{if} \quad \chi_{\{\mathrm{Nrm1}<\Theta_{\mathrm{Nrm1}}\}}(t)a^{\mathrm{B}}(t) \cdot N_{\mathrm{B}} \geq \beta_{\mathrm{Clb5}} \\ k_{35\mathrm{L}} & \text{if} \quad (t > \Delta_{\mathrm{Clb5}}) \quad AND \quad \left(\chi_{\{\mathrm{Nrm1}<\Theta_{\mathrm{Nrm1}}\}}(t)a^{\mathrm{B}}(t) \cdot N_{\mathrm{B}} < \beta_{\mathrm{Clb5}}\right) \\ 0 & \text{otherwise} \end{cases} \quad (C.10.5)$$

Supplementary Note 11

Extension of the model to the α-factor-induced cell cycle arrest

Biology. Haploid yeast cells treated with mating pheromones synchronize their cell cycle in G_1 and undergo morphological changes that prepare them to mating, a process that requires transcriptional remodeling, chemotropism and cell fusion [73]. Cell cycle arrest is reversible, so that cells can re-enter the cycle upon pheromone removal. Cln-Cdk1-specific inhibition plays a major role in mating-factor-mediated G_1 arrest. Notably after mating factor exposure, Far1 inhibitory activity towards Cln1,2 proteins increases following phosphorylation and further Far1 is synthesized [49,74]. Two major properties presented by the system are hysteresis and feed-forward [20,75].

Reconstruction. The α-factor signaling pathway is not explicitly represented in the mathematical model, but its effects on Far1 dynamics are represented by instantaneous modifications of the corresponding equations. More in details, the α-factor promotes Far1 production and phosphorylation. A further approximation is that Far1 phosphorylation occurs by means of a faster dynamics so that, as soon as the α-factor is added, the amount of phosphorylated Far1 (Far1-P, namely) can be considered as a proper fraction of

the total Far1. Both Far1 and Far1-P keep binding to Cln3, whilst only the activated form Far1-P promotes Cln1 and Cln2 degradation [74]. A negative feedback also occurs, since Cln1 and Cln2 both promote the degradation of Far1-P. As a matter of fact, when the α-factor is added during the early G_1-phase, and Cln1, Cln2 are not still accumulated, the α-factor is able to stop the cycle and forbid the onset of the budded phase by persistently inhibiting Cln3 activity; on the other hand, if the α-factor is added in the late G_1-phase, when Cln1, Cln2 have already been accumulated, the commitment to the budded phase is delayed but not blocked. All these assumptions are coherent with [20,75].

<u>*Computational representation.*</u> Let t_α be time instant when the α-factor is added. Then, for $t \geq t_\alpha$ Far1 and Cln3Far1 dynamics (i.e. eqs.(D.5.2-3)) are substituted by the dynamics of $Far1_{tot}$ and $Cln3Far1_{tot}$, obeying the constraints:

$Far1_{tot}$ = Far1 + Far1-P $Cln3Far1_{tot}$ = Cln3Far1 + Cln3Far1-P (C.11.1)

Both dynamics are below reported (see also Fig.2A):

$$\frac{dFar1_{\text{tot}}}{dt} = -\frac{k_7}{V_{\text{nuc}}(t)}Cln3_{\text{nuc}}(t)\cdot Far1_{\text{tot}}(t) + k_6Cln3Far1_{\text{tot}}(t) - k_{10}\frac{\left(\frac{Cln3_{\text{nuc}}(t)}{Cln3Far1_{\text{tot}}(t)}\right)^{n_{10}}}{1+\left(\frac{Cln3_{\text{nuc}}(t)}{Cln3Far1_{\text{tot}}(t)}\right)^{n_{10}}}Far1(t)$$
$$+ d_0(\alpha)V_{\text{nuc}}(t) - d_2Far1P(t) - \frac{d_4}{V_{\text{nuc}}(t)}\big(Cln1(t)+Cln2(t)\big)\cdot Far1P(t) \quad \text{(D.11.1)}$$

$$\frac{dCln3Far1_{\text{tot}}}{dt} = \frac{k_7}{V_{\text{nuc}}(t)}Cln3_{\text{nuc}}(t)\cdot Far1_{\text{tot}}(t) - k_6Cln3Far1_{\text{tot}}(t) \quad \text{(D.11.2)}$$

where the same rate constants (k_6, k_7) refer to complexes Cln3Far1 and Cln3Far1-P formation/disaggregation; not-phosphorylated-Far1 degradation is still controlled by the interplay between free nuclear Cln3 and its inhibited forms given by $Cln3Far1_{tot}$, whilst phosphorylated-Far1 degradation occurs according to a pair of possibilities: besides a linear clearance rate (with rate constant d_2), there is a Cln1,2-dependent degradation, last term in (D.11.1); $d_0(\alpha)$ is the α-factor induced Far1 production rate, whose value is modulated according to the α-factor concentration, see (C.11.3) below.

Nuclear Cln3 dynamics is straightforwardly modified from eq.(D.5.1) as follows:

$$\frac{dCln3_{\text{nuc}}}{dt} = k_5Cln3Ydj1_{\text{nuc}}(t) - \frac{k_7}{V_{\text{nuc}}(t)}Cln3_{\text{nuc}}(t)\cdot Far1_{\text{tot}}(t)$$
$$+ k_6Cln3Far1_{\text{tot}}(t) - k_9Cln3_{\text{nuc}}(t) + k_8Cln3_{\text{cyt}}(t) \quad \text{(D.11.3)}$$

Due to the faster dynamics of Far1 phosphorylation, Far1-P and Far1 can be modeled as instantaneous fractions of $Far1_{tot}$:

$$Far1P(t) = \frac{d_1(\alpha)}{d_1(\alpha)+d_3} Far1_{\mathrm{tot}}, \qquad Far1(t) = \frac{d_3}{d_1(\alpha)+d_3} Far1_{\mathrm{tot}} \tag{C.11.2}$$

with $d_1(\alpha)$ the α-factor induced phosphorylation coefficient for Far1, and d_3 the dephosphorylation coefficient. Finally, the equations of Cln1 and Cln2 dynamics (eqs.(D.9.3) and (D.9.1), respectively) are modified as follows, to properly account for the inhibitory action of Far1 (last term in both equations):

$$\frac{dCln1}{dt} = k_{27}V_{\mathrm{nuc}}(t) - k_{26}Cln1(t) - \frac{d_5}{V_{\mathrm{nuc}}(t)} Cln1(t) \cdot Far1P(t) \tag{D.11.4}$$

$$\frac{dCln2}{dt} = k_{23}V_{\mathrm{nuc}}(t) - k_{22}Cln2(t) - \frac{d_5}{V_{\mathrm{nuc}}(t)} Cln2(t) \cdot Far1P(t) \tag{D.11.5}$$

The way the amount of administered α-factor α modifies parameters $d_0(\alpha)$ and $d_1(\alpha)$ (the ones directly related to it) has been modeled by means of a Michaelis-Menten saturating function:

$$d_i = d_i(\alpha) = M_i \frac{\alpha}{\alpha + \mu_i}, \qquad i = 0, 1 \tag{C.11.3}$$

Supplementary Note 12

Model parameter setting

The molecular model is inherently stochastic, because of the chosen machinery adopted to set the activation times of *CLN1*, *CLN2*, *CLB5*, *CLB6* and *NRM1* (see Supplementary Note 9). As a matter of fact, given the same feasible set for all the model parameters, any two runs of the model would provide different outputs. Moreover, inter-cell variability has been considered, in order to provide simulations of populations of cells. To this end, any single cell model parameters are supposed to be sampled from independent log-normal distributions, whose average values and coefficients of variation have been manually curated in order to replicate the many experiments the model is able to account for.

Initial conditions for a newborn cell are set according to the Supplementary Table 1. The initial conditions for the growth model are given in terms of feasible ranges for proteins and ribosomes, including the case of very small cells (with $P(0)$ = 1.0e10aa). Initial Cln3 is supposed to be all in the ER, therefore, according to (C.4.1):

$$Cln3_{ER}(0) = Cln3_{tot}(0) = k_0 * P(0) \tag{C.12.1}$$

Far1, Swi6, Swi4 and Mbp1 are supposed to be given to newborn cells free of any binding, as well as Whi5 and Sic1 which are also supposed to have no phosphorylations.

All but the growth initial conditions (i.e. $P(0)$ and $R(0)$) are given to model a newborn cell with no uncertainties. On the other hand, different variabilities in the initial protein and ribosome content can be exploited to replicate different experimental frameworks.

Supplementary Note 13

In silico experimental procedures

The proposed model has been exploited to replicate a wide range of experiments involving single cells and populations of cells. As for the single cells simulations, the model parameters have to be thought as fixed to the average values provided by the Supplementary Tables 1-7. The growth parameters, whose average values are not univocally given by the Supplementary Table 2, are shown in Supplementary Table 9 with reference to their corresponding figures in the main text. Notice that, according to Supplementary Tables 2 and 9, the exponential growth condition (C.2.3) is satisfied, providing an exponential growth rate equal to 0.0071min^{-1}.

In case of simulations of populations of cells, any cell of the population is supposed to be given a set of model parameter values as sampled from independent log-normal distributions with average values taken from Supplementary Tables 2-7. As far as the coefficient of variations (CV) (unless explicitly specified in the Tables, as for the activation orders and the weak activation times in Supplementary Table 5), all but parameter k_{3M} are supposed to share the same CV, which may range between 5% and 40%. On the contrary, k_{3M} is sampled from a log-normal distribution with a large variance (CV = 100%), since a very high cell-to-cell variability has been supposed for Cln3 translocation into the cytoplasm (Supplementary Note 4). Also the initial growth conditions vary (both in average value and in CV) according to different population frameworks.
As far as mutant cases, the following experimental procedures have been taken into account:

- different functional/decoy phosphorylation schemes: there no modification in the general setting of the model parameters

- *cln3Δ*: parameter k_0 in (C.4.1) is set equal to zero in order to obtain complete absence of the cyclin
- *cln2Δ*: parameters k_{23H} and k_{23L} in (C.9.9) are set equal to zero in order to obtain complete absence of the cyclin
- *whi5Δ*: the total amount of Whi5 in (C.7.18) is set equal to zero in order to obtain complete absence of the inhibitor
- OCln3 (e.g. 6xCln3): the average value of parameter k_0 in (C.4.1) is increased (e.g. 6-fold the nominal value) in order to show overexpression
- OCln2 (e.g. 6xCln2): the average value of parameters k_{23H} and k_{23L} in (C.9.9) are increased (e.g. 6-fold the nominal value) in order to show overexpression
- extra-SBF: the extra SBF binding sites are modeled by increasing the number of SBF-affine genes (N_A = 166 instead of 136, N_C = 44 instead of 36). As a matter of fact also the average values of the activation order of the SBF-affine genes have been modified as follows: $\langle\beta_{Cln1}\rangle = 9$, $\langle\beta_{Cln2}\rangle = 45$, $\langle\beta_{Nrm1}\rangle = 125$, $\langle\beta_{Clb5}\rangle = 24$
- *ydj1Δ*: besides the setting of parameter $k_1 = 0$, in order to prevent the chaperon-dependent diffusion, we have supposed that this mutant provides a population clustered in two classes, one growing at a much smaller growth rate than the other. Such a fact is modeled by properly varying the growth parameters K_2 and ρ. By denoting the slow grow population with *ydj1Δ-a* and the other with *ydj1Δ-b*, the corresponding growth rates coming from (C.1.4) are 0.0031min^{-1} and 0.0063min^{-1}, respectively. Moreover, both the clusters of cells increase the average value of the weak activation time of Cln1 and Cln2, setting it equal to 150min (with standard deviation of 50min for *ydj1Δ-a*) and equal to 60min (with standard deviation of 20min for *ydj1Δ-b*). See Supplementary Note 17 for more motivation and more details.

Supplementary Table 10 summarizes on the population parameters, and refers to the main text figures. Among them, Fig.3A is made of a pair of distinct populations. One population is made of average (m) cells, the other is made of extra-small (xs) cells.

Supplementary Note 14

Growth rate standardized duration of the G_1-phase (λT_{G1})

The growth-rate-standardized duration of the G_1 phase is defined as the product of the exponential growth rate λ times the length of the G_1 phase: λT_{G1}. As in [19], it can be computed for any cell by suitably exploiting the relationship between initial and critical size, $P(0)$ and P_s, respectively:

$$P_s = P(0)*\exp(\lambda T_{G1}) \quad \Rightarrow \quad \lambda T_{G1} = \ln(P_s) - \ln(P(0)) \qquad \text{(C.14.1)}$$

Fig. 3A reports a set of experimental findings taken from [19] (in the x-axis the size is normalized to the average size at budding of the population) of this relation which presents a large noise and shows a tendency of larger newborn cells to have a shorter λT_{G1} value. Similarly to the experimental setting of [19], simulated cells are sampled from two distinct populations, one referring to average size initial conditions, the other referring to extrasmall size initial conditions. Details can be found in Supplementary Table 10. As reported in Fig. 3A the simulated values (red) closely superimpose with experimental findings (blue).

Supplementary Note 15

λT_{G1}-vs-$P(0)$ as an input-output relationship provided by the model

The use of the proposed model of the G_1/S transition allows to compute P_s as a function of $P(0)$, thus exploiting (C.14.1) as a numerical input-output relationship between initial protein content and λT_{G1}. Even though it is not possible to determine an analytical solution of (C.14.1), it becomes of interest to determine from a statistical analysis of its simulated findings the best fitting mathematical relation between $P(0)$ and λT_{G1}. In order to do so, simulations were performed for populations of 50 cells for any given initial protein content, which varied from 1.0e10 aa to 3.2e10 aa, with incremental steps of 0.1e10 aa. The initial ribosome content was fixed to $\rho P(0)$. For each initial condition the 50 simulations have been run by sampling the model parameters according to the average values and the CVs provided in Supplementary Table 10. Fig. 3B reports in grey the 75% quantile region of the simulated findings, while the red line indicates the resulting average λT_{G1} value. A second set of simulations was run considering the standard average cells (i.e. cells with fixed

average parameters) differing only for their initial protein content, which, as in the previous case, assumed values in [1.0e10, 3.2e10] aa with incremental steps of 0.1e10aa. The standard value curve so obtained is plotted in Fig. 3B as a blue line, and is exploited as a model to determine the input-output relationship in terms of the best hyperbola fitting. Indeed, by setting

$$\lambda T_{G1} = a/(P(0)\text{-}b) \tag{C.15.1}$$

the minimum square error provides a = 3.4.e9 aa and b = 4.1e9 aa. The goodness of fit is shown in Fig. 3E. In this way, the analytically relationship (C.15.1) can be adopted to approximately compute λT_{G1} for any given value of initial cell size $P(0)$ without running any simulations.

Supplementary Note 16

Analysis of λT_{G1} variability

Data reported in Table 1, present the decomposition of G_1 variability (first column) in terms of deterministic size control (second column) and a residual attributable to molecular noise (third column). The assumption is that the growth-rate-standardized time in G_1, λT_{G1}, can be decomposed in the sum of a suitable deterministic function of the initial protein content, name it $h(P(0))$, plus a random variable η. The chosen function for $h(P(0))$ is the hyperbola described in Fig. 3E, encouraged by the good fitting results with the standard value curve. Therefore, the molecular noise is given by:

$$\eta = \lambda T_{G1} - h(P(0)) \tag{C.16.1}$$

The table is computed for different cell populations. The WT case refers to subset of 90 cells sampled from the average size initial conditions in Fig. 3A. The other cases refer to cell populations built according to the same meta-parameters, obtained by running overexpressions of cyclins Cln2 and Cln3. The size dependent/independent variability (columns 2 and 3, respectively) is reported in terms of the coefficients of variation of the components; the overall variability (column 1) is the square root of the sum of the squares of the two noise components. Simulated data are compared to experimental data coming from [19]. Numbers in parenthesis refer to the percentage of the overall variability in terms of size-dependent or size-independent terms.

As a final remark, we focus our attention on the T_{G1} as given by the sum of T_1 and T_2, being the variability largely found in T_1 [19]. Indeed, simulations carried out by the proposed mathematical model of the G_1/S transition support such a statement. To this end, we will generalize λT_{G1} to the growth-rate-standardized duration of the T_2 period, λT_2, which, analogously to λT_{G1}, is achieved by means of size measurements at the end of Timer T_1, P_1, and at the onset of the critical size, P_s:

$$P_s = P_1 * \exp(\lambda T_2) \quad \Rightarrow \quad \lambda T_2 = \ln(P_s) - \ln(P_1) \tag{C.16.2}$$

Fig. 3F reports λT_2 as a function (computed by simulations) of cyclin Cln2 production rate, showing a very low correlation of λT_2 to the *CLN2* dosage. The blue line refers to single cell simulations of 18 average cells (i.e. cells with fixed average parameters) differing only for cyclin Cln2 production rate (parameter k_{23H}) with values in the range [1.0e15, 9.5e15] mo^{-1} L min^{-1} with an incremental step 0.5e15 mo^{-1} L min^{-1}. For each of these conditions, populations of 20 cells have been simulated, simulations have been run by sampling the model parameters according to the average values and the CVs provided in Supplementary Table 10. The red line refers to the average value for the product λT_{G1}. The grey region is the 75% quantile region.

Supplementary Note 17

Linear growth rate (α) and critical volume (V_s)

In this Section we focus our attention on the plots obtained in Fig.4 of [19], where clouds of scattered points are reported in a 'V_s-*versus*-α' plot. Each point refers to a single cell evolution during the G_1/S transition, with 'V_s' denoting the *critical volume* measured at the end of Timer T_1 and 'α' denoting the *linear growth rate*, computed as the average increase rate in volume during the T_1 period:

$$\alpha = (V_s - V_i) / T_1 \tag{C.17.1}$$

'V_i' stands for the volume at birth. Similar plots can be drawn according to a population of simulated cells.

In case of models providing cells in exponential growth a grid can be considered, whose curves refer to cells growing at fixed T_1 length and fixed V_i volumes. To this end, we write

both V_s and α as functions of the pair (T_1,V_i). As far as V_s, the required relationship readily comes from the exponential growth hypothesis. Indeed, by denoting with λ the exponential growth, it is:

$$V_s = V_i * \exp(\lambda T_1) \qquad \text{(C.17.2)}$$

By substituting eq.(C.17.2) in eq.(C.17.1), the second required relationship comes:

$$\alpha = V_i\,(\exp(\lambda T_1) - 1) / T_1 \qquad \text{(C.17.3)}$$

For a given growth rate λ, by fixing T_1, eqs.(C.17.2-3) draw the curves at fixed T_1 length, whilst by fixing V_i, eqs.(C.17.2-3) draw the curves at fixed initial volume V_i. Supplementary Fig. 10A reports these two classes of curves for the case of $\lambda = 0.0063\text{min}^{-1}$. That means, according to the exponential growth rate condition (C.2.4): K_2 = 500 aa rib^{-1} min^{-1}, ρ = 1.32e-5 rib aa^{-1}, D_2 = 3000 min, in terms of the growth model parameters. The black lines refer to the curves at fixed Timer T_1 length, drawn for V_i greater than 1 μm^3: the black numbers over the lines refer to T_1 in minutes. The red lines refer to the curves at fixed initial volume V_i, drawn for T_1 greater than 1min: the red numbers over the lines refer to V_i in μm^3.
These grids allow to visually capture the T_1 length and the initial volume V_i for a point (i.e. a cell) in the 'V_s-*versus*-α' plot. For instance, consider the set of WT cells reported in the panels of Fig.4 in [19]. According to Table S6 in Suppl. Material of the same reference [19], it comes that these WT cells share an average critical volume of 36.1 μm^3, an average initial volume of 24.8 μm^3 and an average T_1 length of 59.6 min. By assuming that these cells are in exponential growth, an estimate of the average λ is given by inverting eq.(C.17.2) by means of the aforementioned average values, thus providing $\lambda = 0.0063\ \text{min}^{-1}$. For the sake of simplification, all these cells are drawn in Supplementary Fig. 10B on the same grid of Supplementary Fig. 10A (to be precise, each cell is supposed to follow an exponential growth slightly different than any other, according to cell-cell variability, thus each cell should refer to its own exponential growth, according to which a proper grid should be drawn). By visual inspection of Supplementary Fig. 10B it readily comes that quite all these WT cells have a T_1 length smaller than 150 min, and the few ones exceeding this value share a very small initial volume, within 5 and 10 μm^3.

Besides the help in interpreting data by visual inspection, the grid allows also to design wet or in silico experiments. For instance, let us consider the experiment of *ydj1Δ* in Fig.4e of

[19]. By assuming that also this experiment involves exponential growth, the average data of Table S6 in Suppl. Material of [19] tell us that the estimated λ is 0.0031 min^{-1}. That means, in terms of the growth model parameters: K_2 = 330 aa rib^{-1} min^{-1}, ρ = 1.0e-5 rib aa^{-1}, D_2 = 3000 min. If we draw these points on a grid with λ = 0.0031 min^{-1} we obtain the picture in Supplementary Fig. 10C. Within the approximation that all cells share the same exponential growth (i.e. they share the same grid) it seems that only a subset of the points is appropriate to the grid, the rest apparently requiring a negative T_1 length to be consistent. This fact could be explained by assuming to cluster the cells into two sets: one set that reacts to the chaperon deletion by lowering its exponential growth (the points within the grid in Supplementary Fig. 10C, over the line at T_1 = 1 min); the other one that does not substantially modifies the exponential growth (the points outside the grid, below the line at T_1 = 1 min). Indeed, if we draw the experimental points of *ydj1Δ* from [19] on the same grid of λ is 0.0063 min^{-1}, Supplementary Fig. 10D, they are all included within reasonable values of T_1.

This clustering may suggest the road to set up two distinct *in silico* experiments to replicate this heterogeneous set of data. Let us focus our attention on the first cluster of points, the ones that fit the grid at λ = 0.0031 min^{-1}, thus suggesting a lowering of the growth rate. A fact that appears from the grid in Supplementary Fig. 10C is that these cells share a high average T_1 length, with a very wide variation, spanning from tens to hundreds of minutes. To replicate such a fact it has been assumed to increase (w.r.t. the WT case) the average value of the random times of activation of cyclins Cln1,2 (now strongly involved in the G_1/S transition because of Cln3 leaky transport into the nucleus without the chaperone). The great variability of the points in the grid is replicated by means of a great variability of such random activation times. In summary, the random times of activation of cyclins Cln1,2 change into a lognormal distribution with average value of 150 min and standard deviation of 50 min (instead of a lognormal distribution with average value of 30 min and standard deviation of 10 min, see Supplementary Table 5). Supplementary Fig. 10E shows this first cluster of simulated cells (blue dots) together with the ones from [19].

The second cluster of cells has been simulated according to a different setting of the exponential growth, equal to the WT case, so that they could be drawn on the grid of Supplementary Fig. 10A. As a matter of fact, this second cluster of points shares a T_1 length within 50 and 250 min, with an initial volume V_i within 10 and 35 μm^3, that means, an initial protein content within 0.7e10 and 2.5e10 aa. In order to simulate cells that could

be compared with a good fit with these ones related to the second cluster, we chose an average initial protein content of 1.6e10 aa (with a CV = 18%) and fix the average random times of activation of Cln1,2 at 60 min, with a standard deviation of 20 min. Simulated cells are reported on Supplementary Fig. 10F (blue), compared to the experimental cells from [19], in green. As it clearly appears, simulated data very good match with the second cluster. Figs. 4E-F compare the experimental vs simulated cells of *ydj1Δ*, keeping the same proportion between the two cluster (the first cluster is about 3-fold bigger than the second). The clusters are highlighted by ovals.

Supplementary Note 18

α-vs-V_s as an input-output relationship provided by the model

The apparent linear correlation, experimentally observed between the critical volume (V_s) and the linear growth rate (α), presented in [62] as a proof that the growth rate sets the critical cell size, is obtained also by means of simulations of our model of the G_1/S transition (see WT simulations in Fig. 4B), that considers the critical cell size as the emergent property of the network presented in Fig. 1A, for which increase in mass of each cell follows an exponential growth.

In the following it will be shown how to analytically derive from our model such an '*almost*' linear relationship between V_s and α. To this end we consider cells with fixed average parameters (Supplementary Tables 2-7) differing only for their initial protein content $P(0)$, with values in the 1.0e10aa to 3.2e10aa range, with incremental steps of 0.1e10 aa. The initial ribosome content is fixed to $\rho P(0)$. This set of cells is the same used to compute the blue line in Fig. 3B. Differently from Fig. 3B, however, in the present case we are interested in volumes (instead of protein content) and in Timer T_1 length (instead of phase G_1). The black line in Fig. 4E reports the α-vs-V_s relationship, numerically computed by our simulation data.

The α-vs-V_s relationship can be inferred from our model starting from the search for the best hyperbola fitting the λT_1-vs-V_i curve, where λT_1 is the growth-rate-standardized duration of Timer T_1, defined by:

$$\lambda T_1 = \log(P_1) - \log(P(0)) \qquad \text{(C.18.1)}$$

with P_1 the cell size at the end of T_1. This best fitting computation is similar to the one carried out in Supplementary Note 15 for the best hyperbola fitting the λT_{G1}-vs-$P(0)$ curve. After computation, we obtain:

$$\lambda T_1 = a'/(V_i - b') \qquad \text{with} \quad a' = 3.213\mu m^3, \qquad b' = 7.815\ \mu m^3 \tag{C.18.2}$$

Since cell volume is proportional to cell size in our model, eq.(C.18.1) can be also written by explicitly involving volumes, so that:

$$\lambda T_1 = \log(V_s) - \log(V_i) \qquad \text{that means} \qquad V_s = V_i{*}\exp(\lambda T_1) \tag{C.18.3}$$

Thus, by substituting (C.18.2) in the second equation of (C.18.3) we can write the initial volume V_i as an invertible function of V_s: $V_i = f_1(V_s)$. Unfortunately there is not an analytical formulation for $f_1(V_s)$. Analogously we can derive from (C.18.2) the length of T_1 as a function of V_s:

$$T_1 = a'/(\lambda{*}(V_i - b')) = a'/(\lambda{*}(f_1(V_s) - b')) = f_2(V_s) \tag{C.18.4}$$

Finally, by substituting $V_i = f_1(V_s)$ and $T_1 = f_2(V_s)$ into the definition of the linear rate of growth (C.17.1) we obtain the V_s versus α relationship drawn in Fig. 4E (red line), which very god fits with the numerical data.

Supplementary Note 19

Sensitivity analysis

Intrinsic consistency and robustness of a model needs to be ascertained against variations in parameter values. Tested parameters were grouped in four sets: Cln3 production and nuclear import (parameters in Supplementary Table 3); G_1/S regulon activation (parameters in Supplementary Table 4 except for $Swi6_{tot}$, $Swi4_{tot}$, $Mbp1_{tot}$, $Whi5_{tot}$); Cln/Clb function (parameters in Supplementary Table 6); Sic1 function (parameters in Supplementary Table 7 except for $Sic1_{tot}$). Taking the standard value as 1, each parameter was either increased (up to 81 fold the standard value, step 3) or decreased (down to 1/81 fold the standard value, step 1/3). The impact of each of these changes in parameter values was tested on four significative outputs: N, the Hill coefficient of the Hill function that best fits the G_1/S regulon activation curve, the length of the T_1 and T_2 period and the critical size P_s. Coefficient N will be referred to in the sequel as the *coherence* of the

activation. The sensitivity analysis was done by varying each parameter individually, associating it to the run of a single cell deterministic simulation, with the proper order of activation and the weak activation times fixed to their average values in Supplementary Table 5.

Supplementary Figs. 12-15 report the results according to the different sets of input and output. The outputs have been normalized with respect to cell values obtained using the standard parameter set. The scale is logarithmic to better appreciate the positive/negative variation of the outputs. The range of the outputs has been fixed for quite all the panel from 0.1-fold to 20-fold the average cell value, except for one panel in Supplementary Fig. 14 that required a wider range from 0.01-fold to 20-fold. When a point corresponding to a large parameter variation is missing, it means that the output cannot be calculated. This may happen because, for instance, time periods T_1 or T_2 never end (because nuclear Whi5 or nuclear Sic1 never exit the nucleus), or because Timer T_2 ends before Timer T_1. We refer to these cases as *unfeasible*.

Supplementary Note 20

Sensitivity analysis for the coherence of activation (Supplementary Figure 12)

Cln3 production and nuclear import. Within this group of parameters, the most significant increase in N was obtained by lowering k_2, i.e. the Cln3 diffusion coefficient from cytoplasm into ER or increasing k_9, i.e. the Cln3 diffusion coefficient from nucleus into cytoplasm. A minor increase in coherence (about 20%) was obtained by lowering the rate constant for Cln3-Ydj1 diffusion from the cytoplasm into the nucleus when cytoplasmic Cln3-Ydj1 is low (k_{4L}). It should be noted that at the beginning of the cycle, wild type cycling cells have an initial amount of $Cln3_{ER}$ high enough to exceed such a threshold. Therefore such a process plays an effective role especially in extra-small or starved cells. Similar results are obtained by lowering the rate constant for dissociation of the nuclear Cln3-Ydj1 complex (k_5), or increasing k_3, i.e., the rate constant that drives Cln3 back to the cytoplasm).

G_1/S regulon activation. A more than 9-fold increase/decrease in several parameters in this group resulted in *unfeasible* cells. These parameters include Θ_{20}, k_{19}, k_{20} and $\mathbf{k_{21}}$. In

the feasible cases, the parameters whose alteration increased significantly the value of N are Θ_{20} and n_{20}, while the strongest decrease was obtained by increasing k_{20}. These parameters are related to Swi6 and Whi5 phosphorylation rate effected by the Cln-Cdk1 complexes. Reductions of coherence N (of a smaller entity, about 30-40%) occur when the formation of complex Swi6Swi4Whi5 is weakened (e.g. by suitably varying the on/off coefficient of reaction (R.7.3), parameters k_{13}, k_{14}) in favor of a larger amount of Swi6Swi4, or when the binding of Swi6Swi4 is enhanced (parameter k_{18}). In both cases the control role played by Whi5 is reduced, since the binding of the transcription factor free of its inhibitor will be favored, thus allowing a direct activation without the chain of phosphorylations required to get rid of Whi5. The result is a shorter T_1 length with a smaller role of the positive feedback of Cln1 + Cln2. Finally a reduction of the Hill coefficient (about 20-40%) occurs also when the formation of complex Swi6Mbp1 is weakened (on/off coefficients of reaction (R.7.2), parameters k_{15}, k_{16}), as well as when the binding coefficient of Swi6Mbp1 is reduced (parameter k_{17}). In all cases, however, there is no reduction of Timer T_1. Indeed, from one hand, a smaller presence of Swi6Mbp1 enhances the role of Whi5 in class C genes, allowing to increase the synchronization due to the phosphorylation mechanism; on the other hand a smaller presence of Swi6Mbp1 delays the activation of class B genes, thus preventing an appreciable increase in Timer T_1 length (see also Supplementary Fig. 13).

Cln/Clb function. Increasing parameter k_{35H} more than 27-fold made cells *unfeasible*. Only a strong increase (at least 27x) in k_{23H} (the proper production rate of Cln2) and k_{27H} (the proper production rate of Cln1) significantly increased (about 2-fold) the value of N. Alteration in other parameters had no effect.

Sic1 function. None of the parameters in this group had any effect on N.

Supplementary Note 21

Sensitivity analysis for the length of Timer T_1 (Supplementary Figure 13)

Overall, results obtained for the time interval T_1 were similar to those obtained for N

Cln3 production and nuclear import. Within this group of parameters, the most significant increase in T_1 was obtained by lowering k_2, i.e. the Cln3 diffusion coefficient from cytoplasm into ER or increasing k_9, i.e. the Cln3 diffusion coefficient from nucleus into cytoplasm. Alterations in all other parameters had either a minor or no effect.

G_1/S regulon activation. A large increase/decrease in several parameters in this group provided *unfeasible* cases. These parameters include Θ_{20}, k_{19}, k_{20} and $\mathbf{k_{21}}$. In the *feasible* cases, the length of Timer T_1 increased maximally by increasing parameters Θ_{20}, n_{20}, and decreasing parameter k_{20} (parameters related to Swi6 and Whi5 phosphorylation, effected by the Cln-Cdk1 complexes). The opposite effect occurs when decreasing Θ_{20}, n_{20}, and increasing k_{20}.

Cln/Clb function. Increasing parameter k_{35H} more than 27-fold provided unfeasible cases. Only the SBF/MBF-mediated production rate of Cln1 (parameter k_{27H}) had about 20% effect of variation on T_1 length.

Sic1 function. None of the parameters in this group had any effect on T_1.

Supplementary Note 22

Sensitivity analysis for the length of Timer T_2 (Supplementary Figure 14)

Cln3 production and nuclear import. A sizable, but modest (about 20% max) effect on the length of T_2 was observed for the Cln3 diffusion coefficient from cytoplasm into ER (k_2) and the rate constant for Cln3 exit from the nucleus into the cytoplasm (k_9), alteration of other parameters in this group having little or no effect.

G_1/S regulon activation. A more than 9 fold increase/decrease in several parameters in this group provided unfeasible cases as described above. These parameters include Θ_{20}, k_{19}, k_{20} and $\mathbf{k_{21}}$. This latter, controlling the diffusion of nuclear phosphorylated Whi5 into the cytoplasm, showed the most dramatic effect in simulated cells able to complete the G_1/S transition. Finally, a more than 9-fold variation (increase in k_{16} and decrease in k_{15}, k_{17}), caused the T_2 period to almost double in length.

Cln/Clb function. In this group, the largest effect on the length of T_2 are caused by parameters involved in the proper production rate of Clb5 and Clb6 (k_{35H} and k_{29H}, respectively), in the proper production rate of Nrm1 (k_{25H}), and in its ability to inhibit MBF (Θ_{Nrm1}). Increasing parameter k_{35H} more than 27-fold provided unfeasible cases.

Sic1 function. In this group decreasing the rate constant for the exit of phosphorylated Sic1 from the nucleus (k_{43}) had the strongest effect on T_2 length (more than 10-fold). Other parameters with a sizable effect include k_{31}, k_{36}, k_{37}, k_{41} and k_{42}.

Supplementary Note 23

Sensitivity analysis for the critical size P_S (Supplementary Figure 15)

Cln3 production and nuclear import. Two parameters in this group significantly affected the critical size P_s: Cln3 diffusion rate from cytoplasm into the ER (parameter k_2) and Cln3 diffusion rate from the nucleus into the cytoplasm (parameter k_9).

G_1/S regulon activation. A greater than 9-fold increase/decrease in several parameters in this group provided unfeasible cases as described above. These parameters include Θ_{20}, k_{19}, k_{20} and $\mathbf{k_{21}}$. Within the feasible cases the most dramatic effects on the variation of the critical size were caused by k_{20}, Θ_{20} and n_{20}.

Cln/Clb function. P_s is robust to alterations in parameters affecting synthesis and functionality of Clns and Clbs, except that a 81-fold increase in k_{35H} (the proper production rate of Clb5), provided unfeasible cases.

Sic1 function. A strong reduction in nuclear export of phosphorylated Sic1 (k_{43}) caused about a 2-fold increase in P_s.

Notice that variations in P_s, though correlated to variations in Timer T_1, are reduced in magnitude, reflecting the fact that the critical size P_s can be roughly represented as an exponential function of Timer T_1. Indeed, from (C.14.1):

$$P_s = P(0)*\exp(\lambda(T_1 + T_2)) \quad \text{(C.23.1)}$$

If we denote with T_1^δ, P_s^δ the T_1 length and P_s modification according to a given parameter variation, and assume to have no significant variation in T_2, then the relative variation in P_s ($r = P_s^\delta/P_s$) can be written in terms of the relative T_1 length variation ($q = T_1^\delta/T_1$):

$$r = P_s^\delta/P_s = P(0)*\exp(\lambda(T_1^\delta + T_2))/P_s = \exp(\lambda(q\text{-}1)T_1) \qquad \text{(C.23.2)}$$

Thus, in case of λ = 7.1e-3 min^{-1} and T_1 = 21 min, a strong relative variation in T_1 - like q = 10, or q = 0.1 - results in a smaller corresponding variation r for P_s (3.8 and 0.9, respectively).

Supplementary Note 24

Model complexity and simulation times

From a numerical viewpoint any single cell evolution related to our model requires the integration of a set of ordinary differential equations, describing synthesis, degradation, activity and sub-cellular localization of proteins and protein complexes, as well as the time evolution of the probabilities related to the DNA-bound transcriptional activators. On top of that, we provide actually a family of model, each element being identified by a suitable choice of Functional versus Decoy phosphorylation site configuration. Each Fu/De configuration shares the same number molecular players (13 different proteins, combining to form an overall number of 20 protein complexes, this number not accounting for the diversity provided by phosphorylations) and the same number of model parameters, 81. Instead, the dynamical state variables of the Ordinary Differential Equation systems associated to the model vary according to different Fu/De configurations. For instance, the 4/8 wild type case involves 264 ODEs, with most of the equations (237 out of 264) involved by the probability equations. Different kind of dynamics are involved and, as a consequence, different time scale are represented, thus providing a stiff ODE, whose numerical integration may be nontrivial.

For the ease of the reader, we have performed a statistical analysis of the computational costs related to a single cell simulation, see Supplementary Fig. 19. Panel A reports the distribution of the simulation times for 1000 wild type cells whose model parameters and initial conditions were randomly sampled from lognormal distributions with CV = 12%. Panel A shows that, except for very few outliers, most of the cells require less than 20 minutes (panel C shows that 992 cells out of 1000 require less than 10 minutes). In any

case, also accounting for the outliers, we have an average computational cost of 3.8 minutes per cell, meaning a time of about 18 hours for a population of 1000 cells. Of course these computations are strongly related to the kind of simulator (MatLab stiff ODE integrator, ode15s.m) and on the kind of machine the computations are run on. In any case, it is of worth noticing that the simulation length is not correlated to the G_1-phase length of the cell, as it appears from panels B and D.

Supplementary References

1 Mitchison, J. M. *The biology of the cell cycle.*, (Cambridge University Press, 1971).

2 Prescott, D. M. *Reproduction of eukaryotic cells*. (Academic Press, 1976).

3 Johnston, G. C., Pringle, J. R. & Hartwell, L. H. Coordination of growth with cell division in the yeast Saccharomyces cerevisiae. *Exp Cell Res* **105**, 79-98 (1977).

4 Alberghina, L. *et al.* Cell growth and cell cycle in Saccharomyces cerevisiae: basic regulatory design and protein-protein interaction network. *Biotechnol Adv* **30**, 52-72, doi:10.1016/j.biotechadv.2011.07.010 (2012).

5 Hartwell, L. H. & Unger, M. W. Unequal division in Saccharomyces cerevisiae and its implications for the control of cell division. *J Cell Biol* **75**, 422-435 (1977).

6 Lord, P. G. & Wheals, A. E. Variability in individual cell cycles of Saccharomyces cerevisiae. *J Cell Sci* **50**, 361-376 (1981).

7 Lord, P. G. & Wheals, A. E. Asymmetrical division of Saccharomyces cerevisiae. *J Bacteriol* **142**, 808-818 (1980).

8 Tyson, C. B., Lord, P. G. & Wheals, A. E. Dependency of size of Saccharomyces cerevisiae cells on growth rate. *J Bacteriol* **138**, 92-98 (1979).

9 Vanoni, M., Vai, M., Popolo, L. & Alberghina, L. Structural heterogeneity in populations of the budding yeast Saccharomyces cerevisiae. *J Bacteriol* **156**, 1282-1291 (1983).

10 Elliott, S. G. & McLaughlin, C. S. Rate of macromolecular synthesis through the cell cycle of the yeast Saccharomyces cerevisiae. *Proc Natl Acad Sci U S A* **75**, 4384-4388 (1978).

11 Klumpp, S., Scott, M., Pedersen, S. & Hwa, T. Molecular crowding limits translation and cell growth. *Proc Natl Acad Sci U S A* **110**, 16754-16759, doi:10.1073/pnas.1310377110 (2013).

12 Alberghina, L., Mariani, L. & Martegani, E. Cell cycle modelling. *Biosystems* **19**, 23-44 (1986).

13 Turner, J. J., Ewald, J. C. & Skotheim, J. M. Cell size control in yeast. *Curr Biol* **22**, R350-359, doi:10.1016/j.cub.2012.02.041 (2012).

14 Martegani, E., Popolo, L., Alberghina, L. & Sturani, E. Reduction of ribosome activity and synthesis of stable RNA in Neurospora crassa. *Biochim Biophys Acta* **610**, 318-330 (1980).

15 Nomura, M., Gourse, R. & Baughman, G. Regulation of the synthesis of ribosomes and ribosomal components. *Annu Rev Biochem* **53**, 75-117, doi:10.1146/annurev.bi.53.070184.000451 (1984).

16 Zinzalla, V., Stracka, D., Oppliger, W. & Hall, M. N. Activation of mTORC2 by association with the ribosome. *Cell* **144**, 757-768, doi:10.1016/j.cell.2011.02.014 (2011).

17 Cook, M. & Tyers, M. Size control goes global. *Curr Opin Biotechnol* **18**, 341-350, doi:S0958-1669(07)00088-2 [pii] 10.1016/j.copbio.2007.07.006 (2007).

18 Barberis, M., Klipp, E., Vanoni, M. & Alberghina, L. Cell size at S phase initiation: an emergent property of the G1/S network. *PLoS Comput Biol* **3**, e64, doi:06-PLCB-RA-0291R4 [pii] 10.1371/journal.pcbi.0030064 (2007).

19 Di Talia, S., Skotheim, J. M., Bean, J. M., Siggia, E. D. & Cross, F. R. The effects of molecular noise and size control on variability in the budding yeast cell cycle. *Nature* **448**, 947-951, doi:nature06072 [pii] 10.1038/nature06072 (2007).

20 Doncic, A., Falleur-Fettig, M. & Skotheim, J. M. Distinct interactions select and maintain a specific cell fate. *Mol Cell* **43**, 528-539, doi:10.1016/j.molcel.2011.06.025 (2011).

21 Costanzo, M. *et al.* CDK activity antagonizes Whi5, an inhibitor of G1/S transcription in yeast. *Cell* **117**, 899-913, doi:10.1016/j.cell.2004.05.024 (2004).

22 Hasan, M. M. *et al.* A comparative study of Whi5 and retinoblastoma proteins: from sequence and structure analysis to intracellular networks. *Front Physiol* **4**, 315, doi:10.3389/fphys.2013.00315 (2013).

23 Kõivomägi, M. *et al.* Cascades of multisite phosphorylation control Sic1 destruction at the onset of S phase. *Nature* **480**, 128-131, doi:10.1038/nature10560 (2011).

24 Brümmer, A., Salazar, C., Zinzalla, V., Alberghina, L. & Höfer, T. Mathematical modelling of DNA replication reveals a trade-off between coherence of origin activation and robustness against rereplication. *PLoS Comput Biol* **6**, e1000783, doi:10.1371/journal.pcbi.1000783 (2010).

25 Mendenhall, M. D., Jones, C. A. & Reed, S. I. Dual regulation of the yeast CDC28-p40 protein kinase complex: cell cycle, pheromone, and nutrient limitation effects. *Cell* **50**, 927-935 (1987).
26 Tyers, M., Tokiwa, G. & Futcher, B. Comparison of the Saccharomyces cerevisiae G1 cyclins: Cln3 may be an upstream activator of Cln1, Cln2 and other cyclins. *Embo j* **12**, 1955-1968 (1993).
27 Hall, D. D., Markwardt, D. D., Parviz, F. & Heideman, W. Regulation of the Cln3-Cdc28 kinase by cAMP in Saccharomyces cerevisiae. *EMBO J* **17**, 4370-4378, doi:10.1093/emboj/17.15.4370 (1998).
28 Alberghina, L., Rossi, R. L., Querin, L., Wanke, V. & Vanoni, M. A cell sizer network involving Cln3 and Far1 controls entrance into S phase in the mitotic cycle of budding yeast. *J Cell Biol* **167**, 433-443, doi:jcb.200405102 [pii] 10.1083/jcb.200405102 (2004).
29 Polymenis, M. & Schmidt, E. V. Coupling of cell division to cell growth by translational control of the G1 cyclin CLN3 in yeast. *Genes Dev* **11**, 2522-2531 (1997).
30 Schneider, B. L. *et al.* Growth rate and cell size modulate the synthesis of, and requirement for, G1-phase cyclins at start. *Mol Cell Biol* **24**, 10802-10813, doi:10.1128/MCB.24.24.10802-10813.2004 (2004).
31 Gallego, C., Garí, E., Colomina, N., Herrero, E. & Aldea, M. The Cln3 cyclin is down-regulated by translational repression and degradation during the G1 arrest caused by nitrogen deprivation in budding yeast. *EMBO J* **16**, 7196-7206, doi:10.1093/emboj/16.23.7196 (1997).
32 Barbet, N. C. *et al.* TOR controls translation initiation and early G1 progression in yeast. *Mol Biol Cell* **7**, 25-42 (1996).
33 Ghaemmaghami, S. *et al.* Global analysis of protein expression in yeast. *Nature* **425**, 737-741, doi:10.1038/nature02046 (2003).
34 Schmoller, K. M., Turner, J. J., Kõivomägi, M. & Skotheim, J. M. Dilution of the cell cycle inhibitor Whi5 controls budding-yeast cell size. *Nature* **526**, 268-272, doi:10.1038/nature14908 (2015).
35 Liu, X. *et al.* Reliable cell cycle commitment in budding yeast is ensured by signal integration. *Elife* **4**, doi:10.7554/eLife.03977 (2015).
36 Landry, B. D., Doyle, J. P., Toczyski, D. P. & Benanti, J. A. F-box protein specificity for g1 cyclins is dictated by subcellular localization. *PLoS Genet* **8**, e1002851, doi:10.1371/journal.pgen.1002851 (2012).
37 Bhaduri, S. & Pryciak, P. M. Cyclin-specific docking motifs promote phosphorylation of yeast signaling proteins by G1/S Cdk complexes. *Curr Biol* **21**, 1615-1623, doi:10.1016/j.cub.2011.08.033 (2011).
38 Tyers, M., Tokiwa, G., Nash, R. & Futcher, B. The Cln3-Cdc28 kinase complex of S. cerevisiae is regulated by proteolysis and phosphorylation. *Embo j* **11**, 1773-1784 (1992).
39 Nash, R., Tokiwa, G., Anand, S., Erickson, K. & Futcher, A. B. The WHI1+ gene of Saccharomyces cerevisiae tethers cell division to cell size and is a cyclin homolog. *EMBO J* **7**, 4335-4346 (1988).
40 Zapata, J. *et al.* PP2ARts1 is a master regulator of pathways that control cell size. *J Cell Biol* **204**, 359-376, doi:10.1083/jcb.201309119 (2014).
41 Garí, E. *et al.* Whi3 binds the mRNA of the G1 cyclin CLN3 to modulate cell fate in budding yeast. *Genes Dev* **15**, 2803-2808, doi:10.1101/gad.203501 (2001).
42 Wang, H., Garí, E., Vergés, E., Gallego, C. & Aldea, M. Recruitment of Cdc28 by Whi3 restricts nuclear accumulation of the G1 cyclin-Cdk complex to late G1. *EMBO J* **23**, 180-190, doi:10.1038/sj.emboj.7600022 (2004).
43 Vergés, E., Colomina, N., Garí, E., Gallego, C. & Aldea, M. Cyclin Cln3 is retained at the ER and released by the J chaperone Ydj1 in late G1 to trigger cell cycle entry. *Mol Cell* **26**, 649-662, doi:10.1016/j.molcel.2007.04.023 (2007).
44 Yahya, G., Parisi, E., Flores, A., Gallego, C. & Aldea, M. A Whi7-anchored loop controls the G1 Cdk-cyclin complex at start. *Mol Cell* **53**, 115-126, doi:10.1016/j.molcel.2013.11.015 (2014).
45 Kaizu, K. *et al.* A comprehensive molecular interaction map of the budding yeast cell cycle. *Mol Syst Biol* **6**, 415, doi:10.1038/msb.2010.73 (2010).
46 Mitchison, J. M. *The Biology of the Cell Cycle*. (Cambridge University Press, 1971).
47 Cross, F. R. DAF1, a mutant gene affecting size control, pheromone arrest, and cell cycle kinetics of Saccharomyces cerevisiae. *Mol Cell Biol* **8**, 4675-4684 (1988).

48 Cross, F. R. Further characterization of a size control gene in Saccharomyces cerevisiae. *J Cell Sci Suppl* **12**, 117-127 (1989).
49 McKinney, J. D., Chang, F., Heintz, N. & Cross, F. R. Negative regulation of FAR1 at the Start of the yeast cell cycle. *Genes Dev* **7**, 833-843 (1993).
50 McKinney, J. D. & Cross, F. R. FAR1 and the G1 phase specificity of cell cycle arrest by mating factor in Saccharomyces cerevisiae. *Mol Cell Biol* **15**, 2509-2516 (1995).
51 Fu, X., Ng, C., Feng, D. & Liang, C. Cdc48p is required for the cell cycle commitment point at Start via degradation of the G1-CDK inhibitor Far1p. *J Cell Biol* **163**, 21-26, doi:10.1083/jcb.200307025 jcb.200307025 [pii] (2003).
52 Jorgensen, P. *et al.* The Size of the Nucleus Increases as Yeast Cells Grow. *Mol Biol Cell* **18**, 3523-3532, doi:10.1091/mbc.E06-10-0973 (2007).
53 Wagner, M. V. *et al.* Whi5 regulation by site specific CDK-phosphorylation in Saccharomyces cerevisiae. *PLoS One* **4**, e4300, doi:10.1371/journal.pone.0004300 (2009).
54 Jorgensen, P., Nishikawa, J. L., Breitkreutz, B. J. & Tyers, M. Systematic identification of pathways that couple cell growth and division in yeast. *Science* **297**, 395-400, doi:10.1126/science.1070850 1070850 [pii] (2002).
55 Travesa, A. *et al.* Repression of G1/S transcription is mediated via interaction of the GTB motifs of Nrm1 and Whi5 with Swi6. *Mol Cell Biol* **33**, 1476-1486, doi:10.1128/mcb.01333-12 (2013).
56 Harris, M. R., Lee, D., Farmer, S., Lowndes, N. F. & de Bruin, R. A. Binding specificity of the G1/S transcriptional regulators in budding yeast. *PLoS One* **8**, e61059, doi:10.1371/journal.pone.0061059 (2013).
57 de Bruin, R. A. *et al.* Constraining G1-specific transcription to late G1 phase: the MBF-associated corepressor Nrm1 acts via negative feedback. *Mol Cell* **23**, 483-496, doi:10.1016/j.molcel.2006.06.025 (2006).
58 de Bruin, R. A., McDonald, W. H., Kalashnikova, T. I., Yates, J., 3rd & Wittenberg, C. Cln3 activates G1-specific transcription via phosphorylation of the SBF bound repressor Whi5. *Cell* **117**, 887-898, doi:10.1016/j.cell.2004.05.025 (2004).
59 Charvin, G., Oikonomou, C., Siggia, E. D. & Cross, F. R. Origin of irreversibility of cell cycle start in budding yeast. *PLoS Biol* **8**, e1000284, doi:10.1371/journal.pbio.1000284 (2010).
60 Eser, U., Falleur-Fettig, M., Johnson, A. & Skotheim, J. M. Commitment to a cellular transition precedes genome-wide transcriptional change. *Mol Cell* **43**, 515-527, doi:10.1016/j.molcel.2011.06.024 (2011).
61 Skotheim, J. M., Di Talia, S., Siggia, E. D. & Cross, F. R. Positive feedback of G1 cyclins ensures coherent cell cycle entry. *Nature* **454**, 291-296, doi:10.1038/nature07118 (2008).
62 Ferrezuelo, F. *et al.* The critical size is set at a single-cell level by growth rate to attain homeostasis and adaptation. *Nat Commun* **3**, 1012, doi:10.1038/ncomms2015 (2012).
63 Wilkinson, D. J. *Stochastic Modelling for Systems Biology*. (Chapman & Hall/CRC Mathematical & Computational Biology, 2011).
64 Allen, L. J. S. *An Introduction to Stochastic Processes with Applications to Biology*. (CRC Press/Chapman & Hall, 2011).
65 Anderson, D. F. & Kurtz, T. G. in *Design and Analysis of Biomolecular Circuits* (ed Springer-Verlag) 3-42 (2011).
66 Wang, H., Carey, L. B., Cai, Y., Wijnen, H. & Futcher, B. Recruitment of Cln3 cyclin to promoters controls cell cycle entry via histone deacetylase and other targets. *PLoS Biol* **7**, e1000189, doi:10.1371/journal.pbio.1000189 (2009).
67 Ferrezuelo, F., Colomina, N., Futcher, B. & Aldea, M. The transcriptional network activated by Cln3 cyclin at the G1-to-S transition of the yeast cell cycle. *Genome Biol* **11**, R67, doi:10.1186/gb-2010-11-6-r67 (2010).
68 Salazar, C., Brümmer, A., Alberghina, L. & Höfer, T. Timing control in regulatory networks by multisite protein modifications. *Trends Cell Biol* **20**, 634-641, doi:10.1016/j.tcb.2010.08.012 (2010).
69 Drury, L. S., Perkins, G. & Diffley, J. F. The cyclin-dependent kinase Cdc28p regulates distinct modes of Cdc6p proteolysis during the budding yeast cell cycle. *Curr Biol* **10**, 231-240 (2000).

70 Barberis, M. Sic1 as a timer of Clb cyclin waves in the yeast cell cycle--design principle of not just an inhibitor. *FEBS J* **279**, 3386-3410, doi:10.1111/j.1742-4658.2012.08542.x (2012).
71 Nash, P. *et al.* Multisite phosphorylation of a CDK inhibitor sets a threshold for the onset of DNA replication. *Nature* **414**, 514-521, doi:10.1038/35107009 (2001).
72 Yang, X., Lau, K. Y., Sevim, V. & Tang, C. Design principles of the yeast G1/S switch. *PLoS Biol* **11**, e1001673, doi:10.1371/journal.pbio.1001673 (2013).
73 Hartwell, L. H., Culotti, J., Pringle, J. R. & Reid, B. J. Genetic control of the cell division cycle in yeast. *Science* **183**, 46-51 (1974).
74 Gartner, A. *et al.* Pheromone-dependent G1 cell cycle arrest requires Far1 phosphorylation, but may not involve inhibition of Cdc28-Cln2 kinase, in vivo. *Mol Cell Biol* **18**, 3681-3691 (1998).
75 Doncic, A. & Skotheim, J. M. Feedforward regulation ensures stability and rapid reversibility of a cellular state. *Mol Cell* **50**, 856-868, doi:10.1016/j.molcel.2013.04.014 (2013).
76 Warner, J. R. The economics of ribosome biosynthesis in yeast. *Trends Biochem Sci* **24**, 437-440 (1999).
